# Precursor‐Directed Synthesis of Apoptosis‐Initiating *N*‐Hydroxyalkyl Phenylbenzoisoquinolindione Alkaloids

**DOI:** 10.1002/open.202200157

**Published:** 2022-12-07

**Authors:** Yu Chen, Hans‐Martin Dahse, Christian Paetz, Bernd Schneider

**Affiliations:** ^1^ Max-Planck-Institute for Chemical Ecology NMR/Biosynthesis Group Hans-Knöll-Straße 8 07745 Jena Germany; ^2^ Institute of Botany Jiangsu Province and Chinese Academy of Sciences No.1 Qianhu Houcun Xuanwu District 210014 Nanjing P. R. China; ^3^ Leibniz Institute for Natural Product Research and Infection Biology Hans Knöll Institute (Leibniz-HKI) Beutenbergstraße 11a 07745 Jena Germany

**Keywords:** antiproliferative effect, apoptosis, cytotoxicity, phenylbenzoisoquinolindione, precursor-directed synthesis

## Abstract

A precursor‐directed approach to access *N*‐hydroxyalkyl phenylbenzoisoquinolindiones (PBIQs) has been developed. Incubation of plant material of *Xiphidium caeruleum* with hydroxylamines of various chain lengths (C_2_, C_4_, C_6_, C_8_, C_10_ and C_12_) resulted in 11 new 5‐hydroxy‐ and 5‐methoxy PBIQs with different *N*‐hydroxyalkyl side chain lengths. The antiproliferative effect and the cytotoxicity against HUVEC, K‐562, and HeLa cell lines of 26 previously reported PBIQs and the 11 newly synthesized *N*‐hydroxyalkyl PBIQs was determined for the first time. The results revealed that introducing long‐chain *N*‐aliphatic amine moieties improved the antiproliferative effect and cytotoxicity of PBIQs when compared to derivatives with *N*‐amino acids as side chains.

## Graphical abstract

**Direct incubation of** ***Xiphidium caeruleum*** **plant material** with ω‐amino alcohols followed by extraction and purification results in *N*‐hydroxyalkyl phenylbenzoisoquinolindiones (PBIQs). Long‐chain derivatives show good antiproliferative effects together with low cytotoxicity.

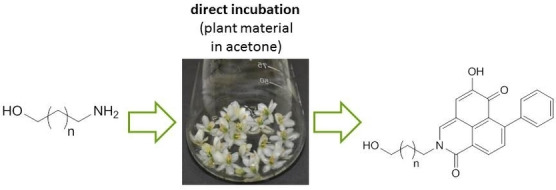

## Introduction

**Scheme 1:**
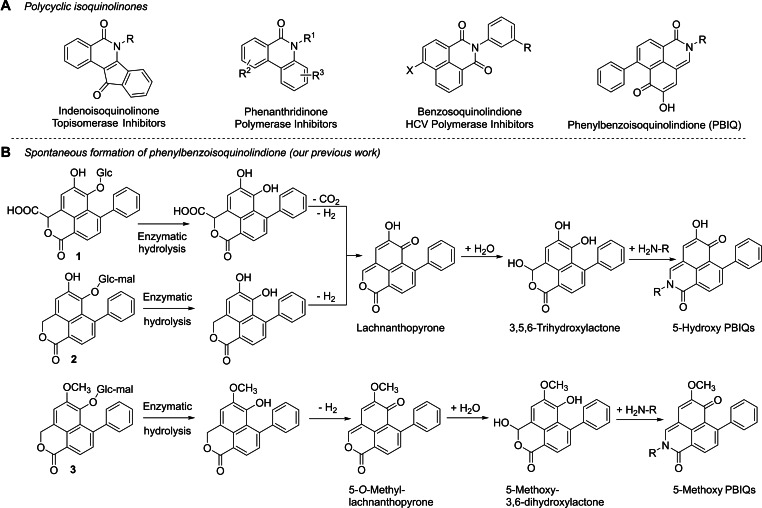
Structures of some polycyclic isoquinolinone alkaloids (A) and pathway of conversion of phenylbenzoisochromenone glucosides **1–3** (PBICs) to phenylbenzoisoquinolindiones (PBIQs) (B) (Chen et al., 2018).^[13]^ HCV: hepatitis C virus.

Isoquinolines, which represent one of the largest groups of naturally occurring alkaloids, show a wide spectrum of biological activities.^[1]^ Polycyclic isoquinolinones constitute an important subclass of isoquinoline alkaloids, and their scaffold was recognized as an important structural feature for some antitumor and/or antiviral drug candidates, such as indeno[1,2‐c]isoquinolinones,^[2]^ 1*H*‐benzo[*de*]isoquinoline‐1,3(2*H*)‐diones,^[3]^ and phenanthridinone (Scheme 1).^[4]^ To synthesize isoquinolones, various approaches have been developed over the years, such as transition‐metal‐catalyzed C−H activation,^[5]^ annulation,^[6]^ or insertion.^[7]^ Recently, metal‐free C−H activation^[8]^ and annulation^[9]^ were reported. Phenylbenzoisoquinolindiones (PBIQs) are a group of special alkaloids consisting of a nitrogen‐containing tricyclic scaffold and a laterally attached phenyl ring. PBIQs have been reported only in *Xiphidium caeruleum* ^[10]^ and from two other Haemodoraceae plants, *Lachnanthes tinctoria* ^[11]^ and *Wachendorfia thyrsiflora*.^[12]^

PBIQs in *X. caeruleum* were derived from a series of enzymatic hydrolyses and spontaneous conversions from three native phenylbenzoisochromenone (PBIC) glucosides, **1**–**3**, and plant proteinogenic amino acids (Scheme 1).^[13]^ Specifically, 5‐hydroxy PBIQs were generated from **1** and **2** through the common intermediate lachnanthopyrone, and 5‐methoxy PBIQs were formed from **3** through the intermediate 5‐*O*‐methyl lachnanthopyrone. Using the formation mechanism above (Scheme 1), we successfully prepared a series of new PBIQs by precursor‐directed synthesis. The strong affinity of the hydroxylactone intermediates (Scheme 1) towards primary amines and the antimicrobial activity of some PBICs provided support for a novel phenylphenalenone‐based plant defense mechanism.^[13]^ The reaction of biogenic amino compounds’ *N*‐termini with the PBICs forms the basis of this defense mechanism. The antiproliferative and cytotoxic activities of the previously reported PBIQs, and their potential pharmacological role, were evaluated for the first time in the present work. Preliminary results prompted us to synthesize a series of *N*‐hydroxyalkyl‐substituted PBIQs. Antitumor bioassays suggest that these compounds can inhibit the proliferation of human tumor cells.

## Results and Discussion

### Antiproliferative and cytotoxicity tests of PBIQs

**Figure 1:**
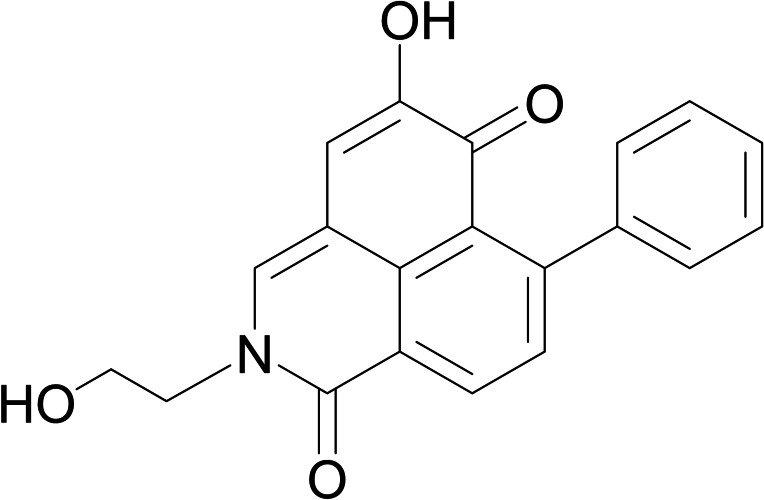
Structure of (2‐hydroxyethyl)‐lachnanthopyridone (**30**).

The frequency with which an isoquinolinone unit occurred in cancerostatic alkaloids motivated us to assess the antiproliferative and cytotoxic properties of our previously reported PBIQs (compounds **4**–**29**) (see Supporting Information Table ST1).[^10a^, ^13^] The 26 compounds, which were obtained by precursor‐directed synthesis using various amino acids,^[13]^ were assayed in a screening test against the human HUVEC, K‐562, and HeLa cell lines. The results showed that none of the amino‐acid‐side‐chain‐containing PBIQs (**4**–**27**), the bis‐PBIQ (**28**), and the thiazolo‐fused PBIQ (**29**) had any effect on the growth of the tested human tumor cell lines. However, (2‐hydroxyethyl)‐lachnanthopyridone (**30**, Figure 1), the first reported PBIQ,^[11b]^ slowed the growth of the HUVEC and K‐562 cells and showed medium cytotoxicity against the HeLa cells (Table 1).

**Table 1:** Bioassay data for (2‐hydroxyethyl)‐lachnanthopyridone (**30**).

Antiproliferative effect		Cytotoxicity
HUVEC (GI_50_)	K‐562 (GI_50_)	HeLa (CC_50_)
38.3 μg mL^−1^	29.4 μg mL^−1^	49.3 μg mL^−1^

### Precursor‐directed synthesis of N‐hydroxyalkyl PBIQ alkaloids

Both the negative bioassay results obtained for compounds **4**–**29** (Supporting Information Table ST1) and the positive result of the preliminary bioactivity tests for compound **30** suggested that the *N*‐hydroxyalkyl group may play an important role in inhibiting the proliferation of cancer cells. Changes in the alkyl chain length of some known chemopreventive and antiproliferative agents help modulating their overall lipophilicity and hence their ability to penetrate cell membranes, which improves their biological activities. Examples are isothiocyanates,^[14]^ paradol derivatives,^[15]^ alkylaminophenols,^[16]^ and novobiocin analogues.^[17]^ Therefore, we focused on PBIQs with *N*‐alkyl chains of various lengths and examined their activities against cancer cell lines.

**Figure 2:**
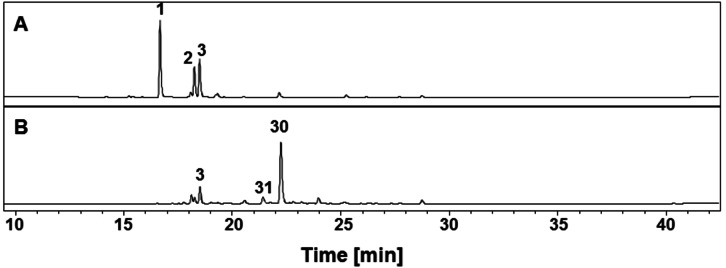
HPLC‐HRESIMS profile (*λ*=254 nm) of the methanol extract of raw plant material of *X. caeruleum* (A) and the products of incubating plant material with ethanolamine (B) For HPLC‐HRESIMS chromatograms of incubations with the *N*‐hydroxylamines of chains C_4_ to C_12_, see Supporting Information Figure SF1.

The relatively high levels of PBIC glucosides **1**–**3** (Figure 2A) in the green aerial parts of *X. caeruleum* have proved to be excellent starting material for producing PBIQs by precursor‐directed biosynthesis.^[13]^ Suspended in acetone, the plant material was incubated with commercially available ω‐hydroxylamines having alkyl chains of various lengths; the goal was to synthesize *N*‐hydroxyalkyl PBIQs **31**–**41**. As demonstrated in Scheme 1, green leaves and stems of *X. caeruleum* were washed, homogenized, and transferred to an Erlenmeyer flask containing acetone. After each suspension received its respective ω‐hydroxylamine, incubation proceeded at room temperature for 48 h. An example is illustrated in Figure 2A and B: Analyzing the suspensions before and after incubation, we noted that the major peaks corresponding to **1**–**3** almost disappeared, whereas a major new peak, here assigned to 5‐hydroxy PBIQ **30**, and a small new peak, assigned to a new 5‐methoxy PBIQ **31**, emerged. Interestingly, a color change of the incubation solution from green to brown indicated that most of the PBIC glucosides **1**–**3** were transformed to the orange PBIQs, among which PBIQs **30** and **31** were derived from the exogenous ethanolamine. After chromatographic separation, the major 5‐hydroxy PBIQ **30**, derived from PBIC glucosides **1** and **2**, and the minor 5‐methoxy PBIQ **31**, derived from **3**, were obtained. The ratio of **30** and **31** in the plant material was in accordance with the initial contents of precursors **1**–**3**. Similarly, ten other 5‐hydroxy and 5‐methoxy PBIQs (**32**–**41**) with different lengths of the *N*‐hydroxyalkyl side chain (C_4_, C_6_, C_8_, C_10_, C_12_) were synthesized by this method (Scheme 1). The structures of the new PBIQs (**31**–**41**) were elucidated by nuclear magnetic resonance (NMR) spectroscopy and high‐resolution mass spectrometry (HRMS) (see Supporting Information Tables ST5–ST7).

### Analytical properties

Phenylbenzoisochromenone (PBIC) glucosides **1**–**3**, precursors of phenylbenzoisoquinolindiones (PBIQs), are major phenylphenalenone metabolites in the aerial green parts of *X. caeruleum*. We aimed to determine the concentration of compounds **1**–**3** in the plant material used for incubation experiments in order to calculate the yield of the resulting PBIQs. Quantitative analyses were performed by a high performance liquid chromatography (HPLC) method for isolation of compounds **1**–**3** as external standards. Fresh leaf material of *X. caeruleum* contained 0.70±0.09 mg g^−1^ of compound **1**, 0.31±0.01 mg g^−1^ of **2**, and 0.33±0.01 mg g^−1^ of **3**, respectively (see Supporting Information Table ST3). The yields of PBIQs **30**–**41** were determined by calculation based on the content of **1–3** in the leaf material used for incubation (Table 2 and Supporting Information Table ST4). The results showed that the yield of 5‐hydroxy PBIQs was slightly higher than that of the corresponding 5‐methoxy PBIQs, perhaps because 3,5,6‐trihydroxylactone is more reactive than 5‐methoxy‐3,6‐dihydroxylactone (for structures, see Table 2). Although HPLC‐HRMS analyses of the incubation solutions (Supporting Information Figure SF1) indicated that *N*‐hydroxyalkyl PBIQs were the major reaction products, their yields ranged only between 12 % and 28 %. Two possibilities may account for the low yields: either a part of precursors **1**–**3** reacted with endogenous amino acids, leading to fewer of the desired PBIQs; or a part of the active hydroxylactone reacted with the *N*‐terminal of endogenous proteins, which did not dissolve in the incubation solutions.

**Table 2:** Chemical structure, synthesis, antiproliferative effect, and cytotoxicity of *N*‐hydroxy‐alkyl PBIQs (GI_50_ and CC_50_ ±S.D., four technical replicates).

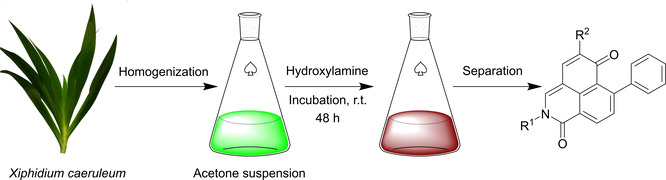							
Hydroxylamine	Product			Yield [%]	Antiproliferative effect		Cytotoxicity
	No.	R^1^	R^2^		HUVEC GI_50_ [μg mL^−1^]	K‐562 GI_50_ [μg mL^−1^]	HeLa CC_50_ [μg mL^−1^]
Ethanolamine	**30**	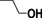	OH	23	28.7 (±1.2)	11.9 (±0.7)	39.8 (±0.9)
	**31**	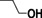	OCH_3_	14	43.4 (±1.0)	49.5 (±0.6)	42.2 (±2.9)
Butanolamine	**32**	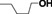	OH	17	13.6 (±1.2)	6.0 (±0.5)	30.3 (±0.6)
	**33**	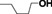	OCH_3_	16	34.2 (±1.7)	29.6 (±1.3)	41.3 (±0.2)
6‐Amino‐1‐hexanol	**34**	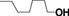	OH	28	6.4 (±0.9)	3.4 (±0.2)	19.2 (±1.2)
	**35**	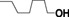	OCH_3_	26	15.0 (±0.5)	16.9 (±2.5)	27.7 (±2.1)
8‐Amino‐1‐octanol	**36**	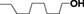	OH	19	9.0 (±1.0)	5.2 (±0.3)	>50
	**37**	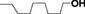	OCH_3_	17	6.8 (±0.3)	7.0 (±1.6)	16.4 (±0.2)
10‐Amino‐1‐decanol	**38**	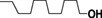	OH	20	6.2 (±0.2)	2.2 (±0.05)	>50
	**39**	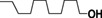	OCH_3_	12	5.7 (±0.4)	3.2 (±0.2)	8.3 (±0.3)
12‐Amino‐1‐dodecanol	**40**	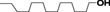	OH	17	>50	2.1 (±0.2)	>50
	**41**	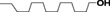	OCH_3_	12	7.0 (±0.3)	2.9 (±0.6)	12.8 (±1.0)
Camptothecin					–	0.1	0.2

### Antiproliferative and cell toxicity data

The two series of *N*‐hydroxyalkyl PBIQs, which differed in the substituent in position 5 (R^2^=OH or R^2^=OCH_3_; Table 2), were tested for their biological activities. Generally, moderate antiproliferative activities of the compounds of the two series were determined against HUVEC and K‐562 cells. Compounds **30**, **32**, **34**, **36**, **38**, and **40**, all of which had a 5‐OH group, showed a trend towards increasing activity with increasing *N*‐hydroxyalkyl chain lengths (Table 2). For example, short‐chain compound **30** (R^1^=C2; R^2^=OH) showed GI_50_ values of 28.7 μg mL^−1^ (HUVEC) and 11.9 μg mL^−1^ (K‐562) in the antiproliferative test. Activity values of long‐chain compound **38** (R^1^=C10; R^2^=OH) were 6.2 μg mL^−1^ (HUVEC) and 2.2 μg mL^−1^ (K‐562). The trend towards increasing activity with extended *N*‐hydroxyethyl chain length was also observed for **31**, **33**, **35**, **37**, **39**, and **41**, all of which had a 5‐OCH_3_ group: short‐chain compound **31** (R^1^=C2; R^2^=OH) showed GI_50_ values of only 43.4 μg mL^−1^ (HUVEC) and 49.5 μg mL^−1^ (K‐562), whereas the GI_50_ values of long‐chain compound **41** (R^1^=C12; R^2^=OH) were 7.0 μg mL^−1^ (HUVEC) and 2.9 μg mL^−1^ (K‐562). Most of the *N*‐hydroxyalkyl PBIQs that exhibited medium cytotoxicity against HeLa cells also showed a trend towards increasing activity in compounds with extended *N*‐hydroxyethyl chain lengths (Table 2). However, some compounds (**36**, **38**, and **40**) failed to show any cytotoxicity. The antiproliferative and cytotoxicity data from all tested compounds are presented in Table 2, and the corresponding dose‐response curves are shown in the Supporting Information Figures SF3 to SF5.

### Apoptosis data

A relatively strong antiproliferative activity against leukemic K‐562 (see Supporting Information Figure SF4) led us to check if the compounds induced apoptosis. Two different assays were performed: Cell Death Detection ELISA^PLUS^ and Annexin V FITC/Fixable Viability Dye eFluor780 flow cytometric assay. The first used mouse monoclonal antibodies directed against mono‐ and oligo‐nucleosomes in the cytoplasmatic fraction of the cell lysates to quantify apoptosis. The second, based on flow cytometry, detected early and late apoptotic cells as a result of ongoing apoptosis. The results of Cell Death Detection ELISA^PLUS^ demonstrated that all PBIQs induce apoptosis in leukemic K‐562. Compared to the speed of induction in the positive control, camptothecin, the apoptosis induction of the new PBIQs proceeded more slowly. Although at a lower concentration, camptothecin induced apoptosis already within 4 h. (see Supporting Information Figure SF6).

## Conclusions

Employing an innate plant defense mechanism in *Xiphidium caeruleum* (Aubl.), we synthesized a series of *N*‐hydroxyalkyl PBIQs by *in vitro* incubation experiments. The compounds showed activity against human cancer cell lines where they induced apoptosis. The mechanism of cytotoxicity and the cellular targets of the *N*‐hydroxyalkyl PBIQs remain unclear. However, we could show that phenylphenalenone‐type compounds like PBIQs may serve as possible lead structures in anti‐cancer drug development.

## Experimental Section

### General experimental procedures

NMR spectra (^1^H NMR, ^1^H‐^1^H SELTOCSY, ^13^C NMR, ^1^H‐^1^H COSY, ^1^H‐^1^H ROESY, ^1^H‐^13^C HSQC and ^1^H‐^13^C HMBC) were measured either on a Bruker Avance III HD 500 NMR spectrometer (operating at 500.13 MHz for ^1^H and 125.75 MHz for ^13^C; 5 mm TCI cryoprobe) or on a Bruker Avance III HD 700 NMR spectrometer (operating at 700.13 MHz for ^1^H and 175.75 MHz for ^13^C; 1.7 mm TCI microcryoprobe) (Bruker Biospin, Karlsruhe, Germany), controlled by Bruker TopSpin ver. 3.5 and ver. 3.2, respectively. Standard pulse programs as implemented in Bruker TopSpin were used. Data processing was accomplished using Bruker TopSpin. The residual solvent signals were used for referencing spectra in the ^1^H and ^13^C dimensions.

Chromatographic analyses and separations were conducted on an Agilent Infinity 1260 HPLC consisting of a quaternary pump (G1311B), autosampler (G1367E), column oven (G1316A), and diode array detector (G1315D) (Agilent Analytik GmbH, Waldbronn, Germany). The devices were controlled using Bruker Hystar ver. 3.2 (Bruker Biospin, Karlsruhe, Germany). Semipreparative HPLC separations were carried out using a Shimadzu Prominence HPLC system, controlled by the Shimadzu LCSolution ver. 1.21, and consisting of a DGU‐20As degasser, LC‐20AT gradient pump, SIL‐10AP autosampler, CTO‐20A column oven, SPD‐20 A UV detector, FRC‐10A fraction collector and CBM‐20A system controller (Shimadzu Deutschland GmbH, Duisburg, Germany). For conducting micro‐preparative separation (HPLC‐SPE), the Agilent Infinity 1260 HPLC was coupled to a Bruker/Spark Holland Prospekt 2 SPE fraction collector (Bruker Biospin, Karlsruhe, Germany/Spark Holland, Emmen, The Netherlands) equipped with HySphere resin GP cartridges (10 μm; 10×2 mm) to trap selected peaks. Depending on the flow rate used for chromatographic separation, a four‐fold flow of water was added post‐column by a make‐up pump (Knauer, Berlin, Germany) in order to reduce the eluotropic capacity of the eluent. After chromatographic loading, cartridges were dried with a stream of N_2_ gas for 15 min before being eluted with MeCN into 2 mL Eppendorf micro‐reaction vessels (Eppendorf, Wesseling‐Berzdorf, Germany) for further analysis by MS or NMR.

The following columns and gradient conditions were used for chromatographic separations:

- HPLC‐UV quantitative analysis, Agilent 1100 system. Merck Purospher STAR RP‐18e (5 μm; 250×4.6 mm; Merck, Darmstadt, Germany) using H_2_O (0.1 % (*v*/*v*) formic acid (FA), solvent A) and MeCN (0.1 % (*v*/*v*) FA, solvent B) at 30 °C with a flow rate of 0.8 mL min^−1^. Linear binary gradient: 0–5 min 18–25 % B, 5–35 min 25 % B, 35–40 min 25–30 % B, 40–48 min 100 % B, and 48–55 min 18 % B.
- HPLC‐PDA‐HRESIMS measurements, Agilent 1260 system. Zorbax C18 column (3.5 μm; 150×4.6 mm; Agilent, St Louis, MO, USA) with a constant flow rate of 500 μL min^−1^ at 25 °C, binary solvent system of H_2_O (solvent A) and MeCN (solvent B), both containing 0.1 % (*v*/*v*) FA. Linear binary gradient (1): 0–2 min 5 % B, 40 min 100 % B, 40–55 min 100 % B, and 55–60 min 5 % B; (2): 0–23 min 35–100 % B, 23–26 min 100 % B, and 26–30 min 35 % B.
- Semipreparative separation, Shimadzu Prominence HPLC system. MN Nucleodur C‐18 HTEC column (5 μm; 250×10 mm; Macherey‐Nagel, Düren, Germany) at 25 °C with a flow rate of 3.5 mL min^−1^. Binary solvent system of 0.1 % FA (*v*/*v*) in H_2_O (solvent A) and 0.1 % FA (*v*/*v*) in MeOH (solvent B). Linear binary gradient (1): 0–5 min 55–60 % B, 5–25 min 60 % B, 25–30 min 60–100 % B, 30–38 min 100 % B, and 38–45 min 55 % B; (2): 0–20 min 70 % B, 20–25 min 70–100 % B, 25–30 min 100 % B, and 30–35 min 70 % B; (3): 0–10 min 70–80 % B, 10–20 min 80 % B, 20–27 min 100 % B, and 27–35 min 70 % B; (4): 0–10 min 75–85 % B, 10–20 min 85 % B, 20–27 min 100 % B, and 27–35 min 75 % B; (5): 0–10 min 85–90 % B, 10–20 min 90 % B, 20–27 min 100 % B, and 27–35 min 85 % B.

For HRESIMS measurements, the Agilent Infinity 1260 HPLC was coupled to a Bruker Compact OTOF mass spectrometer (Bruker Daltonics, Bremen, Germany), controlled by Bruker Compass (ver. 1.9)/Bruker OTOFControl ver. 4.0 (Bruker Daltonics, Bremen, Germany). The samples were measured in positive ionization mode in the mass range *m/z* 50 to 1300 using 30,000 m/Δm resolving power.

Purities of hydroxylamines (TCI Deutschland GmbH, Eschborn, Germany) were as follows: ethanolamine >99 %, butanolamine >98 %, 6‐amino‐1‐hexanol >97 %%, 8‐amino‐1‐octanol >98.0 %, 10‐amino‐1‐decanol >98.0 %, and 12‐amino‐1‐dodecanol >98.0 %. Other reagents and solvents were purchased from Sigma‐Aldrich, Deisenhofen, Germany, and used without further purification.

### Plant material

Twelve branches (600 g, fresh weight) of *Xiphidium caeruleum* Aubl. plants grown in soil were collected from the greenhouse of the botanical garden of the Friedrich Schiller University, Jena, Germany, in March 2017. After the red senescent parts were removed, the green leaves and stems were washed with distilled water, dried, and cut into 4–5 cm pieces. From the pooled plant material, three samples (18 g) were taken for quantitative analysis, and the remaining material was used to perform incubation experiments.

### Quantification of PBIC glucosides 1–3 in plant materials

From the fresh plant material, three samples of 18 g (fresh weight) were frozen in liquid nitrogen and freeze‐dried. The lyophilized plant material was weighed, transferred into three 50 mL polypropylene centrifuge tubes, and powdered by shaking in the presence of steel beads (2 mm diameter) in a paint shaker SO‐10 m (Fluid Management, Sassenheim, The Netherlands) for 2 min. Approximately 30 mg lyophilized powder of each sample was weighed and transferred into a 2 mL Eppendorf tube, to which 1.5 mL of methanol was added. The suspension was vortexed (1 min) and sonicated (3 min) at room temperature. Subsequently, the homogenate was centrifuged at 13,000 rpm using an Eppendorf centrifuge 5415R (Eppendorf, Wesseling‐Berzdorf, Germany) for 3 min at 20 °C. Supernatants were collected, and the remaining debris were extracted two more times with methanol (1.5 mL). Afterwards, the pooled supernatants were dried using nitrogen gas and reconstituted in 1 mL of methanol. The solution was then filtered and subjected to HPLC analysis (method A) and HPLC‐HRMS analysis (method B, gradient 1). The average of the UV signal (254 nm) integral values of three injections from each of the three samples was used to construct calibration curves (see Supporting Information Figure SF2). These were obtained as follows: Stock solutions of the previously isolated glucosides **1**–**3** ^[13]^ (purity >98 %, checked by ^1^H NMR), were prepared in methanol and diluted to appropriate concentration ranges. A calibration curve for each compound was constructed with five different concentrations in triplicate by plotting the peak area (*y*) versus concentration (*x*). The linearity for each compound was evaluated by the correlation coefficient (r^2^≥0.9982). The results are presented in the Supporting Information Table ST2. The limits of detection (LOD) and quantification (LOQ) at 254 nm were estimated by means of the baseline noise method.

### Precursor‐directed synthesis of N‐hydroxyalkyl PBIQ alkaloids

Six hydroxylamines (see Supporting Information Table ST4) were used for precursor‐directed synthesis as follows: pieces of leaves of soil‐cultured *X. caeruleum* plants were homogenized by a blender (MaxoMixx, Bosch, Germany) and transferred into Erlenmeyer flasks. Each flask was filled with acetone to about 5 times the volume of the leaf suspension, then covered with perforated aluminum foil to expose the contents to air. To the suspension, the ω‐hydroxylamine (see Supporting Information Table ST4) in acetone (10–50 mL, depending on solubility) was added. After being shaken for 2 d at ambient temperature, the suspension was filtered through degreased cotton wool. The solvent was removed by means of a rotary evaporator, reconstituted with distilled water, and passed through preconditioned Chromabond HR−X SPE cartridges (Macherey‐Nagel, Düren, Germany). After being washed with water, the loaded cartridges were eluted with MeCN. The MeCN eluates were evaporated using N_2_ gas, reconstituted with acetone, and subjected to HPLC‐HRMS analysis (method B, gradient 1). Purification of the compounds was accomplished by semi‐preparative HPLC using method C with the following conditions: compounds **30**–**33**, gradient 1; **34**–**35**, gradient 2; **36** –**37**, gradient 3; **38**–**39**, gradient 4; and **40**–**41**, gradient 5. For yields, see Supporting Information Table ST4. The structures of the new PBIQs (**31**–**41**) were determined by NMR spectroscopy and HRMS (see Supporting Information Tables ST5 to ST7 and Schemes SS1 to SS12).

***2‐(2′′‐Hydroxyethyl)‐5‐methoxy‐7‐phenyl‐2H‐benzo[de]isoquinoline‐1,6‐dione (31)***: Orange powder. HPLC‐PDA‐HRESIMS gradient 2, *t*_R_ 7.5 min; UV (MeCN‐H_2_O) *λ*_max_ 206, 240, 320, 434 nm; ^1^H NMR data, see Supporting Information Table ST5; ^13^C NMR data, see Supporting Information Table ST7; HRESIMS *m/z* 348.1237 [M+H]^+^ (calcd for C_21_H_18_NO_4_, 348.1230).

***2‐(4′′‐Hydroxybutyl)‐5‐hydroxy‐7‐phenyl‐2H‐benzo[de]isoquinoline‐1,6‐dione (32)***: Orange powder. HPLC‐PDA‐ESIMS gradient 2, *t*_R_ 9.4 min; UV (MeCN‐H_2_O) *λ*_max_ 206, 238, 322, 442 nm; ^1^H NMR data, see Supporting Information Table ST5; ^13^C NMR data, see Supporting Information Table ST7; HRESIMS *m/z* 362.1394 [M+H]^+^ (calcd for C_22_H_20_NO_4_, 362.1387).

***2‐(4′′‐Hydroxybutyl)‐5‐methoxy‐7‐phenyl‐2H‐benzo[de]isoquinoline‐1,6‐dione (33)***: Orange powder. HPLC‐PDA‐HRESIMS gradient 2, *t*_R_ 8.4 min; UV (MeCN‐H_2_O) *λ*_max_ 196, 238, 320, 434 nm; ^1^H NMR data, see Supporting Information Table ST5; ^13^C NMR data, see Supporting Information Table ST7; HRESIMS *m/z* 376.1541 [M+H]^+^ (calcd for C_23_H_22_NO_4_, 376.1543).

***2‐(6′′‐Hydroxyhexyl)‐5‐hydroxy‐7‐phenyl‐2H‐benzo[de]isoquinoline‐1,6‐dione (34)***: Orange powder. HPLC‐PDA‐HRESIMS gradient 2, *t*_R_ 12.3 min; UV (MeCN‐H_2_O) *λ*_max_ 224, 324, 444 nm; ^1^H NMR data, see Supporting Information Table ST5; ^13^C NMR data, see Supporting Information Table ST7; HRESIMS *m/z* 390.1708 [M+H]^+^ (calcd for C_24_H_24_NO_4_, 390.1700).

***2‐(6′′‐Hydroxyhexyl)‐5‐methoxy‐7‐phenyl‐2H‐benzo[de]isoquinoline‐1,6‐dione (35)***: Orange powder. HPLC‐PDA‐HRESIMS gradient 2, *t*_R_ 10.9 min; UV (MeCN‐H_2_O) *λ*_max_ 206, 238, 320, 434 nm; ^1^H NMR data, see Supporting Information Table ST5; ^13^C NMR data, see Supporting Information Table ST7; HRESIMS *m/z* 404.1863 [M+H]^+^ (calcd for C_25_H_26_NO_4_, 404.1856).

***2‐(8′′‐Hydroxyoctyl)‐5‐hydroxy‐7‐phenyl‐2H‐benzo[de]isoquinoline‐1,6‐dione (36)***: Orange powder. HPLC‐PDA‐HRESIMS gradient 2, *t*_R_ 15.3 min; UV (MeCN‐H_2_O) *λ*_max_ 206, 218, 238, 324, 444 nm; ^1^H NMR data, see Supporting Information Table ST5; ^13^C NMR data, see Supporting Information Table ST7; HRESIMS *m/z* 418.2030 [M+H]^+^ (calcd for C_26_H_28_NO_4_, 418.2013).

***2‐(8′′‐Hydroxyoctyl)‐5‐methoxy‐7‐phenyl‐2H‐benzo[de]isoquinoline‐1,6‐dione (37)***: Orange powder. HPLC‐DAD‐ESIMS gradient 2, *t*_R_ 14.1 min; UV (MeCN‐H_2_O) *λ*_max_ 206, 238, 320, 434 nm; ^1^H NMR data, see Supporting Information Table ST6; ^13^C NMR data, see Supporting Information Table ST7; HRESIMS *m/z* 432.2171 [M+H]^+^ (calcd for C_27_H_30_NO_4_, 432.2169).

***2‐(10′′‐Hydroxydecyl)‐5‐hydroxy‐7‐phenyl‐2H‐benzo[de]isoquinoline‐1,6‐dione (38)***: Orange powder. HPLC‐PDA‐HRESIMS gradient 2, *t*_R_ 18.7 min; UV (MeCN‐H_2_O) *λ*_max_ 208, 234, 324, 444 nm; ^1^H NMR data, see Supporting Information Table ST6; ^13^C NMR data, see Supporting Information Table ST7; HRESIMS *m/z* 446.2326 [M+H]^+^ (calcd for C_28_H_32_NO_4_, 446.2326).

***2‐(10′′‐Hydroxydecyl)‐5‐methoxy‐7‐phenyl‐2H‐benzo[de]isoquinoline‐1,6‐dione (39)***: Orange powder. HPLC‐DAD‐ESIMS gradient 2, *t*_R_ 17.6 min; UV (MeCN‐H_2_O) *λ*_max_ 204, 238, 320, 434 nm; ^1^H NMR data, see Supporting Information Table ST6; ^13^C NMR data, see Supporting Information Table ST7; HRESIMS *m/z* 460.2482 [M+H]^+^ (calcd for C_29_H_34_NO_4_, 460.2482).

***2‐(12′′‐Hydroxydodecyl)‐5‐hydroxy‐7‐phenyl‐2H‐benzo[de]isoquinoline‐1,6‐dione (40)***: Orange powder. HPLC‐DAD‐ESIMS gradient 2, *t*_R_ 22.3 min; UV (MeCN‐H_2_O) *λ*_max_ 206, 242, 324, 444 nm; ^1^H NMR data, see Supporting Information Table ST6; ^13^C NMR data, see Supporting Information Table ST7; HRESIMS *m/z* 474.2649 [M+H]^+^ (calcd for C_30_H_36_NO_4_, 474.2639).

***2‐(12′′‐Hydroxydodecyl)‐5‐methoxy‐7‐phenyl‐2H‐benzo[de]isoquinoline‐1,6‐dione (41)***: Orange powder. HPLC‐DAD‐ESIMS gradient 2, *t*_R_ 21.2 min; UV (MeCN‐H_2_O) *λ*_max_ 204, 236, 320, 434 nm; ^1^H NMR data, see Supporting Information Table ST6; ^13^C NMR data, see Supporting Information Table ST7; HRESIMS *m/z* 488.2806 [M+H]^+^ (calcd for C_31_H_38_NO_4_, 488.2795).

### Cell toxicity

To determine cell toxicity and antiproliferative activity, PBIQs **30**–**41** were dissolved in dimethylsulfoxide (DMSO, conc. 10 mg mL^−1^). Five replicates were investigated for each assay. Cell toxicity assays were conducted using a HeLa cell line (DSM ACC 57), and antiproliferative activity was assayed using HUVEC (ATCC CRL‐1730) and K‐562 cell lines (DSM ACC 10). The test substances were dissolved in DMSO before being diluted in the respective cell culture medium to concentrations of between 1 and 100 μg mL^−1^. The adherent cells were harvested in the logarithmic growth phase after soft trypsinization, using 0.25 % trypsin in phosphate‐buffered saline containing 0.02 % ethylenediaminetetraacetic acid. For each experiment, approximately 10,000 cells were seeded with 0.1 mL culture medium per well of a 96‐well microplate. HeLa cells were pre‐incubated for 48 h prior to the application of the test compounds to give subconfluent monolayers. Incubation was then conducted in a humidified atmosphere at 37 °C and 5 % CO_2_. In the case of K‐562 cells, the antiproliferative effect was determined using the CellTiter‐Blue1 assay.^[18]^ The adherent HUVEC and HeLa cells were fixed by glutaraldehyde (MERCK 1.04239.0250) and stained with a 0.05 % solution of methylene blue (SERVA 29198) for 10 min. After gentle washing, the dye was eluted with 0.2 mL of 0.33 n HCl from the wells. The optical densities were measured at 660 nm in a SUNRISE microplate reader (Tecan Trading AG, Männedorf, Switzerland).

### Apoptosis assays

Cell Death Detection ELISA^PLUS^ (Merck, Darmstadt, Germany) was performed as described earlier.^[19]^ To detect early and late apoptotic cells by flow cytometry, the described protocols for Annexin V FITC (Biolegend, cat. no. 640906) and Fixable Viability Dye eFluor780 (eBioscience, cat. no. 65–0865‐14) were used.

## Supporting Information Summary

An overview of the compounds used for the bioassays together with their analytical data (MS and NMR) is provided in the Supporting Information. Furthermore, the results of the bioassays in graphical form and microscopic images of cells treated with PBIQs are given.

## Conflict of interest

The authors declare no conflict of interest.

1

## Supporting information

As a service to our authors and readers, this journal provides supporting information supplied by the authors. Such materials are peer reviewed and may be re‐organized for online delivery, but are not copy‐edited or typeset. Technical support issues arising from supporting information (other than missing files) should be addressed to the authors.

Supporting Information

## Data Availability

The data that support the findings of this study are available in the supplementary material of this article.
