# Supplementary material for: Precursor‐Directed Synthesis of Apoptosis‐Initiating N‐Hydroxyalkyl Phenylbenzoisoquinolindione Alkaloids
Source: ChemistryOpen. 2022 Dec 7;11(12):e202200157. doi: 10.1002/open.202200157 (PMC9728486; doi:10.1002/open.202200157)
Supplement: Supplementary file 1 — Supporting Information [file OPEN-11-e202200157-s001.pdf]

ChemistryOpen

Supporting Information

Precursor-Directed Synthesis of Apoptosis-Initiating *N*-Hydroxyalkyl Phenylbenzoisoquinolindione Alkaloids

Yu Chen, Hans-Martin Dahse, Christian Paetz,* and Bernd Schneider

Table ST1. Chemical structures of previously reported PBIQs **4** - **29**

Figure SF1. HPLC-HRESIMS chromatograms of PBIQs **32** to **41** after incubation of plant material with hydroxylamines

Figure SF2. HPLC-UV chromatograms of a raw plant material extract and standard compounds **1** - **3**

Table ST2. Calibration curves and test ranges of standard compounds **1** - **3**

Table ST3. Concentration of compounds **1** – **3** in above-ground plant material of *X. caeruleum*

Table ST4. PBIQs **30** – **41** obtained by incubation of *X. caeruleum* plant material with hydroxylamines

Table ST5. ^{1}H NMR data of compounds **31** - **36**

Table ST6. ^{1}H NMR data of compounds **37** - **41**

Table ST7. ^{13}C NMR data of compounds **31** - **41**

Scheme SS1. Experimental HRESIMS and NMR spectra of **31**

Scheme SS2. Experimental HRESIMS and NMR spectra of **32**

Scheme SS3. Experimental HRESIMS and NMR spectra of **33**

Scheme SS4. Experimental HRESIMS and NMR spectra of **34**

Scheme SS5. Experimental HRESIMS and NMR spectra of **35**

Scheme SS6. Experimental HRESIMS and NMR spectra of **36**

Scheme SS7. Experimental HRESIMS and NMR spectra of **37**

Scheme SS8. Experimental HRESIMS and NMR spectra of **38**

Scheme SS10. Experimental HRESIMS and NMR spectra of **39**

Scheme SS11. Experimental HRESIMS and NMR spectra of **40**

Scheme SS12. Experimental HRESIMS and NMR spectra of **41**

Figure SF3. Test for antiproliferative effects of compounds **30** – **41** on HUVEC cells (72 h)

Figure SF4. Test for antiproliferative effects of compounds **30** – **41** on K-562 cells (72 h)

Figure SF5. Test for antiproliferative effects of compounds **30** – **41** on HELA cells (72 h)

Figure SF6. Enrichment of nucleosomes after induced apoptosis on K-562 by camptothecin and compounds **32** and **34** – **41**

Figure SF7. Apoptosis detection for PBIQs **30**-**41** by flow cytometry

Figure SF8. Apoptosis progression for compound **38**

Figure SF9. Microscopic images of K-562 cells, control

Figure SF10. Microscopic images of K-562 cells treated with **32**

Figure SF11. Microscopic images of K-562 cells treated with **34**

Figure SF12. Microscopic images of K-562 cells treated with **36**

Figure SF13. Microscopic images of K-562 cells treated with **37**

Figure SF14. Microscopic images of K-562 cells treated with **38**

Figure SF15. Microscopic images of K-562 cells treated with **39**

Figure SF16. Microscopic images of K-562 cells treated with **40**

Figure SF17. Microscopic images of K-562 cells treated with **41**

Table ST1. Chemical structures of previously reported PBIQs **4** -**29**.

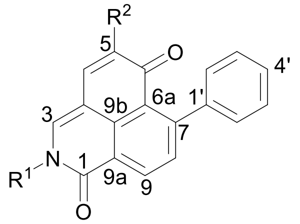

**Comp**	**R^1**	**R^2**	**Comp**	**R^1**	**R^2**	**Comp**	**R^1**	**R^2**
**4**	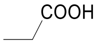	OH	**12**	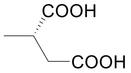	OH	**20**	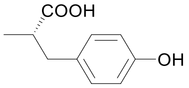	OH
**5**	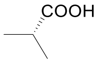	OH	**13**	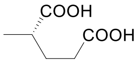	OH	**21**	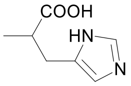	OH
**6**	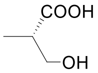	OH	**14**	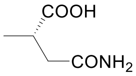	OH	**22**	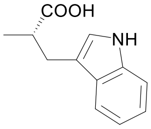	OH
**7**	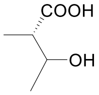	OH	**15**	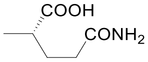	OH	**23**	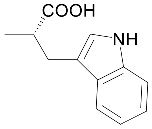	OCH_3
**8**	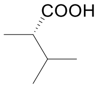	OH	**16**	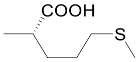	OH	**24**	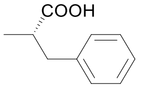	OCH_3
**9**	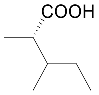	OH	**17**	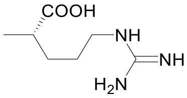	OH	**25**	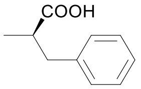	OCH_3
**10**	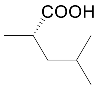	OH	**18**	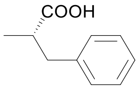	OH	**26**	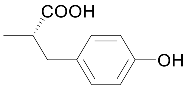	OCH_3
**11**	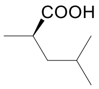	OH	**19**	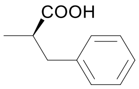	OH	**27**	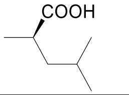	OCH_3

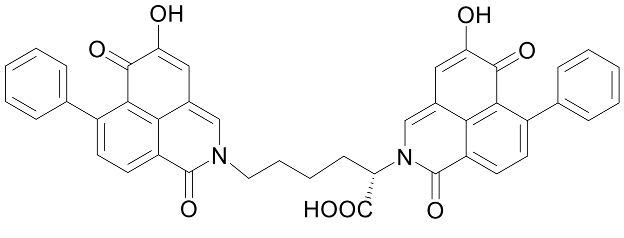

28

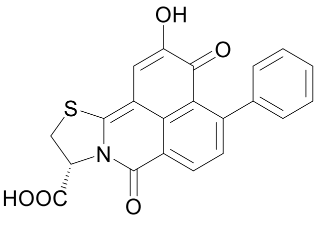

29

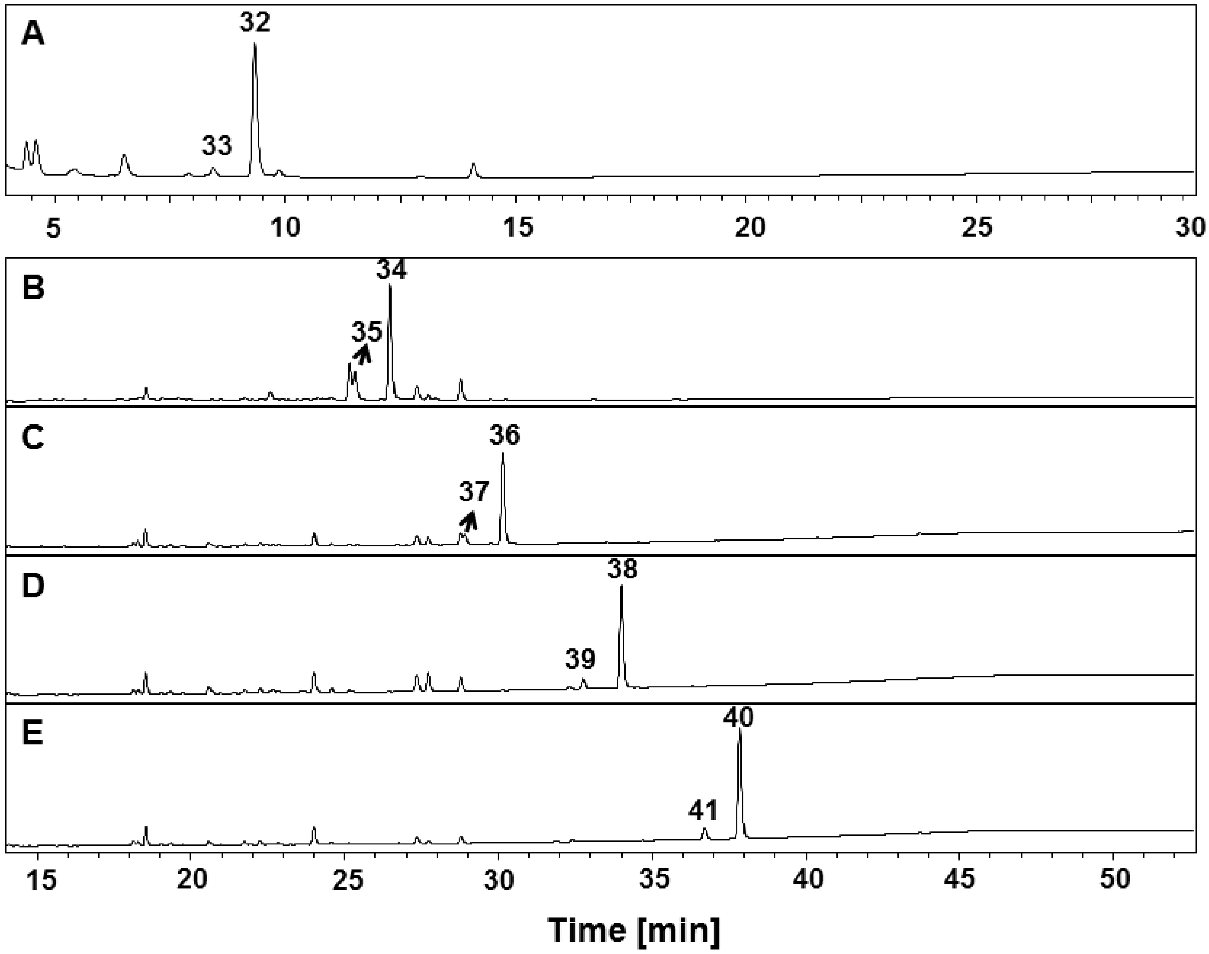

Figure SF1. HPLC-HRESIMS chromatograms (λ = 254 nm) of **32** to **41** after incubation of plant material with hydroxylamines (**A**, method B, gradient 2), 6-amino-1-hexanol (**B**, method B, gradient 1), 8-amino-1-octanol (**C**, method B, gradient 1), 10-amino-1-decanol (**D**, method B, gradient 1), 12-amino-1-dodecanol (**E**, method B, gradient 1).

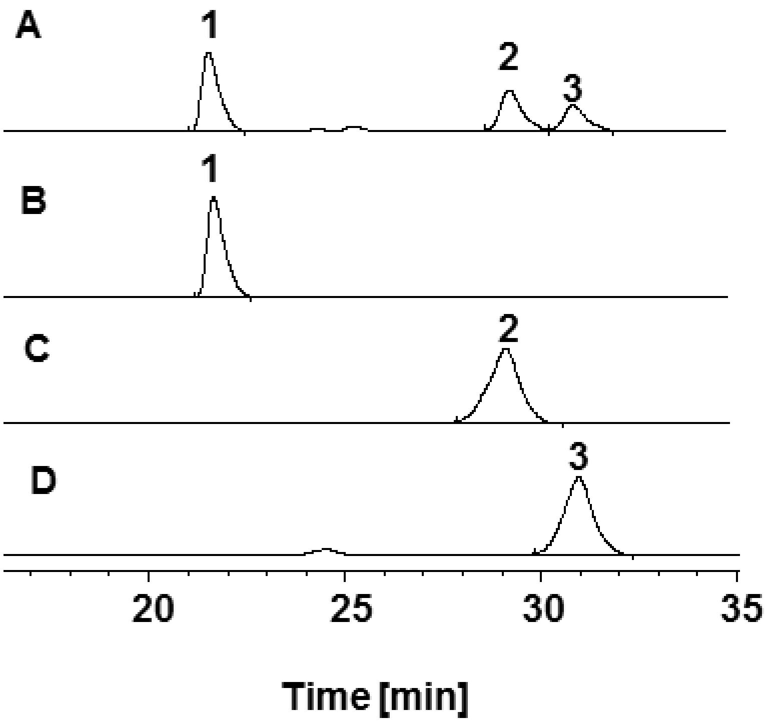

Figure SF2. HPLC-UV chromatograms (λ = 254 nm) of samples of raw plant material (A) and standard compounds **1** – **3** (B-D) .

Table ST2. Calibration curves and test ranges of standard compounds **1** – **3**

Analytes	Calibration curves[a]	r^2	Test ranges (µg / ml)	LOD (µg / ml)	LOQ (µg / ml)
**1**	$y = 29.10x - 18.20$	1.0000	9.69-1240.36	1.27	5.09
**2**	$y = 26.08x + 0.94$	1.0000	30.94-495.00	2.85	8.55
**3**	$y = 25.37x - 64.80$	1.0000	30.00-480.00	2.24	6.30

[a] *y*, Peak area count; *x*, concentration of standard (µg/ml).

Table ST3. Concentration of compounds **1** – **3** in above ground plant material of *X. caeruleum* (samples A – C)

Sample / comp.	*Xiphidium caeruleum*			
	A	B	C	Mean ± SD
**1** (mg g^{-1})	0.62	0.69	0.80	0.70 ± 0.09
**2** (mg g^{-1})	0.32	0.30	0.31	0.31 ± 0.01
**3** (mg g^{-1})	0.33	0.34	0.34	0.33 ± 0.01

Table ST4. PBIQs **30** – **41** obtained by incubation of *X. caeruleum* plant material with hydroxylamines

Plant material	ω-Hydroxy-*n*-alkylamines	Products	Yield
[g]	Name, mass [mg]	No., mass [mg]	%
100 g	Ethanolamine, 100 mg	**30**, 15.08 mg	23
		**31**, 2.97 mg	14
60 g	Butanolamine, 60 mg	**32**, 7.21 mg	17
		**33**, 2.09 mg	16
80 g	6-Amino-1-hexanol, 100 mg	**34**, 17.27 mg	28
		**35**, 4.94 mg	26
112 g	8-Amino-1-octanol, 124 mg	**36**, 17.36 mg	19
		**37**, 4.90 mg	17
110 g	10-Amino-1-decanol, 125 mg	**38**, 19.38 mg	20
		**39**, 3.65 mg	12
119 g	12-Amino-1-dodecanol, 148 mg	**40**, 19.34 mg	17
		**41**, 3.97 mg	12

Table ST5. ^{1}H NMR data of compounds **31** to **36** (500 MHz, δ values, J in Hz)[a]

Position	**31**	**32**	**33**	**34**	**35**	**36**
	δ_H (mult., J)	δ_H (mult., J)	δ_H (mult., J)	δ_H (mult., J)	δ_H (mult., J)	δ_H (mult., J)
3	7.96 (s)	8.09 (s)	8.03 (s)	8.09 (s)	8.01 (s)	7.53 (s)
4	6.99 (s)	7.01 (s)	6.98 (s)	7.00 (s)	6.96 (s)	6.91 (s)
8	7.52 (d, 8.1)	7.58 (d, 8.1)	7.54 (d, 8.1)	7.58 (d, 8.1)	7.54 (d, 8.1)	7.58 (d, 8.1)
9	8.60 (d, 8.1)	8.67 (d, 8.1)	8.61 (d, 8.1)	8.67 (d, 8.1)	8.61 (d, 8.1)	8.74 (d, 8.1)
2'/6'	7.30 (dd, 7.9, 1.7)	7.34 - 7.36 (m)	7.30 - 7.34 (m)	7.34 - 7.36 (m)	7.30 - 7.32 (m)	7.31 - 7.33 (m)
3'/4'/5'	7.35 - 7.41 (m)	7.38 - 7.45 (m)	7.34 - 7.41 (m)	7.39 - 7.45 (m)	7.36 - 7.41 (m)	7.44 - 7.49 (m)
1''	4.21 (t, 5.0)	4.18 (t, 7.3)	4.17 (t, 7.3)	4.14 (t, 7.3)	4.12 (t, 7.3)	4.07 (t, 7.4)
2''	3.93 (t, 5.2)	1.91 – 1.97 (m)	1.91 – 1.95 (m)	1.84 – 1.90 (m)	1.85 – 1.88 (m)	1.81 – 1.86 (m)
3''		1.60 – 1.66 (m)	1.60 – 1.65 (m)	1.44 - 1.47 (m)	1.44 - 1.47 (m)	1.30 - 1.41 (m)
4''		3.62 (dd,11.5, 6.1)	3.62 (dd,11.4, 6.1)	1.44 - 1.47 (m)	1.44 - 1.47 (m)	1.30 - 1.41 (m)
5''				1.51 – 1.55 (m)	1.52 – 1.55 (m)	1.30 - 1.41 (m)
6''				3.52 – 3.56 (m)	3.52 – 3.55 (m)	1.30 - 1.41 (m)
7''						1.51 – 1.57 (m)
8''						3.62 (t, 6.5)
5-OCH_3	3.77 (s)		3.76 (s)		3.76 (s)	

[a] Compounds **31** - **35** were measured in acetone-d_6, and compound **36** was measured in chloroform-d.

Table ST6. ^{1}H NMR data of compounds **37** to **41** (500 MHz, δ values, J in Hz) [a]

Position	**37**	**38**	**39**	**40**	**41**
	δ_H (mult., J)	δ_H (mult., J)	δ_H (mult., J)	δ_H (mult., J)	δ_H (mult., J)
3	7.46 (s)	8.08 (s)	8.02 (s)	7.51 (s)	7.45 (s)
4	6.61 (s)	7.00 (s)	6.97 (s)	6.88 (s)	6.60 (s)
8	7.55 (d, 8.1)	7.58 (d, 8.0)	7.55 (d, 8.1)	7.57 (d, 8.1)	7.54 (d, 8.1)
9	8.67 (d, 8.1)	8.67 (d, 8.0)	8.62 (d, 8.1)	8.73 (d, 8.1)	8.67 (d, 8.1)
2'/6'	7.29 - 7.31 (m)	7.34 - 7.36 (m)	7.30 - 7.32 (m)	7.30 - 7.33 (m)	7.27 - 7.30 (m)
3'/4'/5'	7.34 - 7.41 (m)	7.38 - 7.45 (m)	7.34 - 7.41 (m)	7.42 - 7.48 (m)	7.33 - 7.40 (m)
1''	4.05 (t, 7.3)	4.14 (t, 7.3)	4.13 (t, 7.3)	4.06 (t, 7.4)	4.05 (t, 7.3)
2''	1.78 – 1.85 (m)	1.83 – 1.89 (m)	1.83 – 1.89 (m)	1.80 - 1.86 (m)	1.79 – 1.85 (m)
3''	1.30 - 1.42 (m)	1.27 - 1.44 (m)	1.27 - 1.44 (m)	1.24 - 1.43 (m	1.23 - 1.42 (m)
4''	1.30 - 1.42 (m)	1.27 - 1.44 (m)	1.27 - 1.44 (m)	1.24 - 1.43 (m	1.23 - 1.42 (m)
5''	1.30 - 1.42 (m)	1.27 - 1.44 (m)	1.27 - 1.44 (m)	1.24 - 1.43 (m)	1.23 - 1.42 (m)
6''	1.30 - 1.42 (m)	1.27 - 1.44 (m)	1.27 - 1.44 (m)	1.24 - 1.43 (m)	1.23 - 1.42 (m)
7''	1.51 – 1.56 (m)	1.27 - 1.44 (m)	1.27 - 1.44 (m)	1.24 - 1.43 (m)	1.23 - 1.42 (m)
8''	3.62 (t, 6.6)	1.27 - 1.44 (m)	1.27 - 1.44 (m)	1.24 - 1.43 (m)	1.23 - 1.42 (m)
9''		1.46 – 1.51 (m)	1.45 – 1.51 (m)	1.24 - 1.43 (m)	1.23 - 1.42 (m)
10''		3.51 (t, 6.6)	3.51 (t, 5.7)	1.24 - 1.43 (m)	1.23 - 1.42 (m)
11''				1.51 – 1.57 (m)	1.50 – 1.56 (m)
12''				3.62 (t, 6.7)	3.61 (t, 6.7)
5-OCH_3	3.80 (s)		3.76 (s)		3.79 (s)

[a] Compounds **37**, **40** and **41** were measured in chloroform-*d*, and compounds **38** and **39** were measured in acetone-d_6.

Table ST7. ^{13}C NMR data of compounds **31** to **41** (125 MHz, δ values) [a]

Position	**31**	**32**	**33**	**34**	**35**	**36**	**37**	**38**	**39**	**40**	**41**
1	161.8	161.7	162.3	161.7	161.5	161.5	161.4	161.7	161.3	161.5	161.4
3	138.8	138.6	137.3	138.7	137.3	136.6	134.9	138.6	137.1	136.5	134.9
3a	109.4	110.1	109.4	110.2	110.1	110.0	110.1	110.1	109.9	110.1	110.1
4	110.4	110.8	110.4	110.8	110.2	109.9	108.3	110.8	110.0	109.7	108.5
5	152.5	149.3	153.1	149.3	152.5	148.2	151.9	149.3	152.3	148.2	152.0
6	178.2	179.1	178.2	179.2	178.2	178.8	178.5	179.1	178.0	178.8	178.5
6a	125.3	125.0	126.8	125.0	125.4	123.7	124.4	125.0	125.3	123.8	124.5
7	150.6	150.9	151.0	150.9	150.5	150.9	150.8	150.9	150.3	150.9	150.8
8	132.3	132.3	132.5	132.4	132.5	131.9	132.3	132.3	132.3	131.9	132.3
9	132.6	133.4	132.6	133.4	132.7	133.6	132.7	133.4	132.5	133.6	132.7
9a	127.0	125.6	127.8	125.5	127.1	124.7	125.8	125.6	127.6	124.8	126.0
9b	133.1	133.0	133.4	133.0	132.9	132.2	132.1	133.0	132.9	132.2	132.1
1'	143.6	143.1	144.3	143.1	143.6	141.6	141.9	143.1	143.4	141.6	142.0
2'/6'	129.1	129.2	129.2	129.2	129.1	128.2	128.2	129.2	129.3	128.2	128.1
3'/5'	128.6	128.6	128.6	128.6	128.6	128.1	128.1	128.6	128.9	128.1	128.1
4'	127.8	128.1	127.8	128.1	127.9	128.0	127.5	128.1	128.0	128.0	127.5
1''	53.0	49.9	49.8	50.0	49.9	50.2	50.0	50.1	49.8	50.2	50.1
2''	60.6	26.8	26.8	29.8	29.8	29.4	29.4	29.9	29.7	29.4	29.4
3''		30.7	30.6	27.2	27.2	26.8	26.8	27.4	27.4	26.9	26.9
4''		62.1	62.1	26.4	26.4	29.4	29.4	30.4	30.2	29.5 -29.7	29.4 -29.7
5''				33.6	33.6	29.5	29.5	30.4	30.2	29.5 -29.7	29.4 -29.7
6''				62.3	62.3	25.8	25.8	30.4	30.2	29.5 -29.7	29.4 -29.7
7''						32.8	32.8	30.4	30.2	29.5 -29.7	29.4 -29.7
8''						63.2	63.1	26.8	26.7	29.5 -29.7	29.4 -29.7
9''								33.9	33.5	29.5 -29.7	29.4 -29.7
10''								62.6	62.3	25.9	25.9
11''										33.0	33.0
12''										63.2	63.2
5-OCH_3	55.8		55.9		55.8		55.8		55.7		55.8

[a] Compounds **31** – **35**, **38**, and **39** were measured in acetone-d_6, and compounds **36**, **37**, **40** and **41** were measured in chloroform-*d*

Scheme SS1. Experimental HRESIMS and NMR spectra of **31**

2-(2''-Hydroxyethyl)-5-methoxy-7-phenyl-2H-benzo[de]isoquinoline-1,6-dione (31)

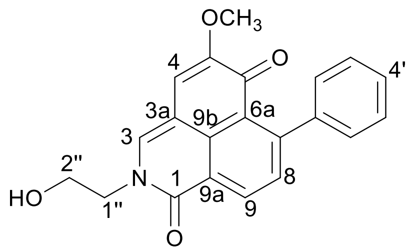

Structure of **31**

HPLC-PDA-HRESIMS (method B, gradient 2) chromatogram of **31** (UV 254 nm)

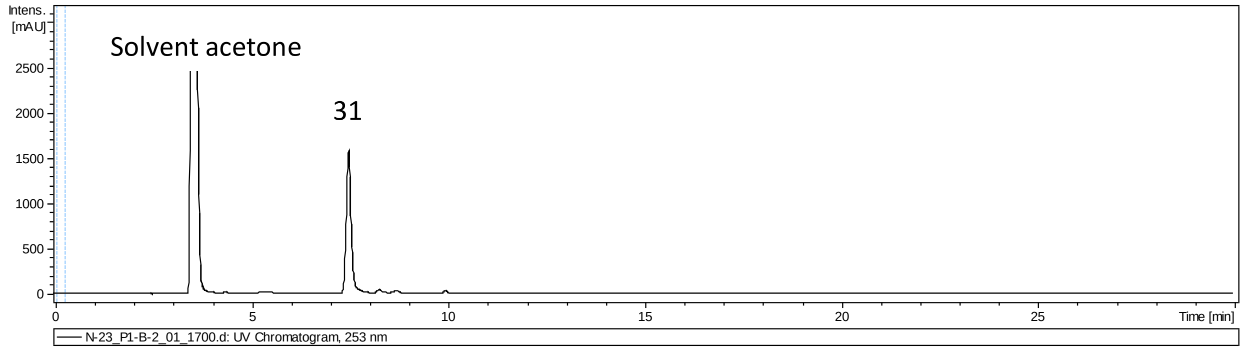

UV and HRESIMS spectrum of **31**

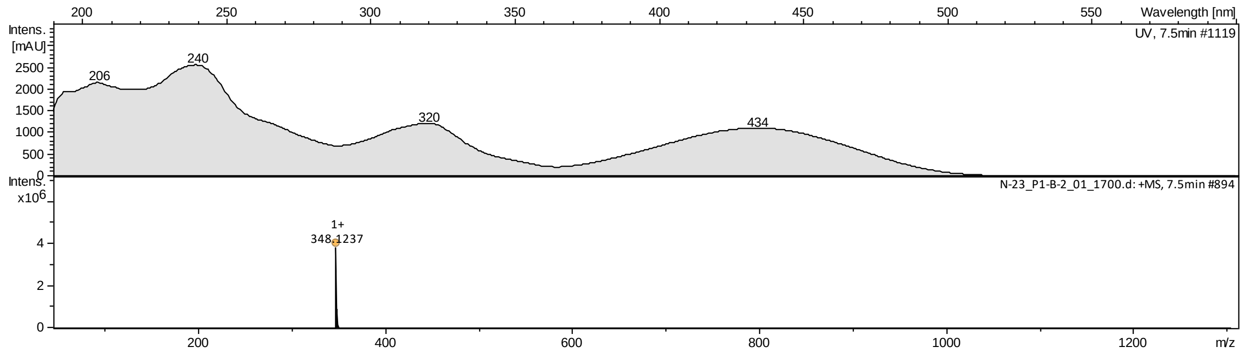

^{1}H NMR spectrum (500 MHz, acetone-d_6) of **31**

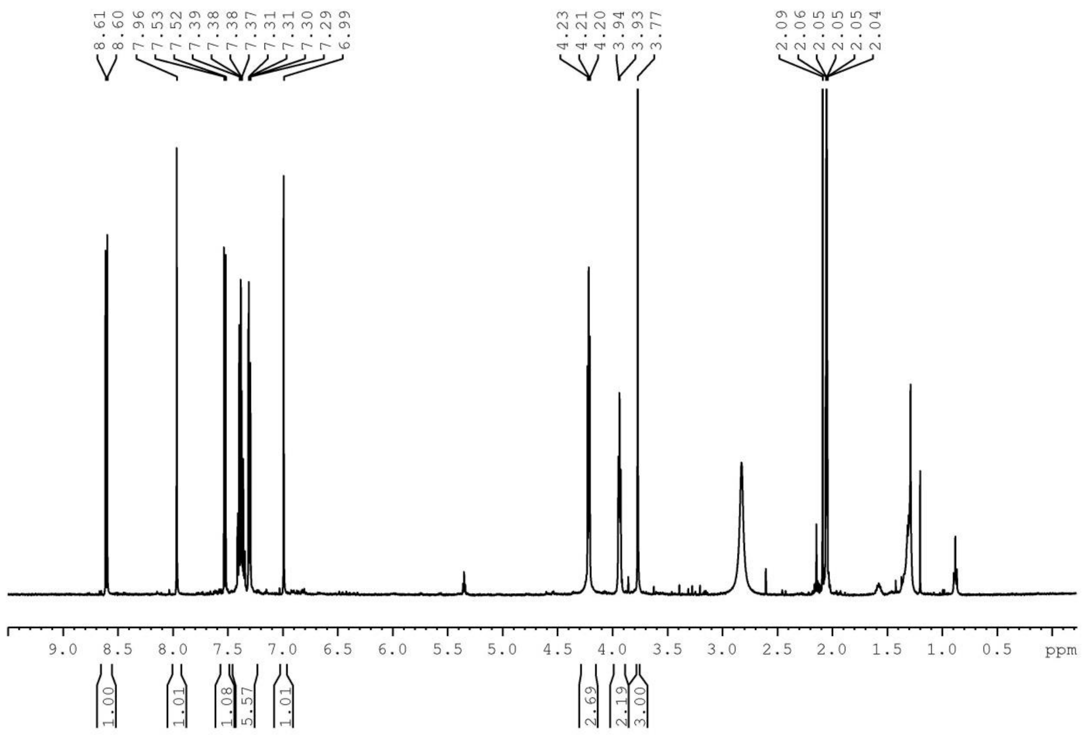

^{13}C NMR spectrum (125 MHz, acetone-d_6) of of **31**

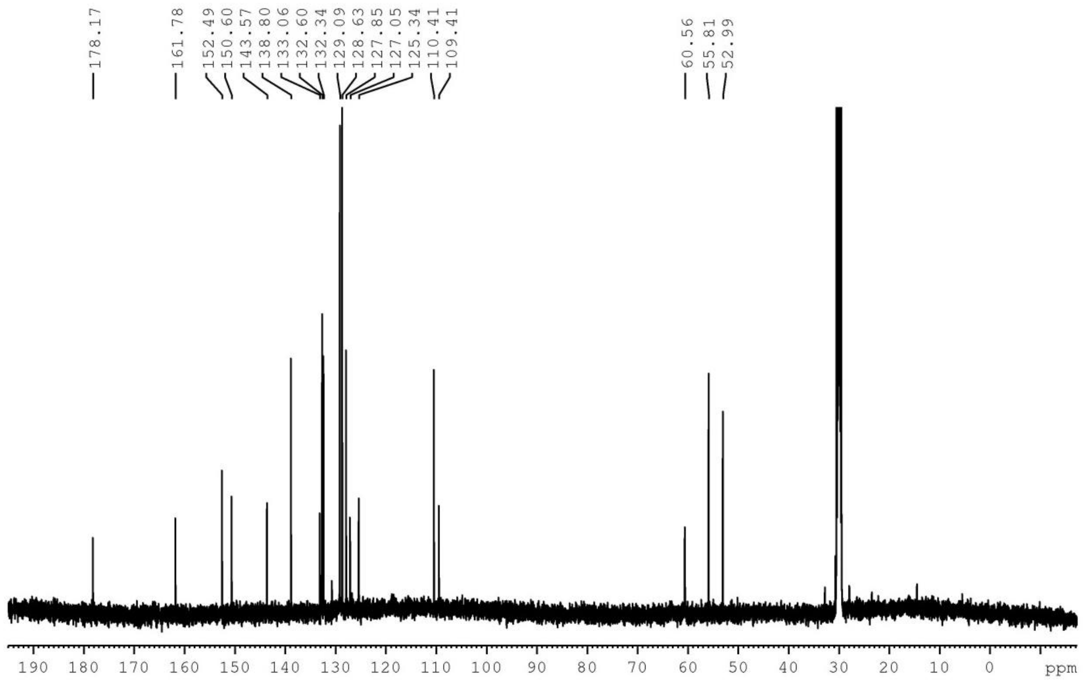

HSQC spectrum (500 MHz, acetone-d_6) of **31**

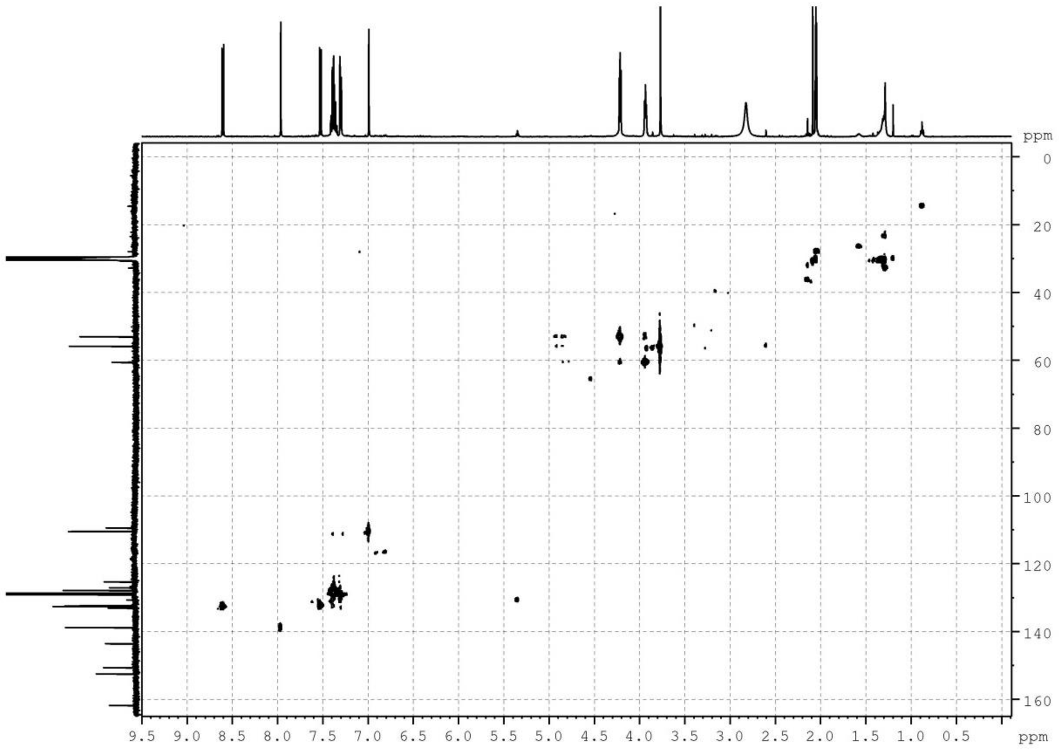

HMBC spectrum (500 MHz, acetone-d_6) of **31**

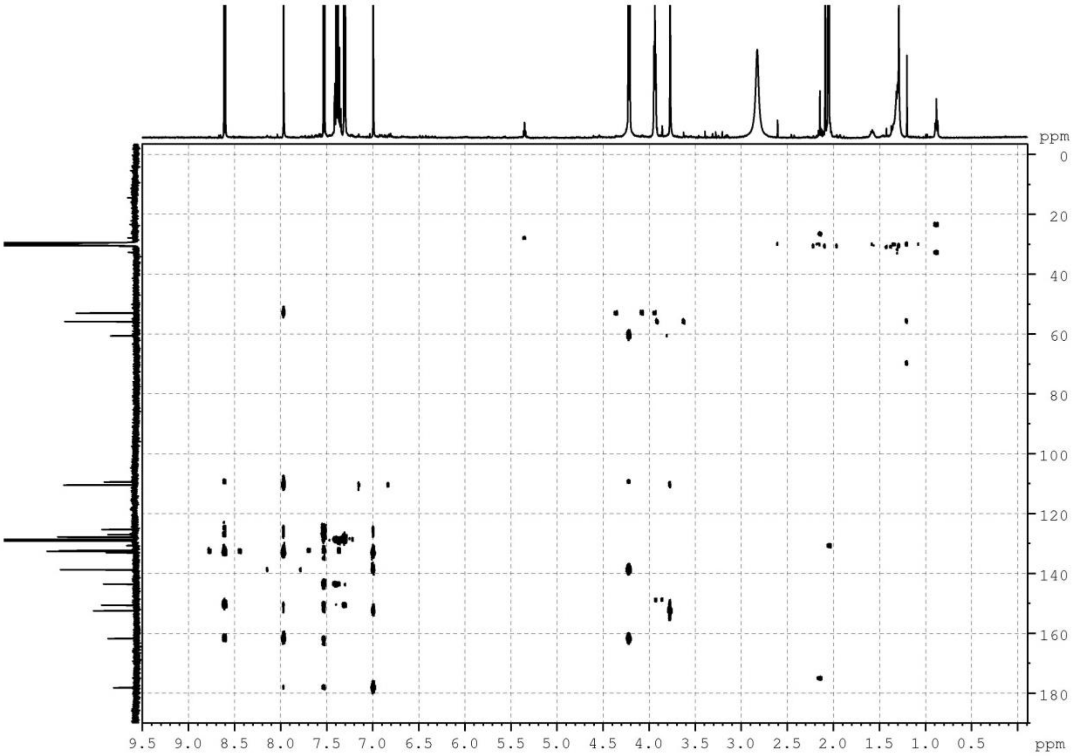

Scheme SS2. Experimental HRESIMS and NMR spectra of **32**

2-(4''-Hydroxybutyl)-5-hydroxy-7-phenyl-2H-benzo[de]isoquinoline-1,6-dione (32)

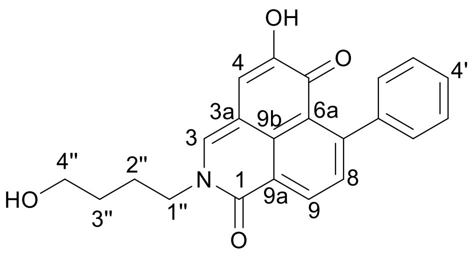

Structure of **32**

HPLC-PDA-HRESIMS (method B, gradient 2) chromatogram of **32** (UV 254 nm)

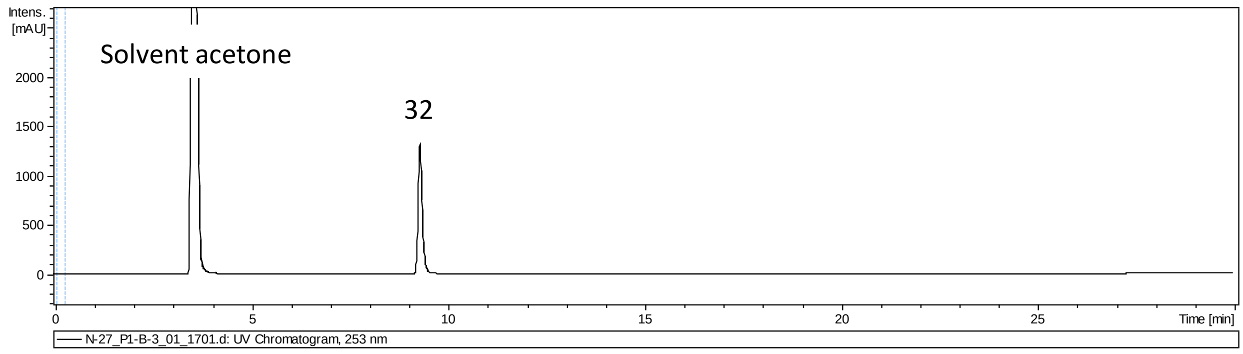

UV and HRESIMS spectrum of **32**

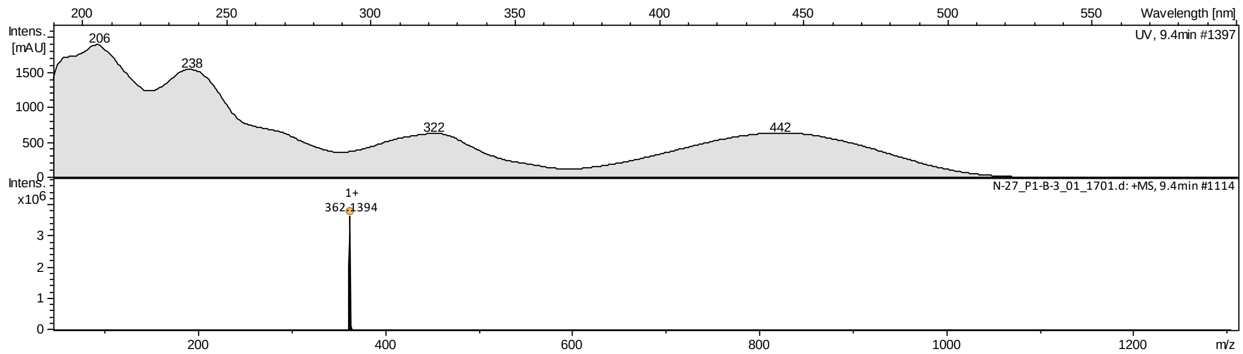

^{1}H NMR spectrum (500 MHz, acetone-d_6) of **32**

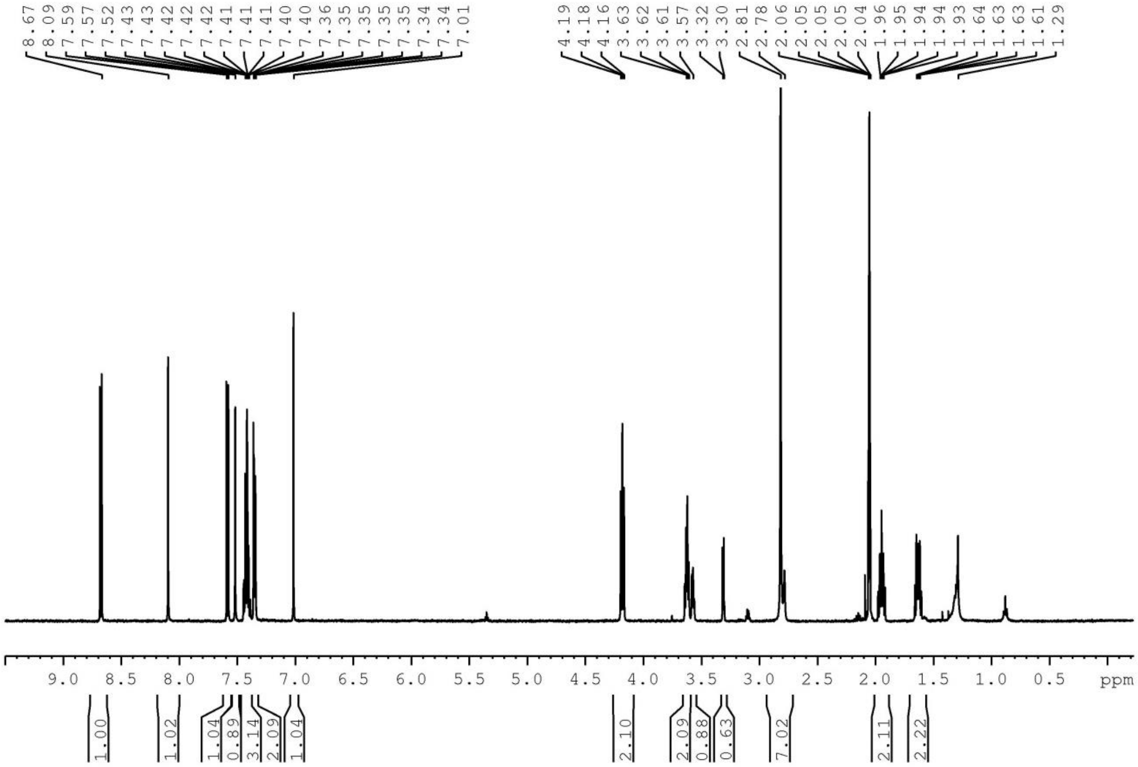

^{13}C NMR spectrum (125 MHz, acetone-d_6) of **32**

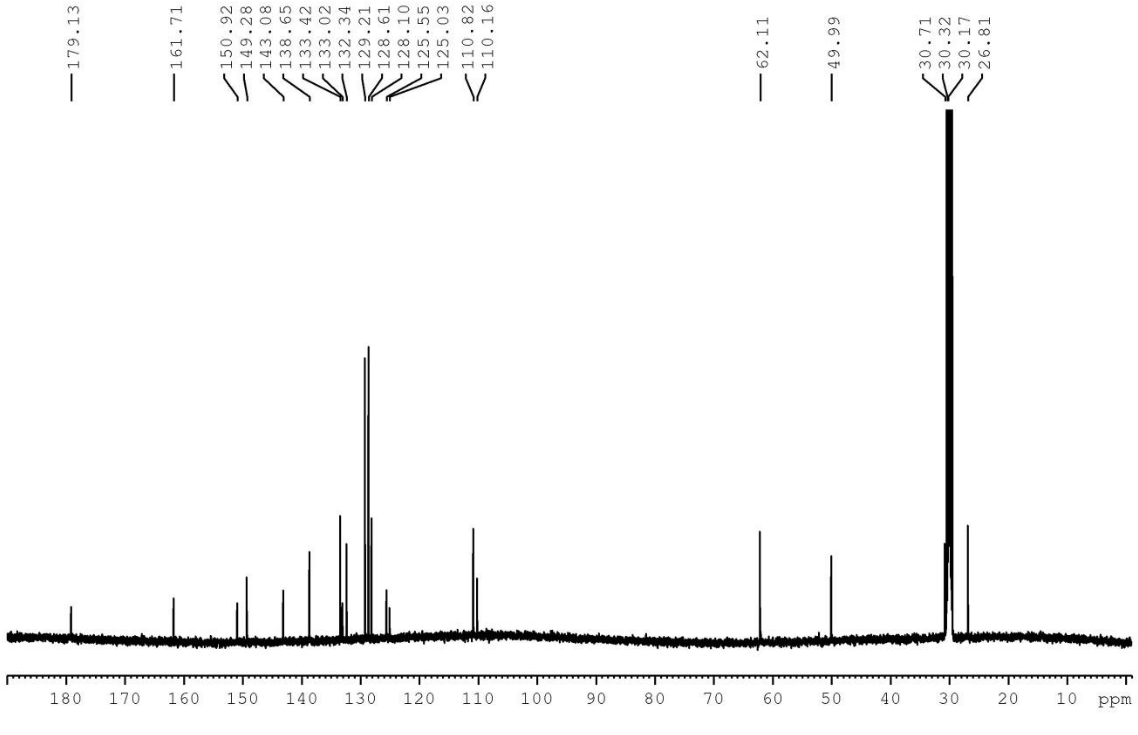

HSQC spectrum (500 MHz, acetone-d_6) of **32**

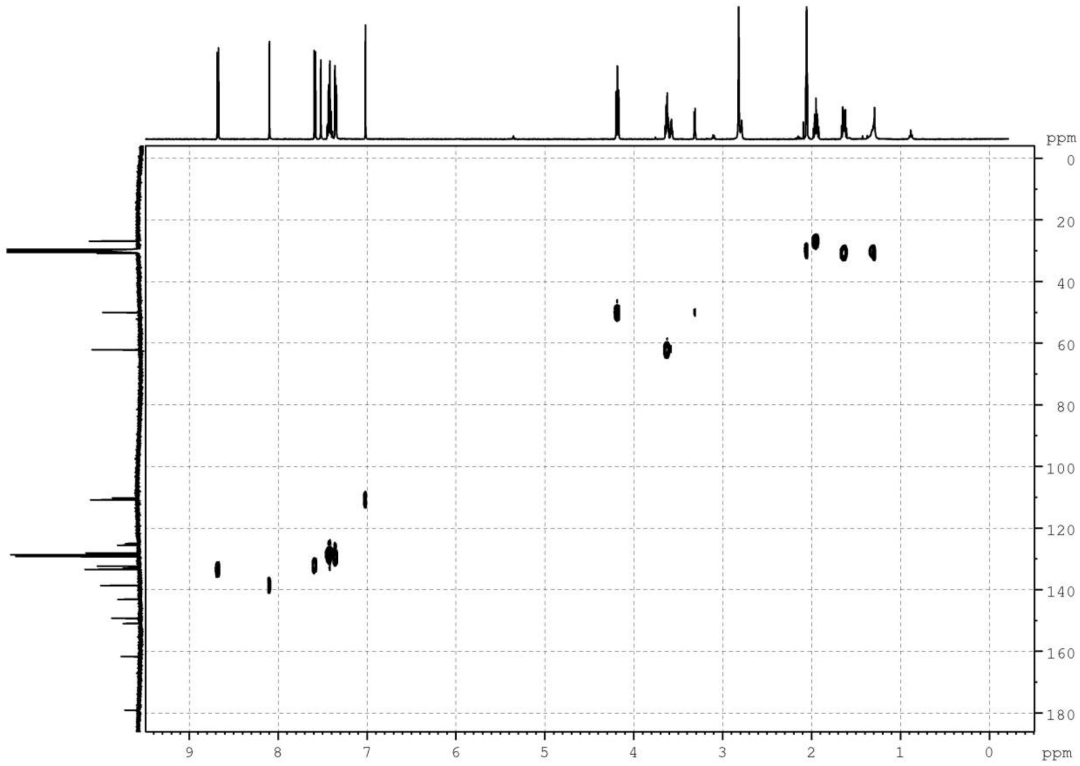

HMBC spectrum (500 MHz, acetone-d_6) of **32**

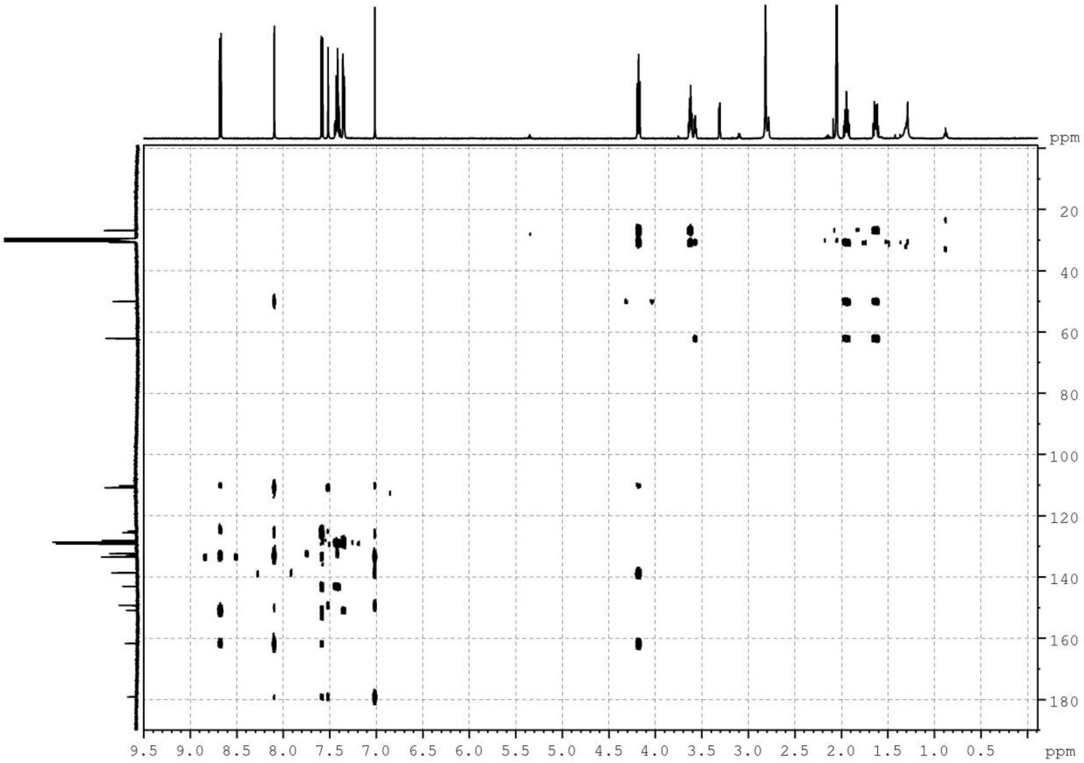

Scheme SS3. Experimental HRESIMS and NMR spectra of **33**

2-(4''-Hydroxybutyl)-5-methoxy-7-phenyl-2H-benzo[de]isoquinoline-1,6-dione (33)

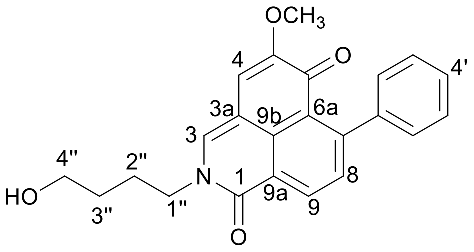

Structure of **33**

HPLC-PDA-HRESIMS (method B, gradient 2) chromatogram of **33** (UV 254 nm)

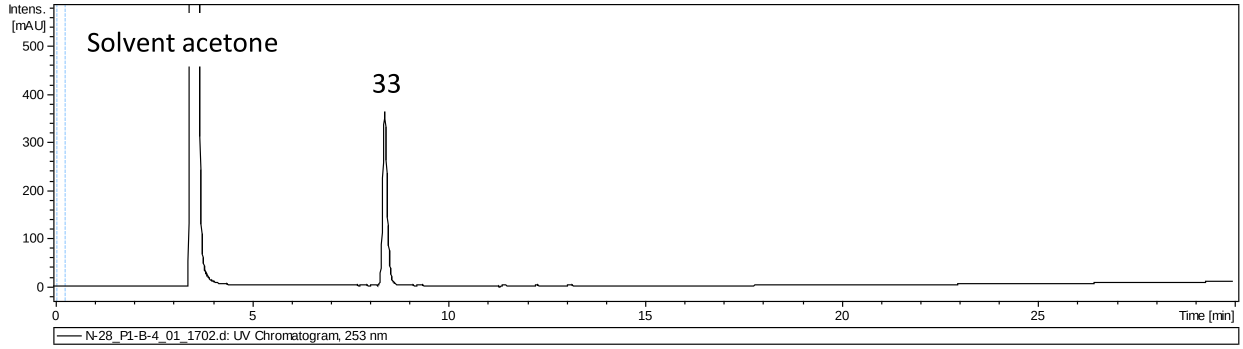

UV and HRESIMS spectrum of **33**

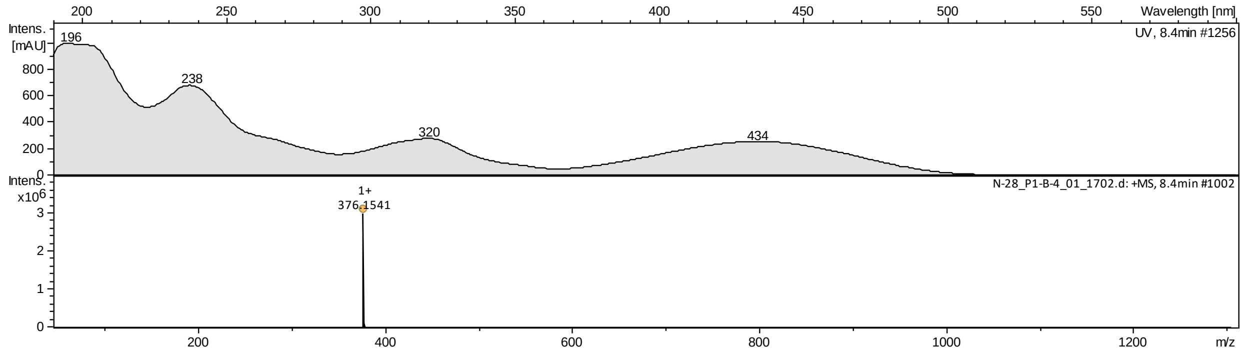

^{1}H NMR spectrum (500 MHz, acetone-d_6) of **33**

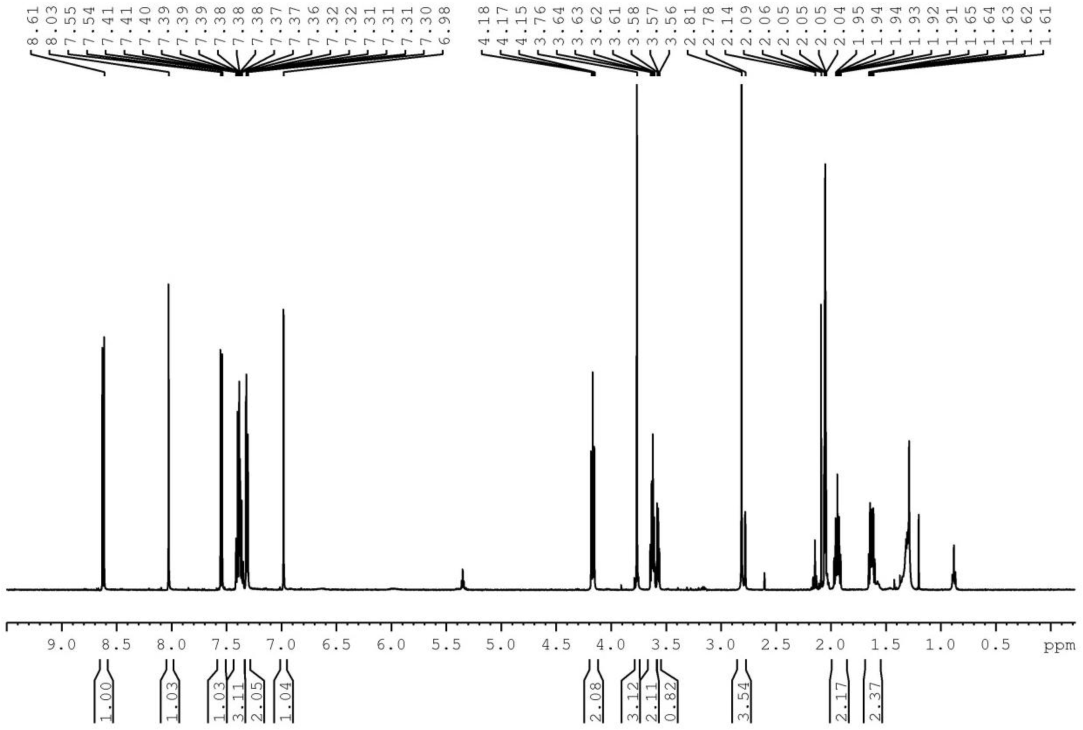

HSQC spectrum (500 MHz, acetone-d_6) of **33**

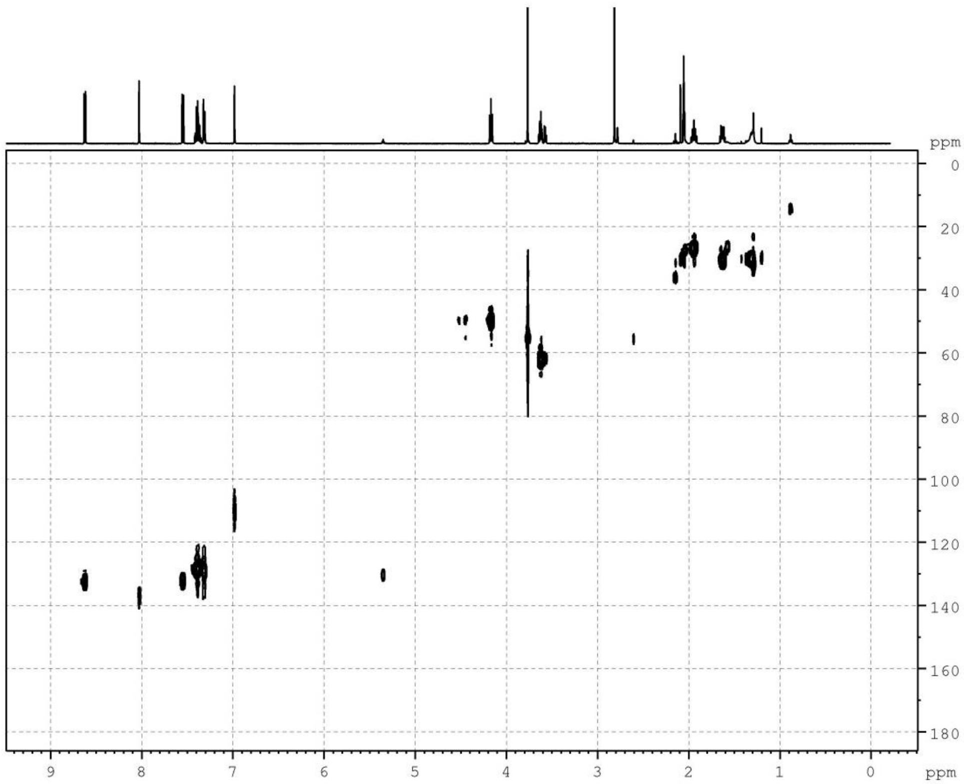

HMBC spectrum (500 MHz, acetone-d_6) of **33**

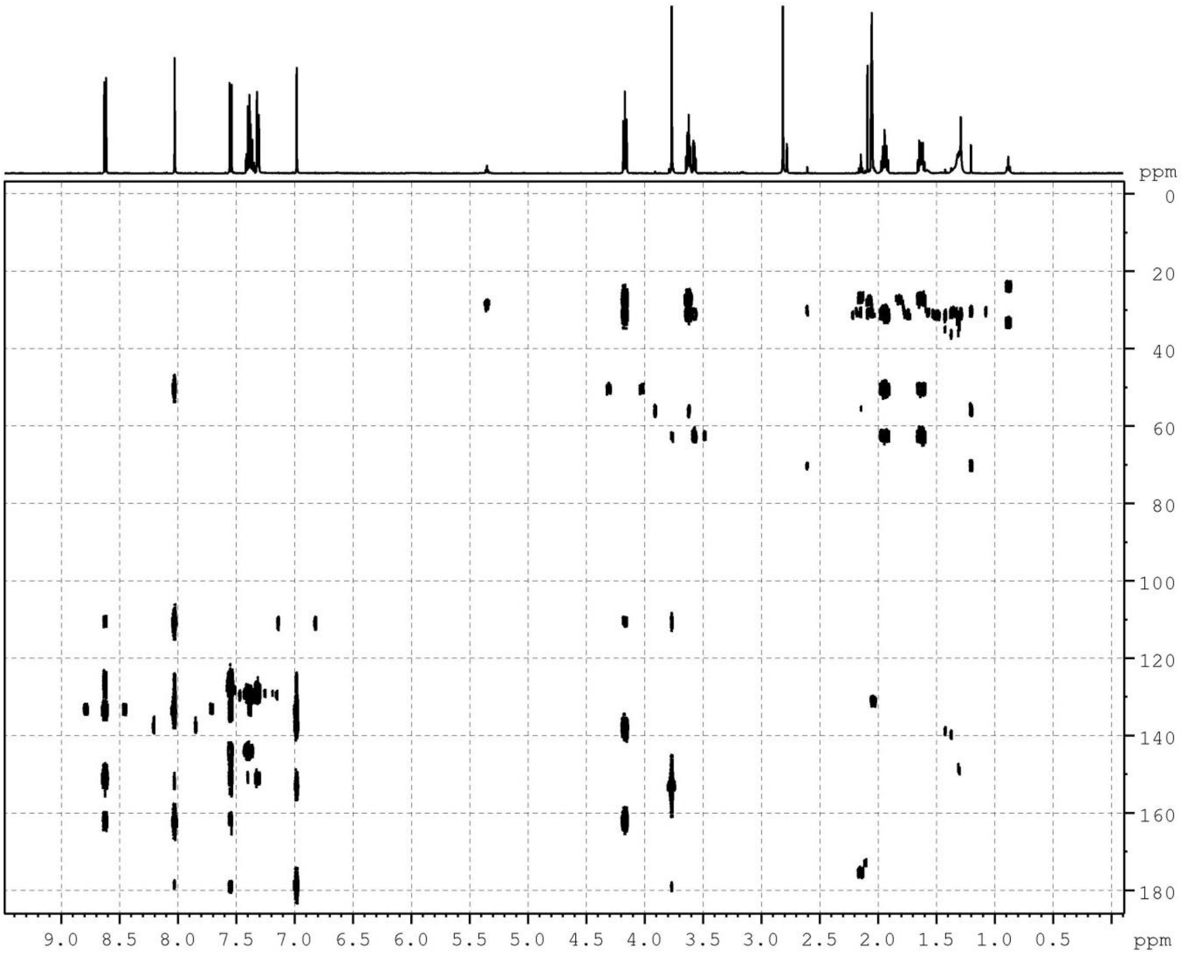

Scheme SS4. Experimental HRESIMS and NMR spectra of **34**

2-(6''-Hydroxyhexyl)-5-hydroxy-7-phenyl-2H-benzo[de]isoquinoline-1,6-dione (34)

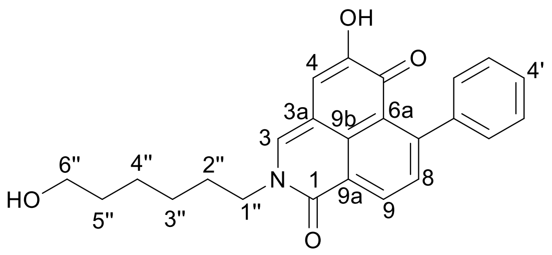

Structure of **34**

HPLC-PDA-HRESIMS (method B, gradient 2) chromatogram of **34** (UV 254 nm)

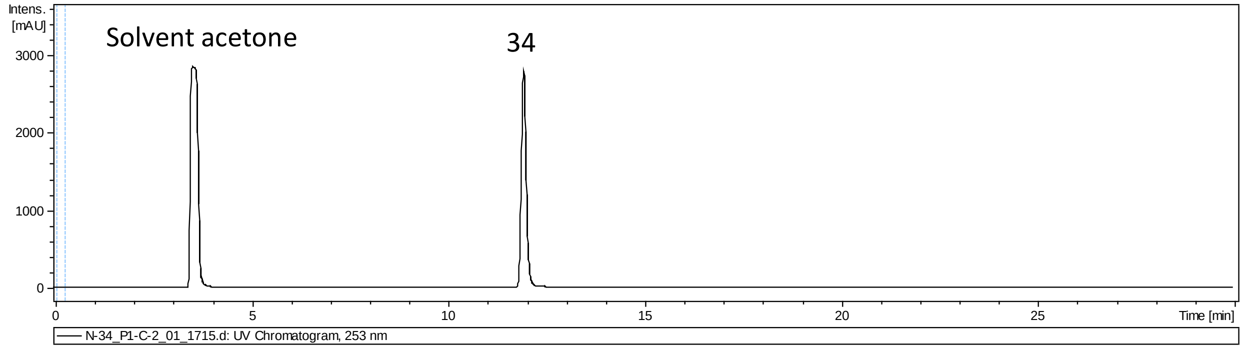

UV and HRESIMS spectrum of **34**

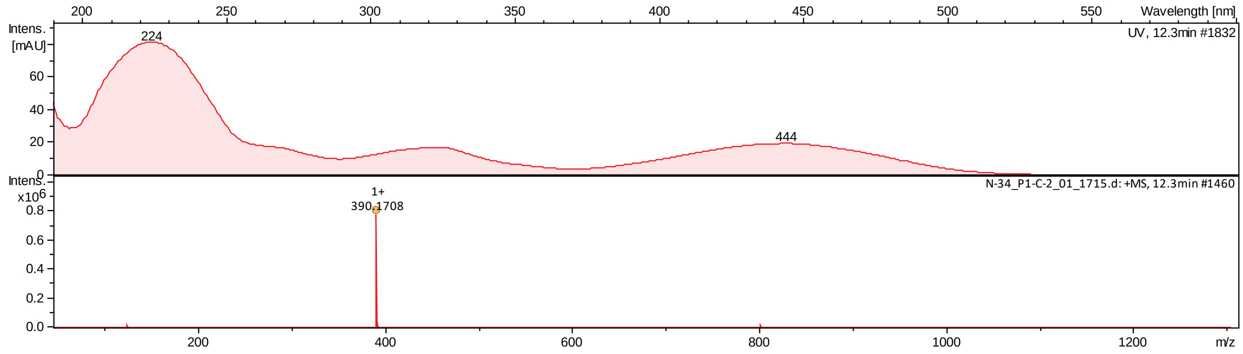

^{1}H NMR spectrum (500 MHz, acetone-d_6) of **34**

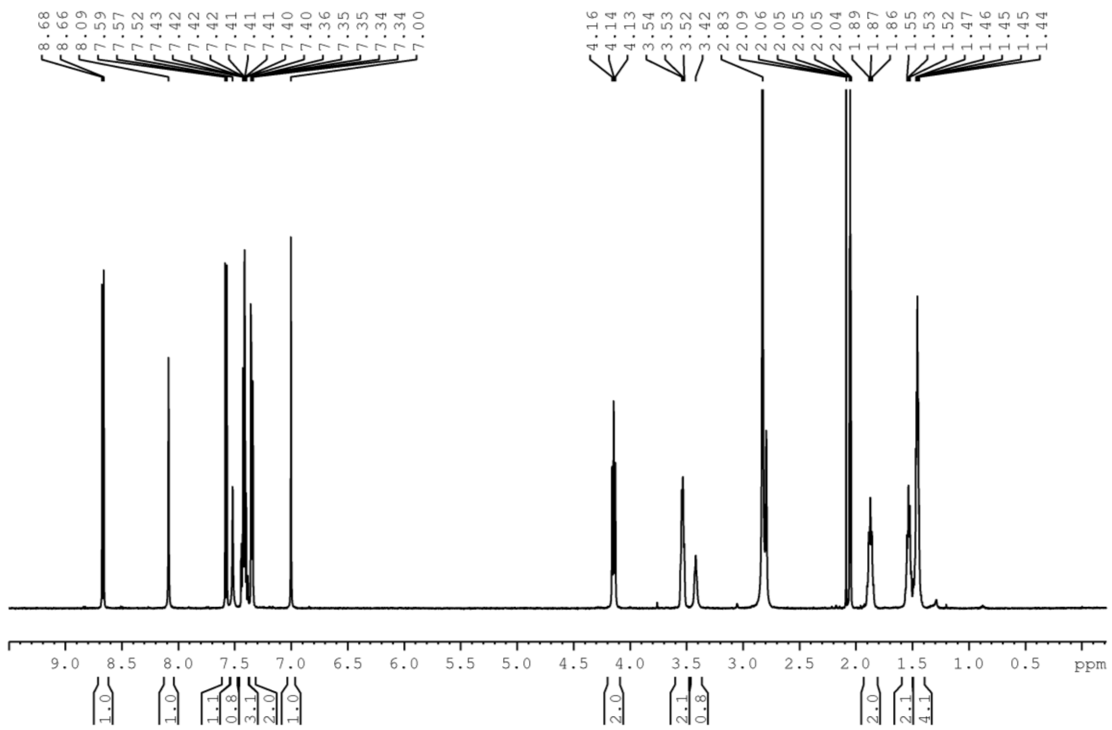

^{13}C NMR spectrum (125 MHz, acetone-d_6) of **34**

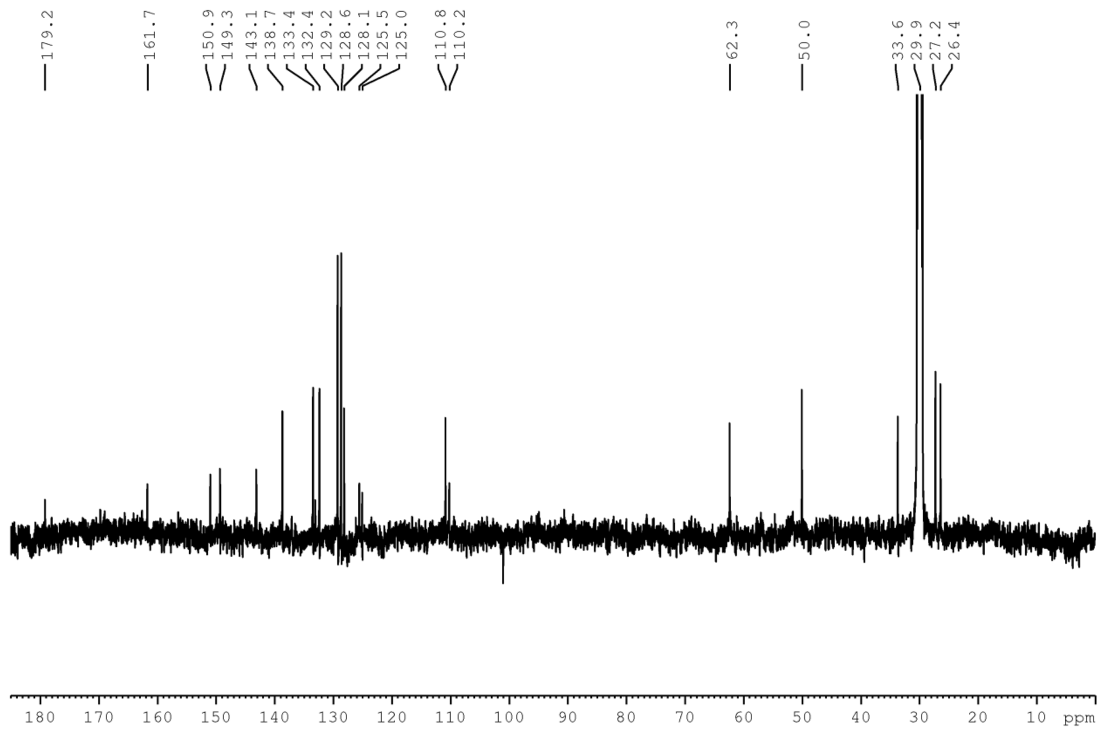

COSY spectrum (500 MHz, acetone-d_6) of **34**

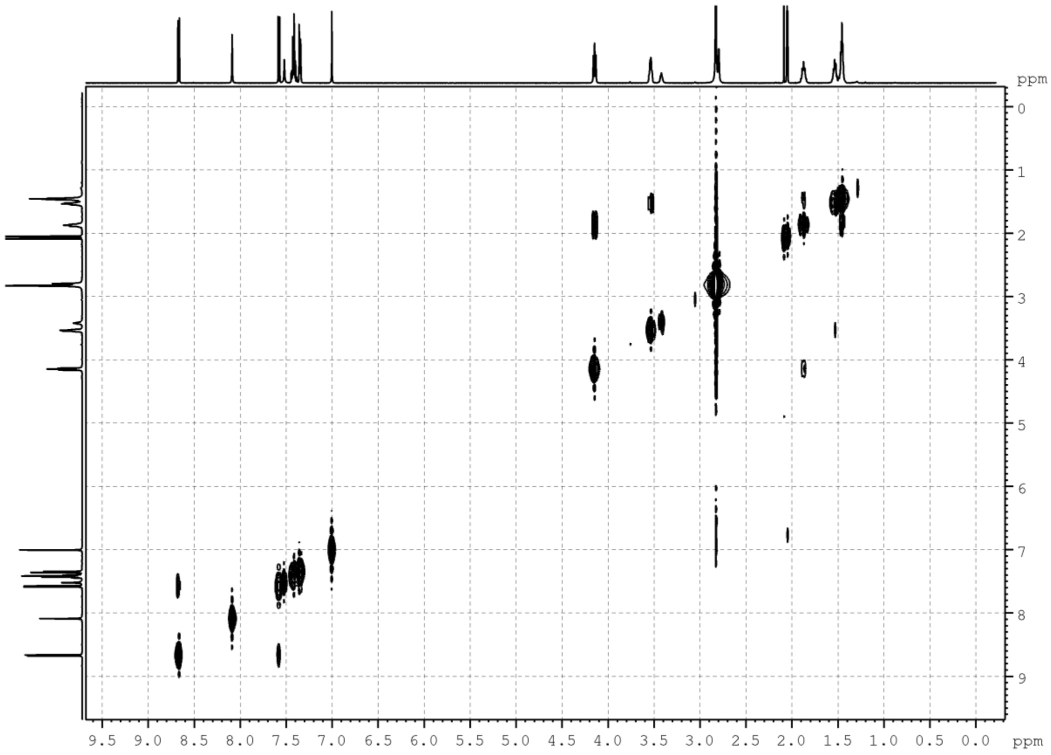

HSQC spectrum (500 MHz, acetone-d_6) of **34**

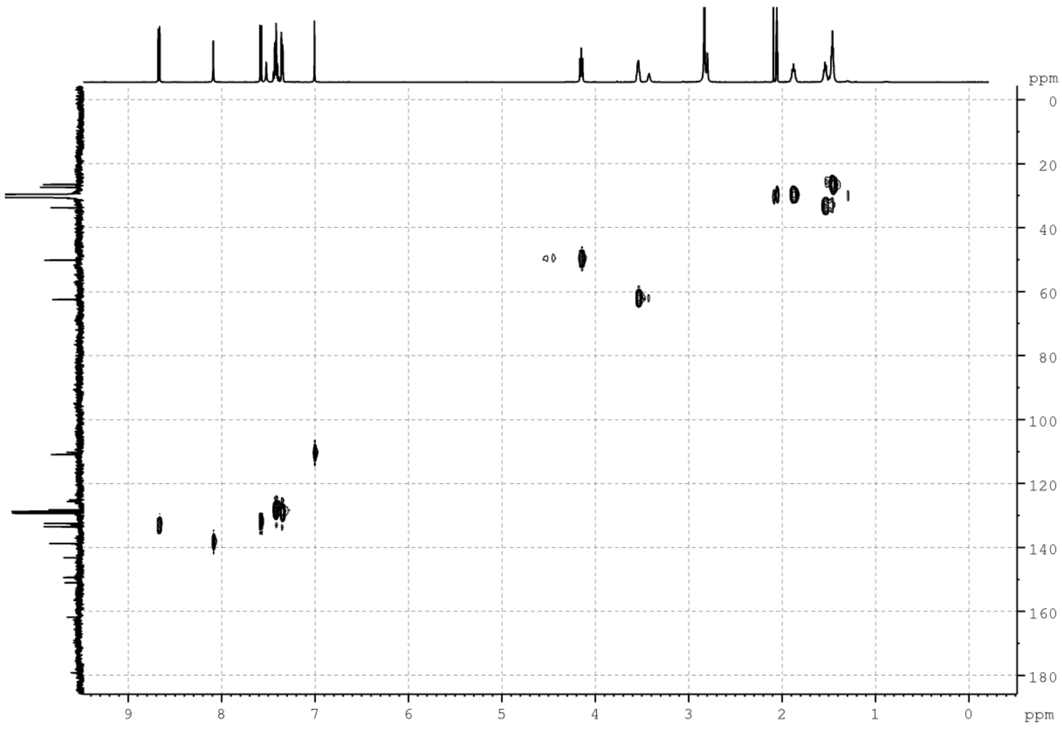

HMBC spectrum (500 MHz, acetone-d_6) of **34**

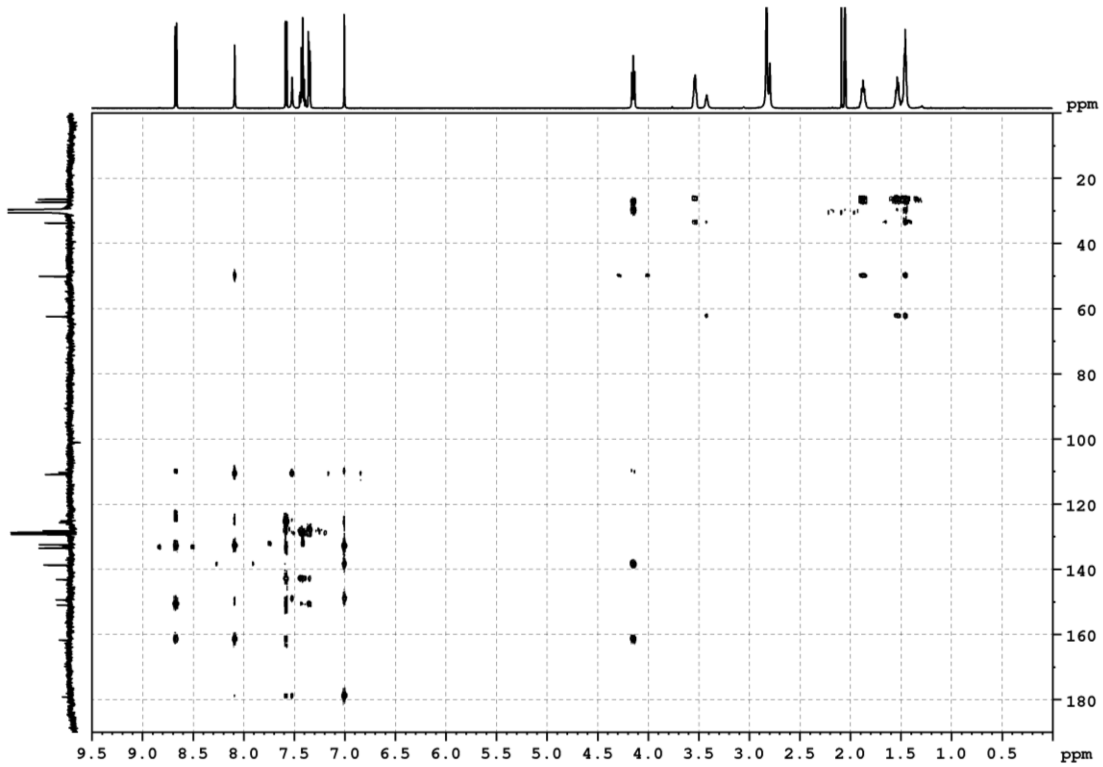

Scheme SS5. Experimental HRESIMS and NMR spectra of **35**

2-(6''-Hydroxyhexyl)-5-methoxy-7-phenyl-2H-benzo[de]isoquinoline-1,6-dione (35)

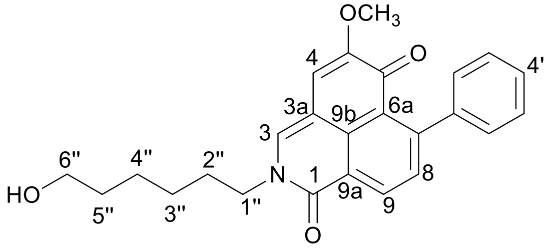

Structure of **35**

HPLC-PDA-HRESIMS (method B, gradient 2) chromatogram of **35** (UV 254 nm)

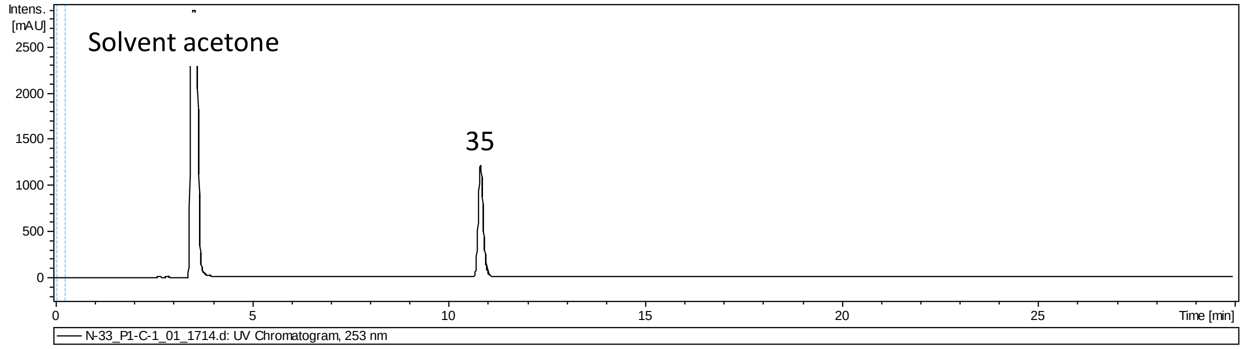

UV and HRESIMS spectrum of **35**

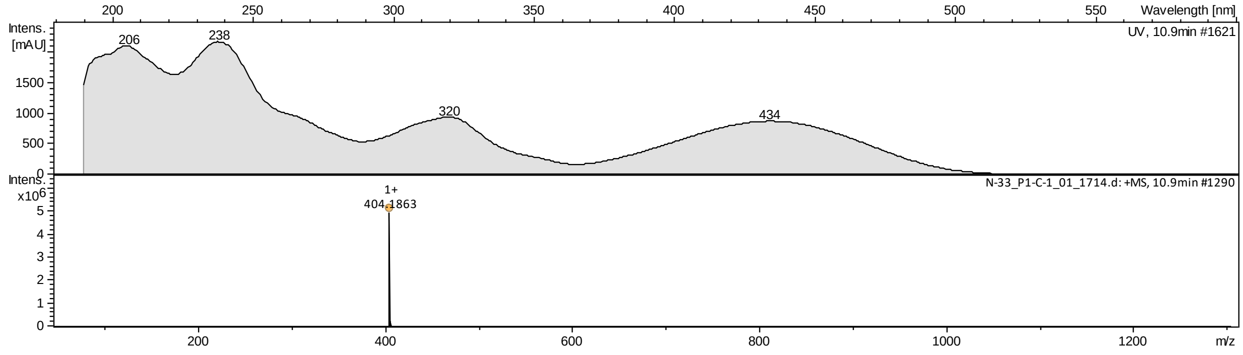

^{1}H NMR spectrum (500 MHz, acetone-d_6) of **35**

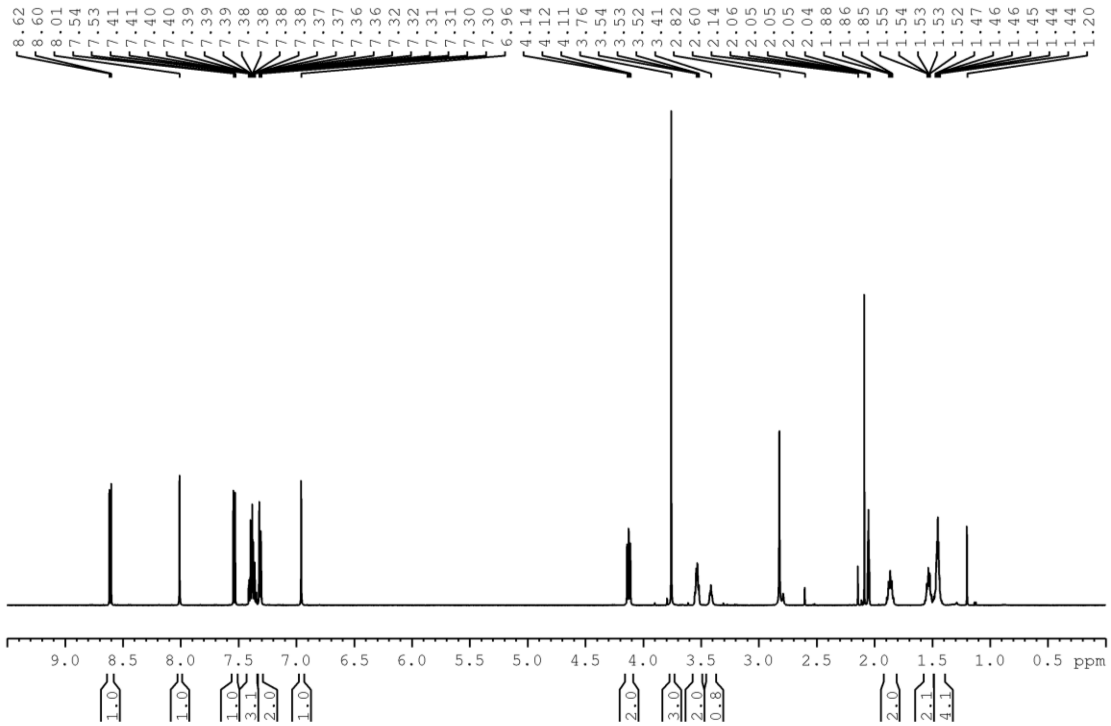

^{13}C NMR spectrum (125 MHz, acetone-d_6) of **35**

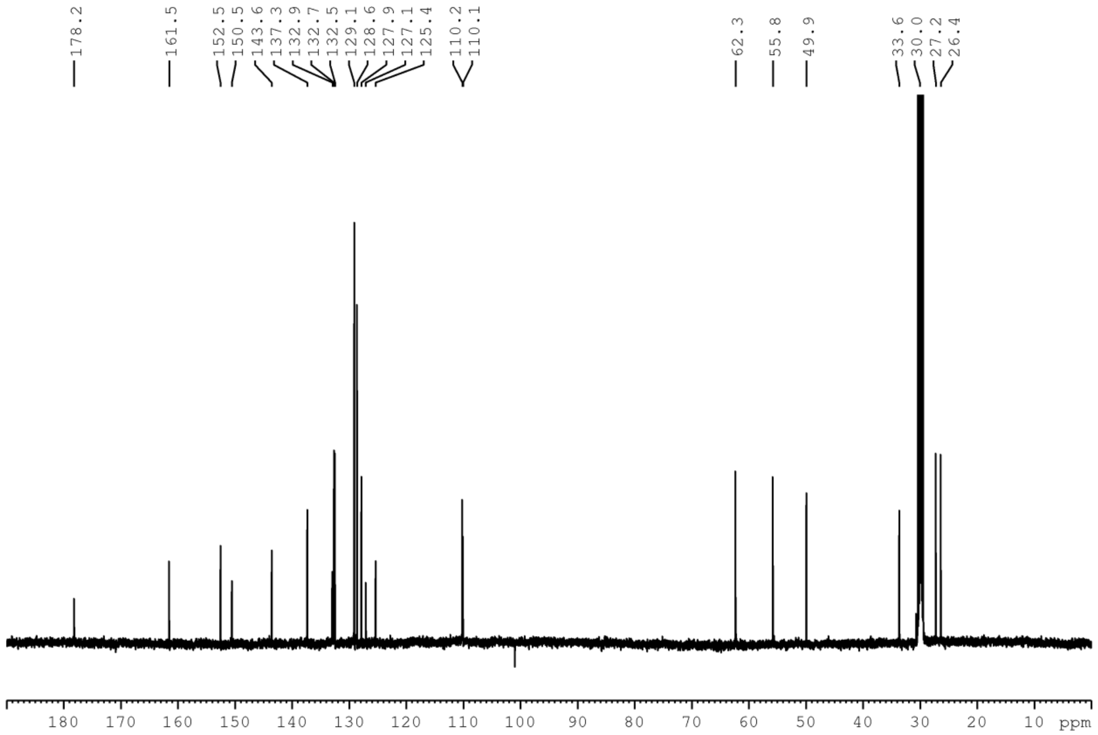

HSQC spectrum (500 MHz, acetone-d_6) of **35**

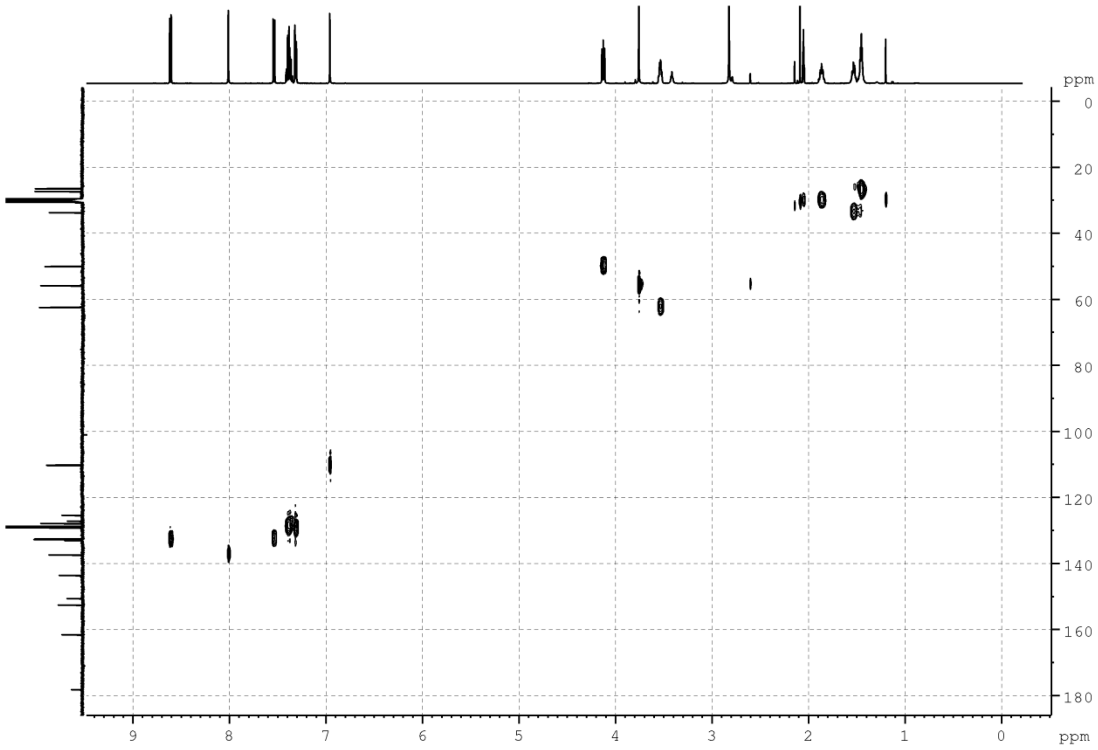

HMBC spectrum (500 MHz, acetone-d_6) of **35**

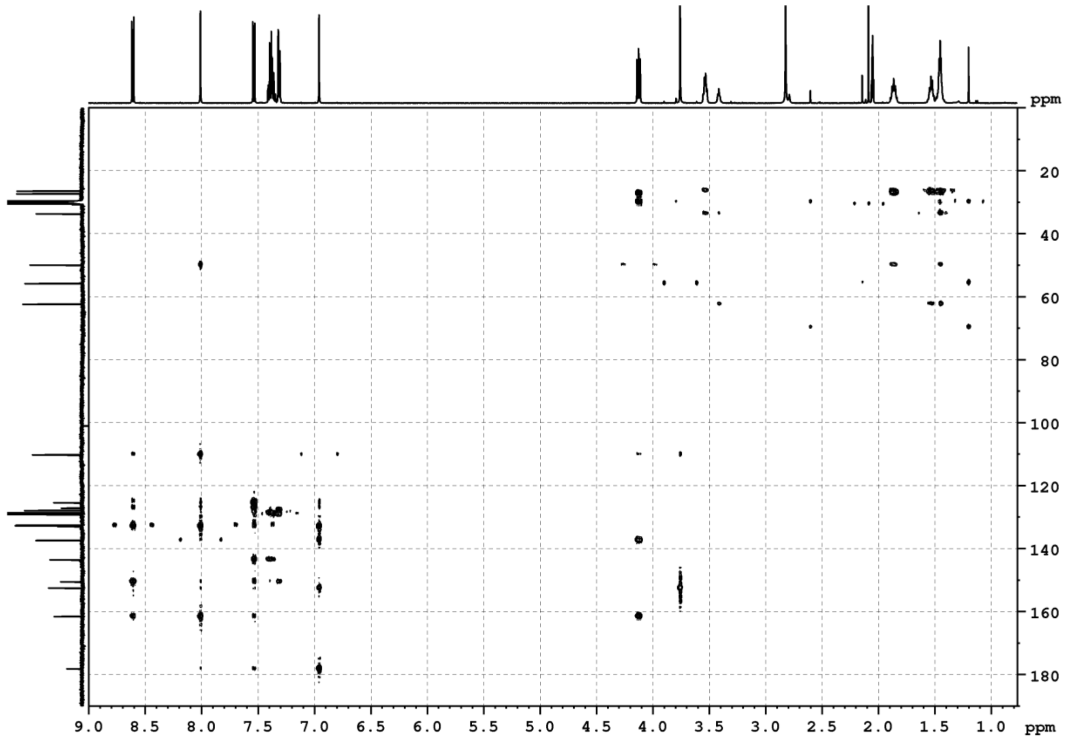

Scheme SS6. Experimental HRESIMS and NMR spectra of **36**

2-(8''-Hydroxyoctyl)-5-hydroxy-7-phenyl-2H-benzo[de]isoquinoline-1,6-dione (36)

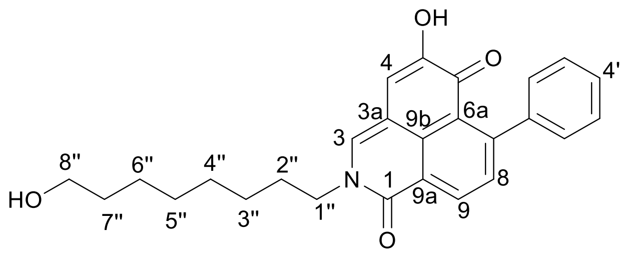

Structure of **36**

HPLC-PDA-HRESIMS (method B, gradient 2) chromatogram of **36** (UV 254 nm)

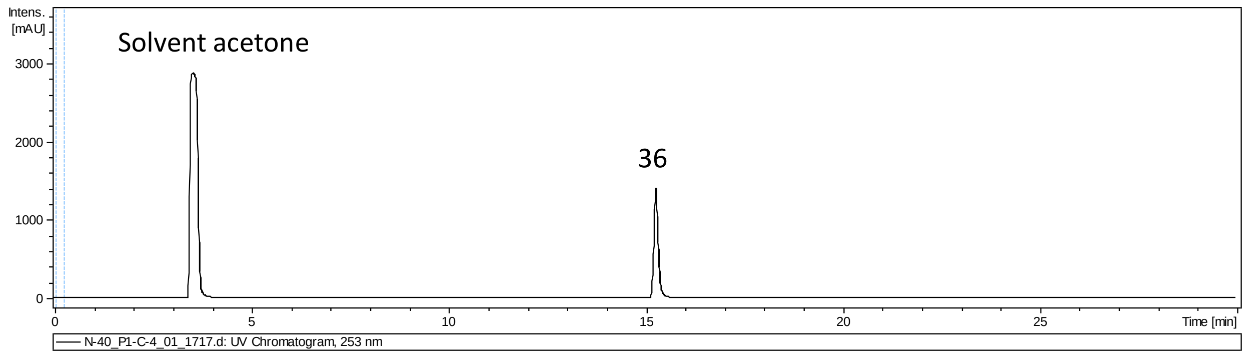

UV and HRESIMS spectrum of **36**

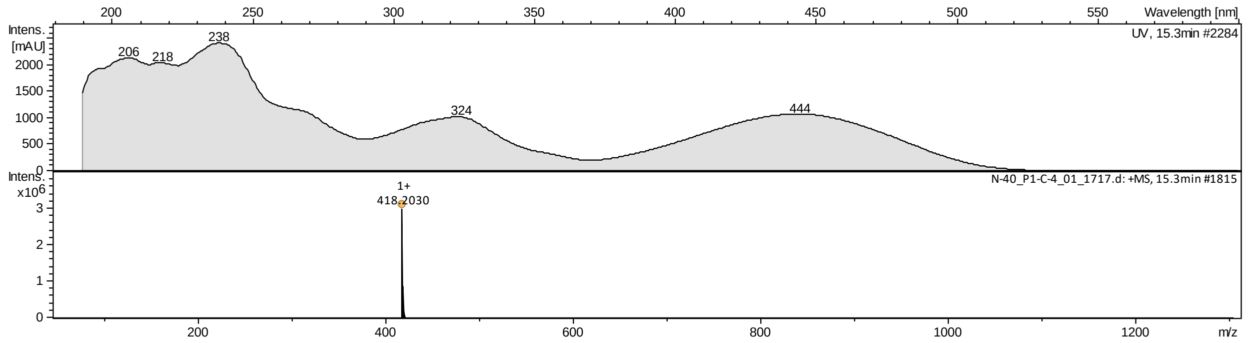

^{1}H NMR spectrum (500 MHz, chloroform-*d*) of **36**

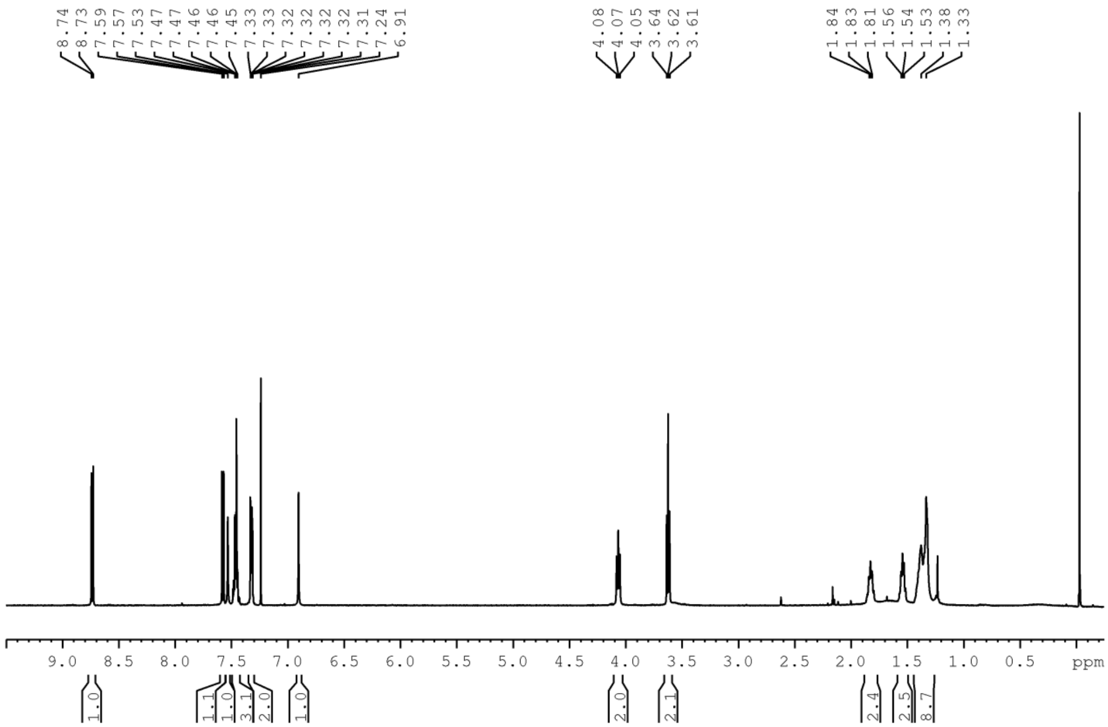

^{13}C NMR spectrum (125 MHz, chloroform-*d*) of **36**

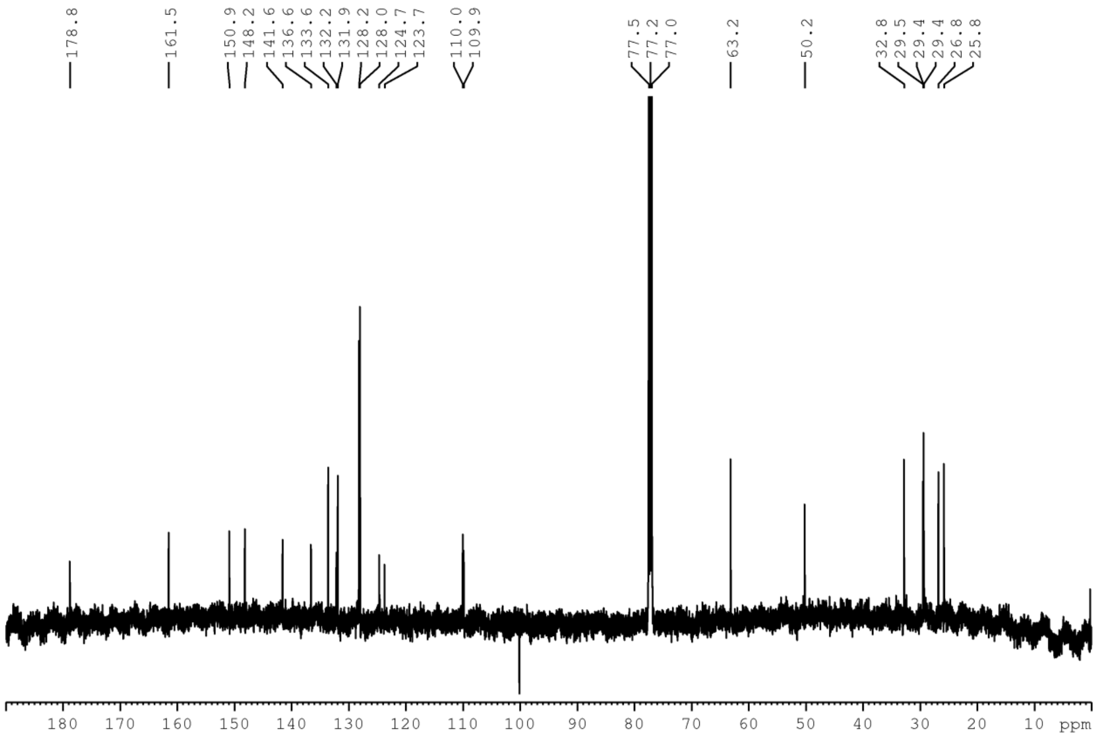

COSY spectrum (500 MHz, chloroform-*d*) of **36**

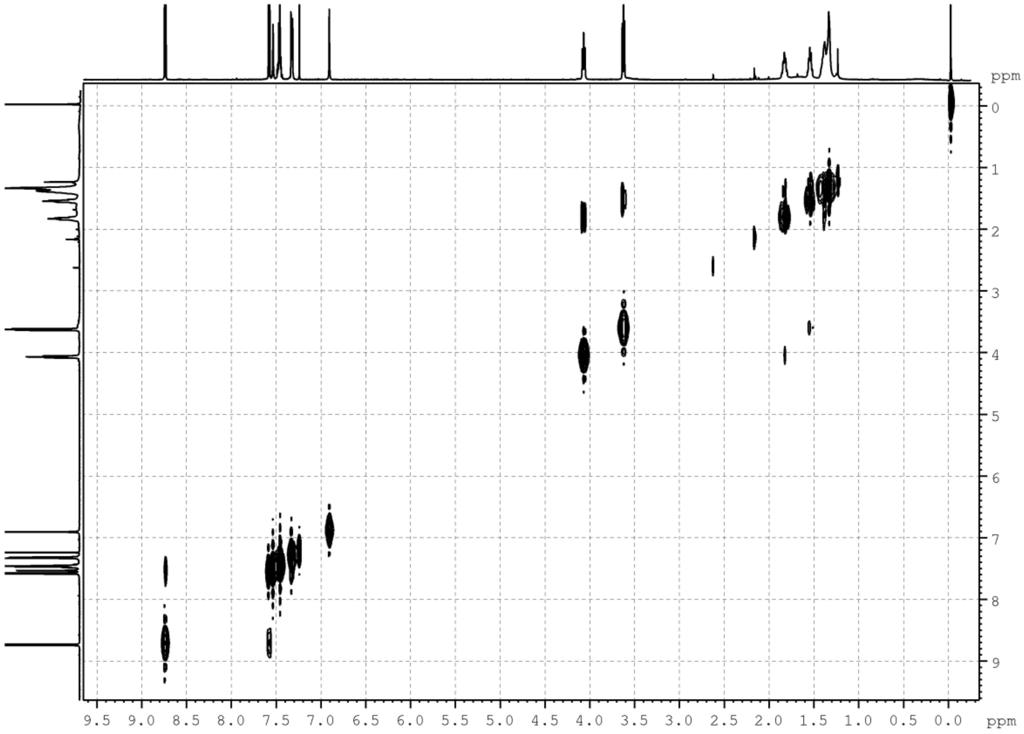

HSQC spectrum (500 MHz, chloroform-*d*) of **36**

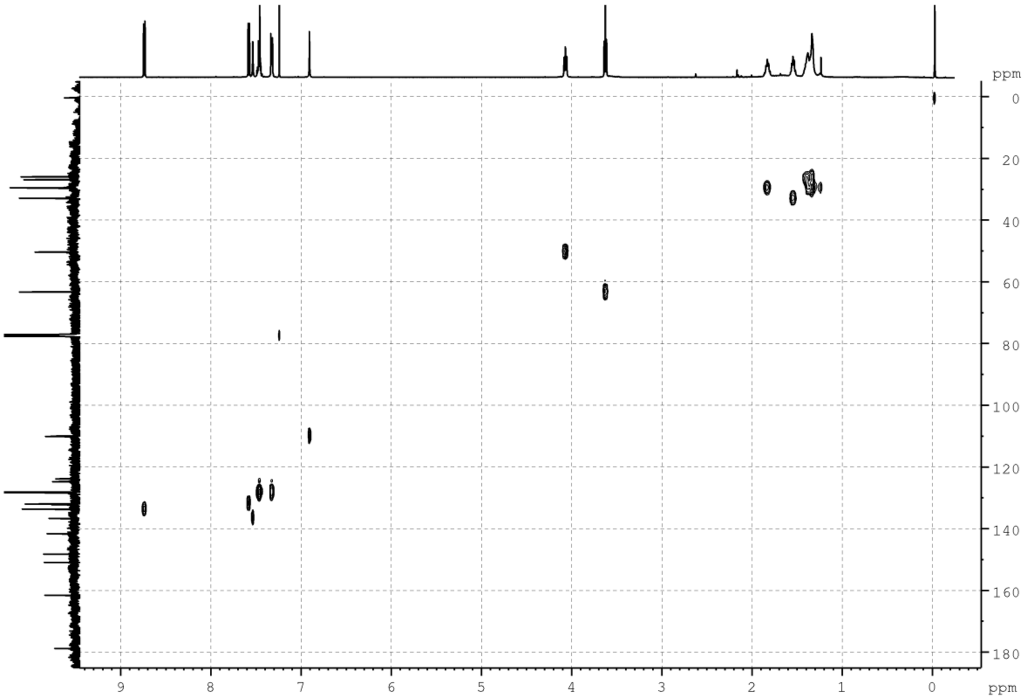

HMBC spectrum (500 MHz, chloroform-*d*) of **36**

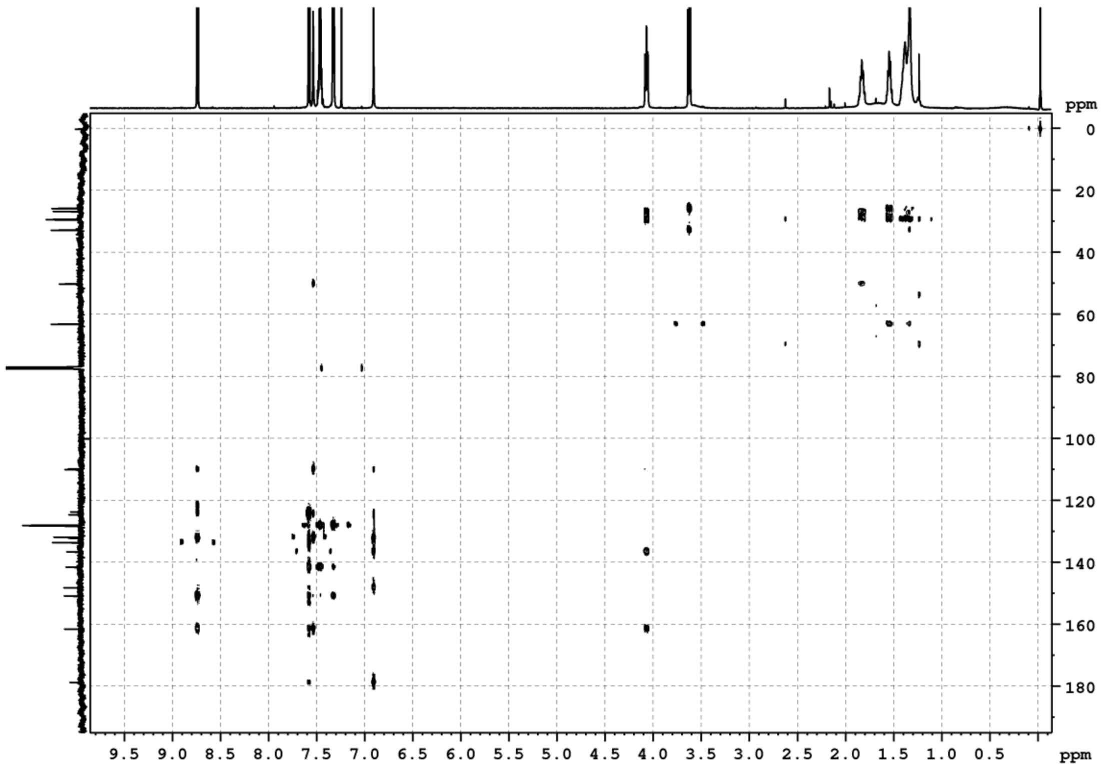

Scheme SS7. Experimental HRESIMS and NMR spectra of **37**

2-(8''-Hydroxyoctyl)-5-methoxy-7-phenyl-2H-benzo[de]isoquinoline-1,6-dione (37)

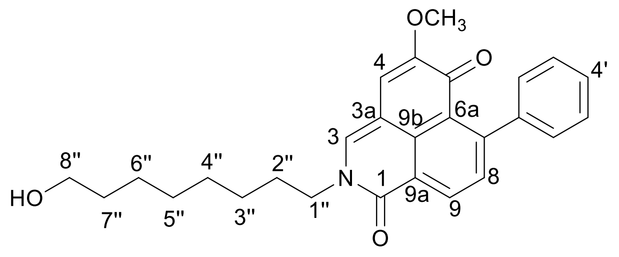

Structure of **37**

HPLC-PDA-HRESIMS (method B, gradient 2) chromatogram of **37** (UV 254 nm)

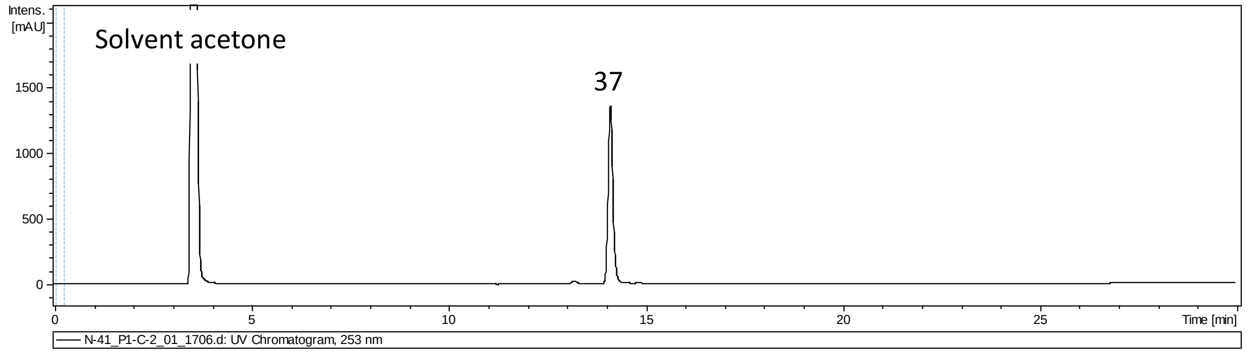

UV and HRESIMS spectrum of **37**

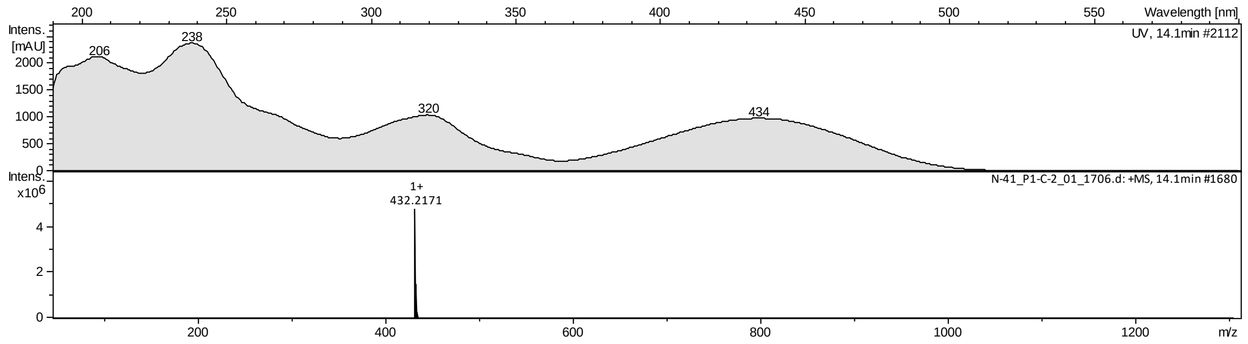

^{1}H NMR spectrum (500 MHz, chloroform-*d*) of **37**

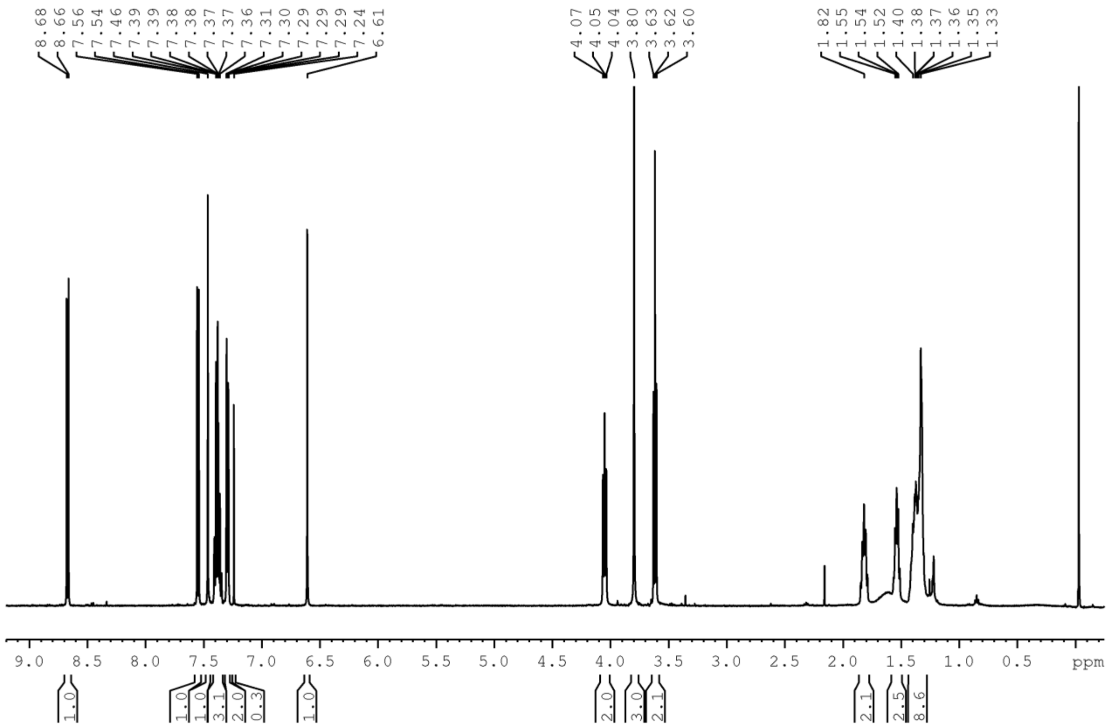

^{13}C NMR spectrum (125 MHz, chloroform-*d*) of **37**

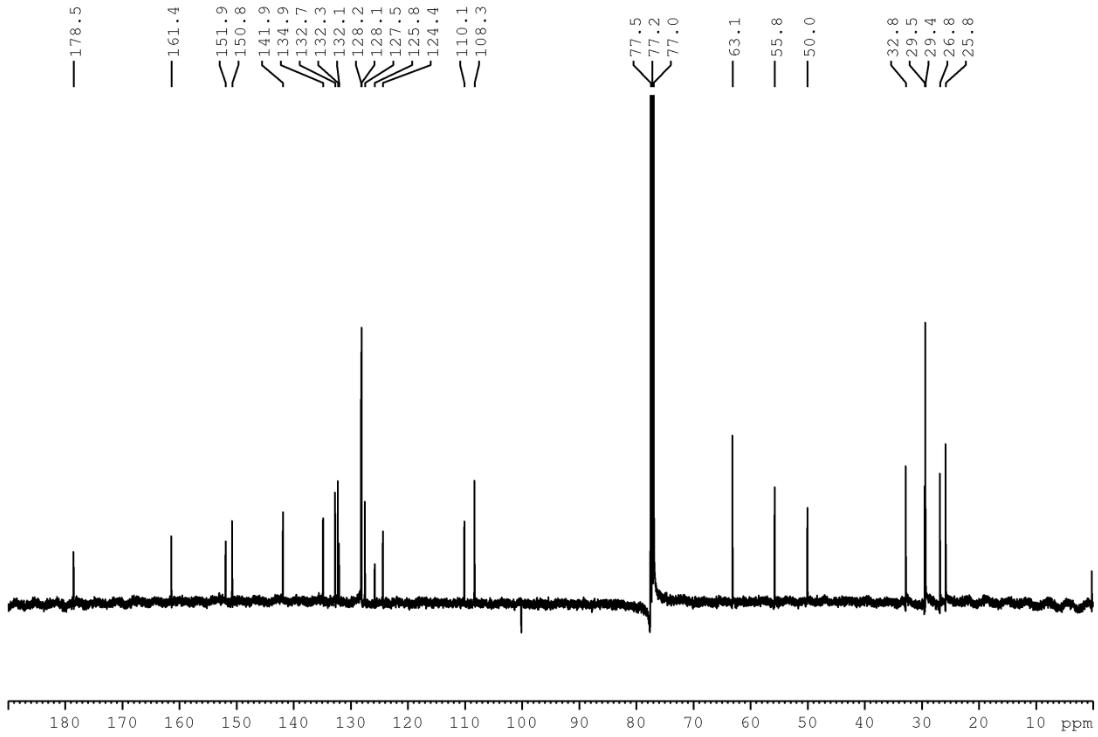

COSY spectrum (500 MHz, chloroform-*d*) of **37**

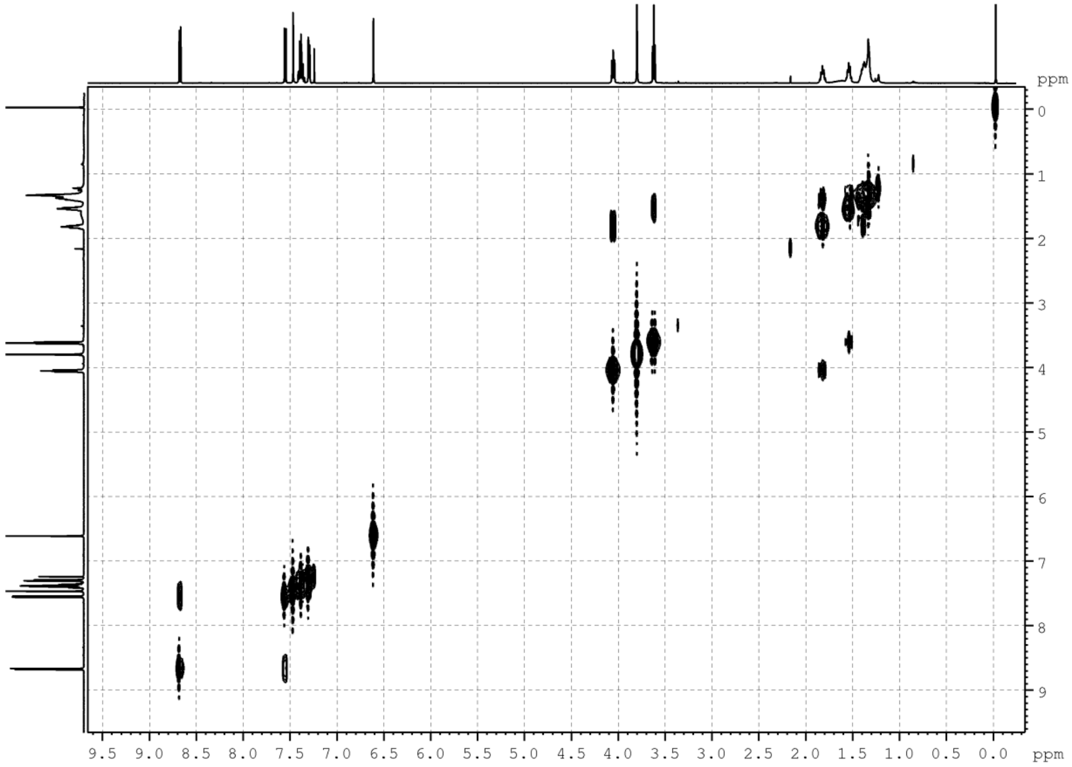

HSQC spectrum (500 MHz, chloroform-*d*) of **37**

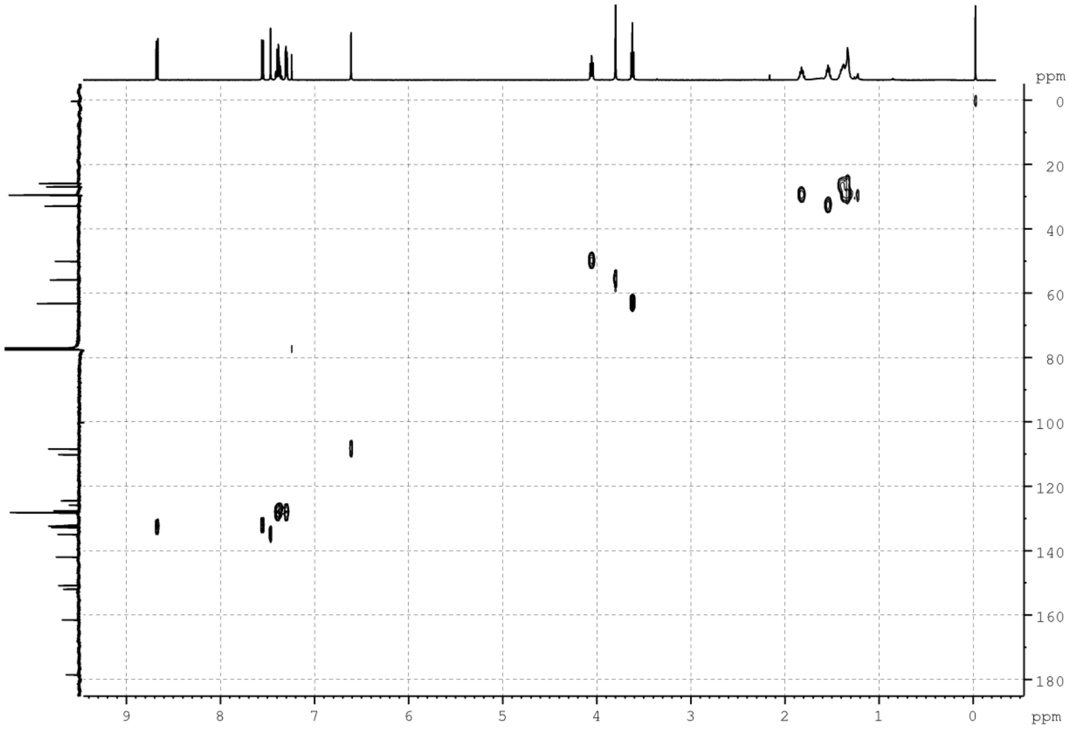

HMBC spectrum (500 MHz, chloroform-*d*) of **37**

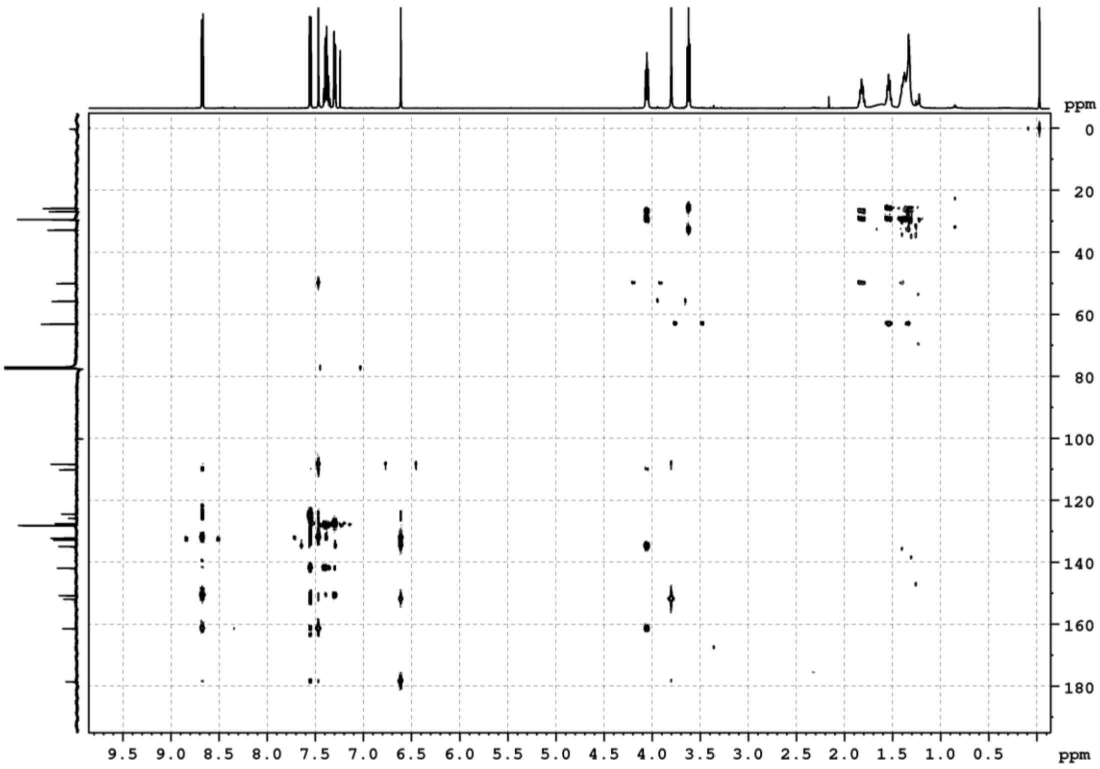

Scheme SS8. Experimental HRESIMS and NMR spectra of **38**

2-(10''-Hydroxydecyl)-5-hydroxy-7-phenyl-2H-benzo[de]isoquinoline-1,6-dione (38)

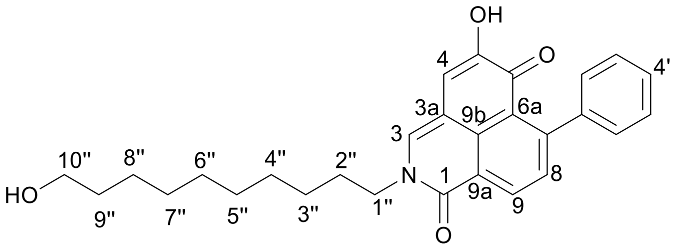

Structure of **38**

HPLC-PDA-HRESIMS (method B, gradient 2) chromatogram of **38** (UV 254 nm)

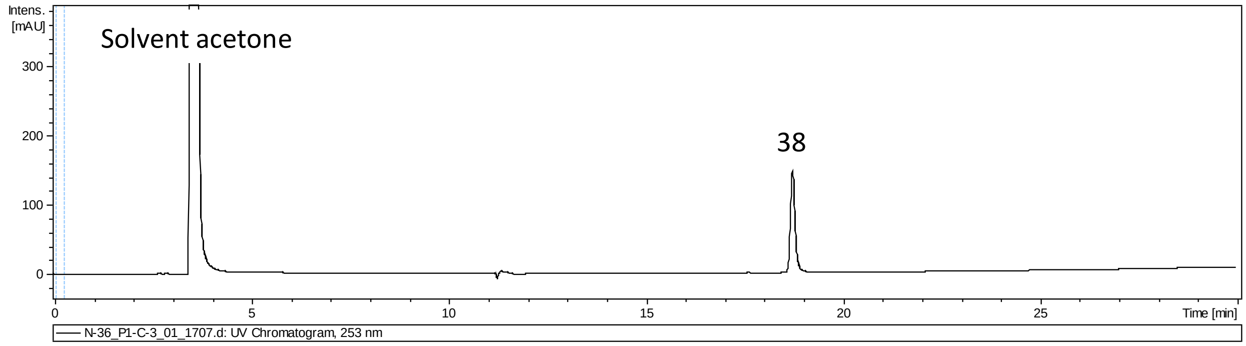

UV and HRESIMS spectrum of **38**

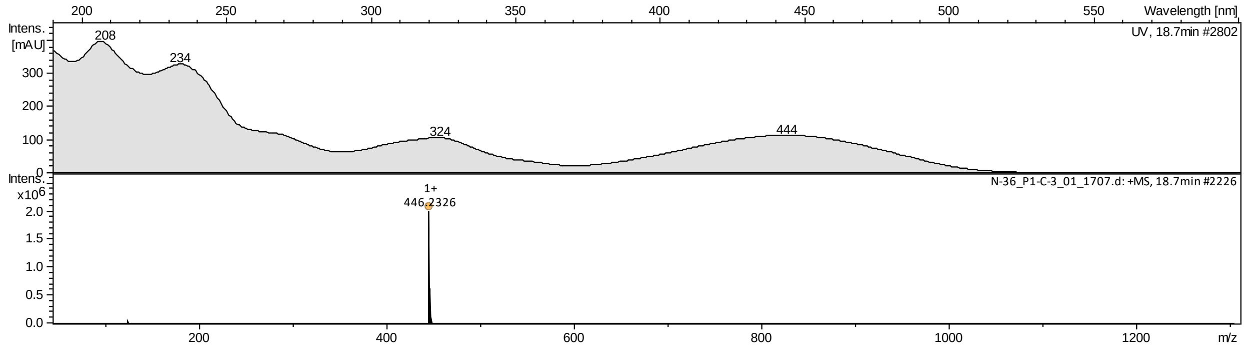

^{1}H NMR spectrum (500 MHz, acetone-d_6) of **38**

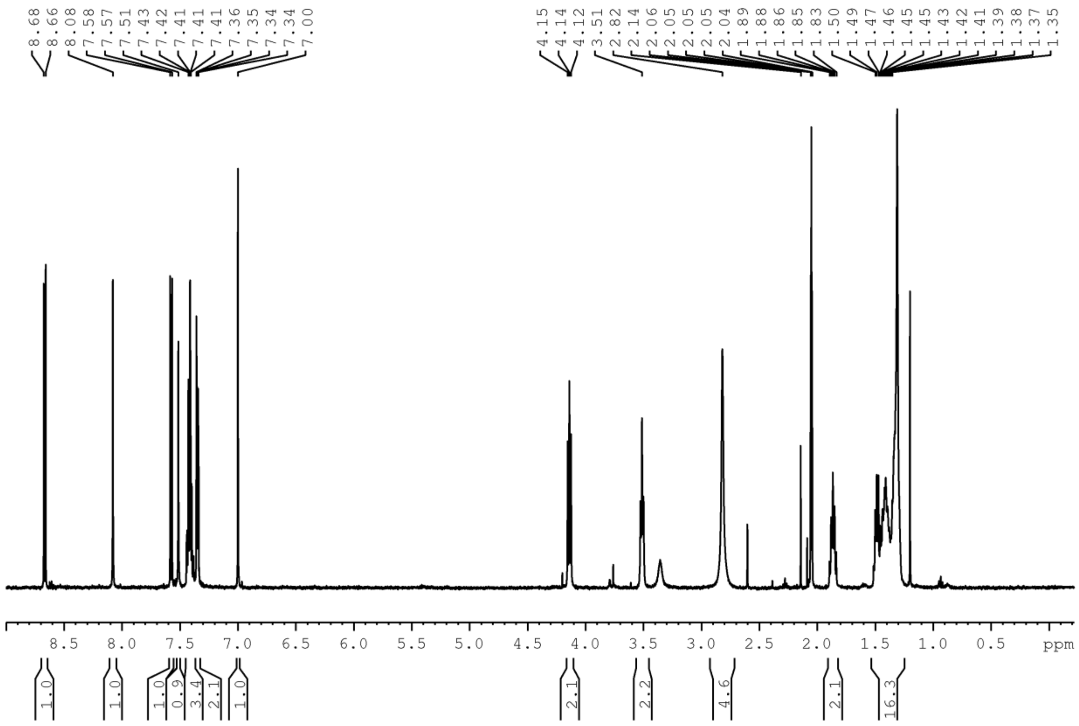

^{13}C NMR spectrum (125 MHz, acetone-d_6) of **38**

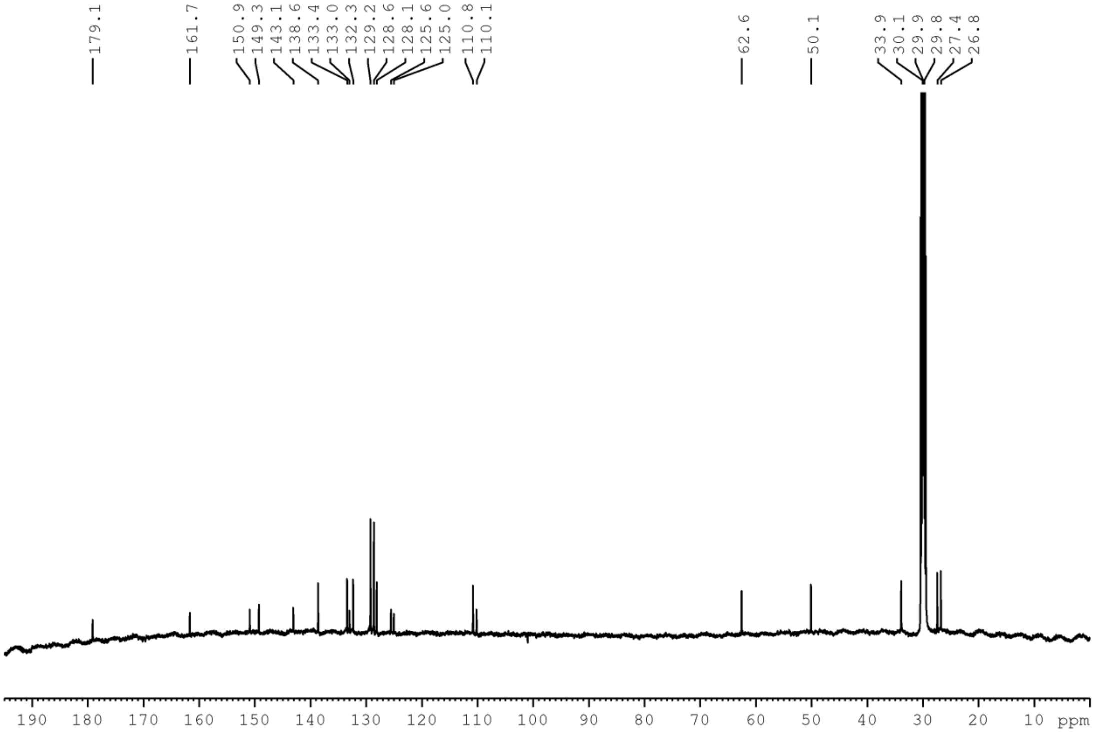

COSY spectrum (500 MHz, acetone-d_6) of **38**

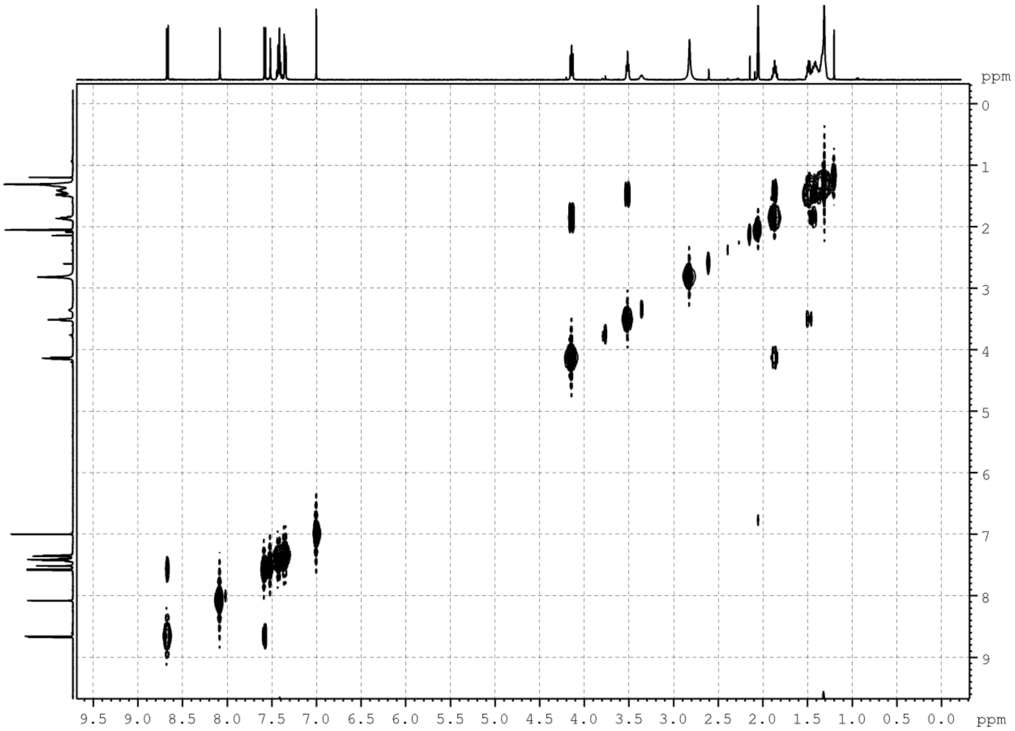

HSQC spectrum (500 MHz, acetone-d_6) of **38**

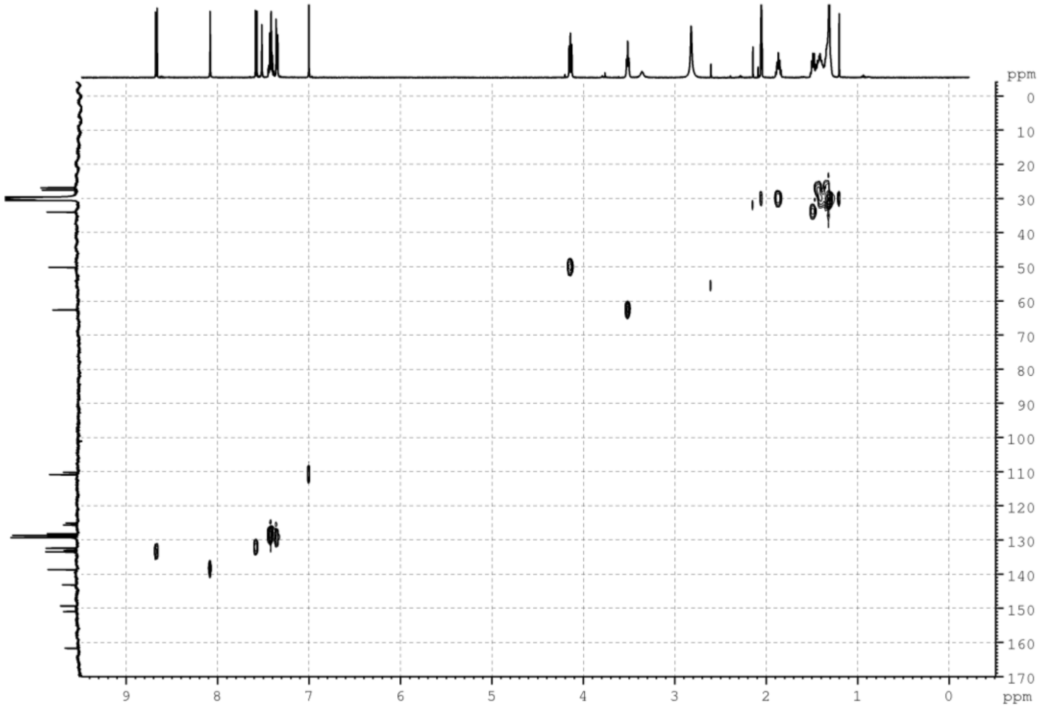

HMBC spectrum (500 MHz, acetone-d_6) of **38**

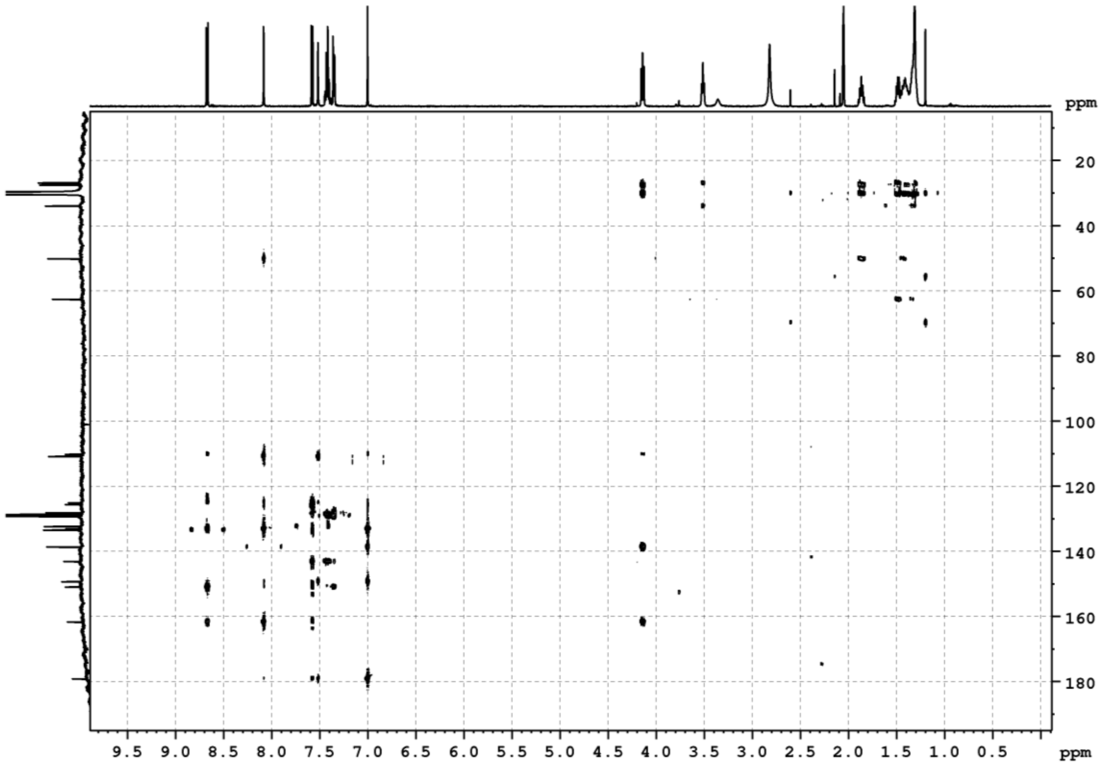

Scheme SS9. Experimental HRESIMS and NMR spectra of **39**

2-(10''-Hydroxydecyl)-5-methoxy-7-phenyl-2H-benzo[de]isoquinoline-1,6-dione (39)

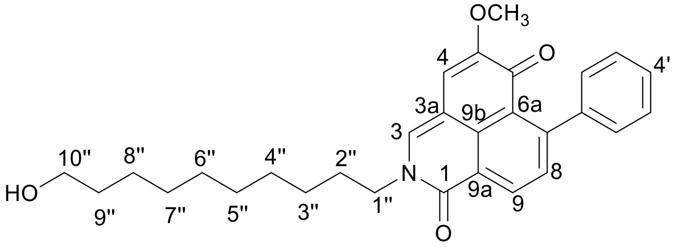

Structure of **39**

HPLC-PDA-HRESIMS (method B, gradient 2) chromatogram of **39** (UV 254 nm)

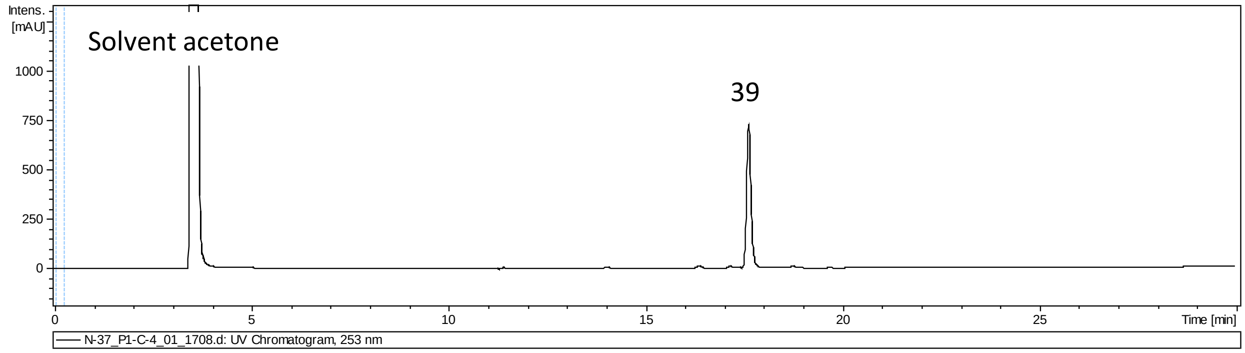

UV and HRESIMS spectrum of **39**

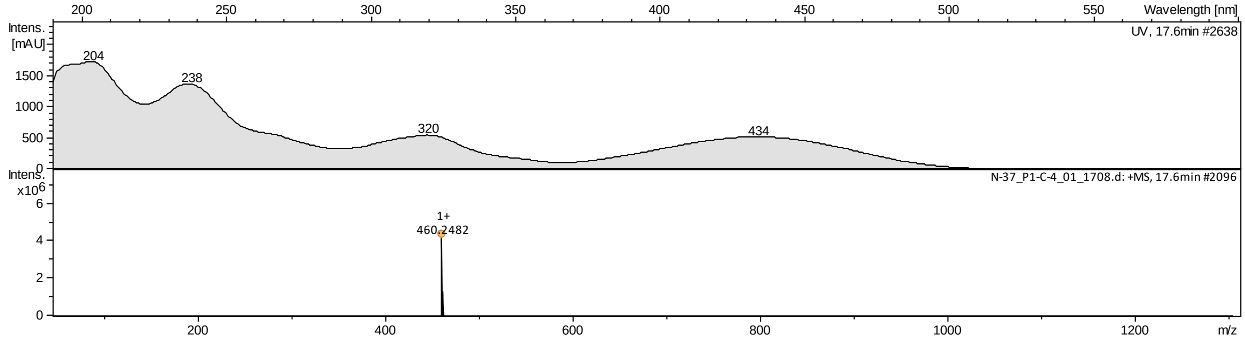

^{1}H NMR spectrum (500 MHz, acetone-d_6) of **39**

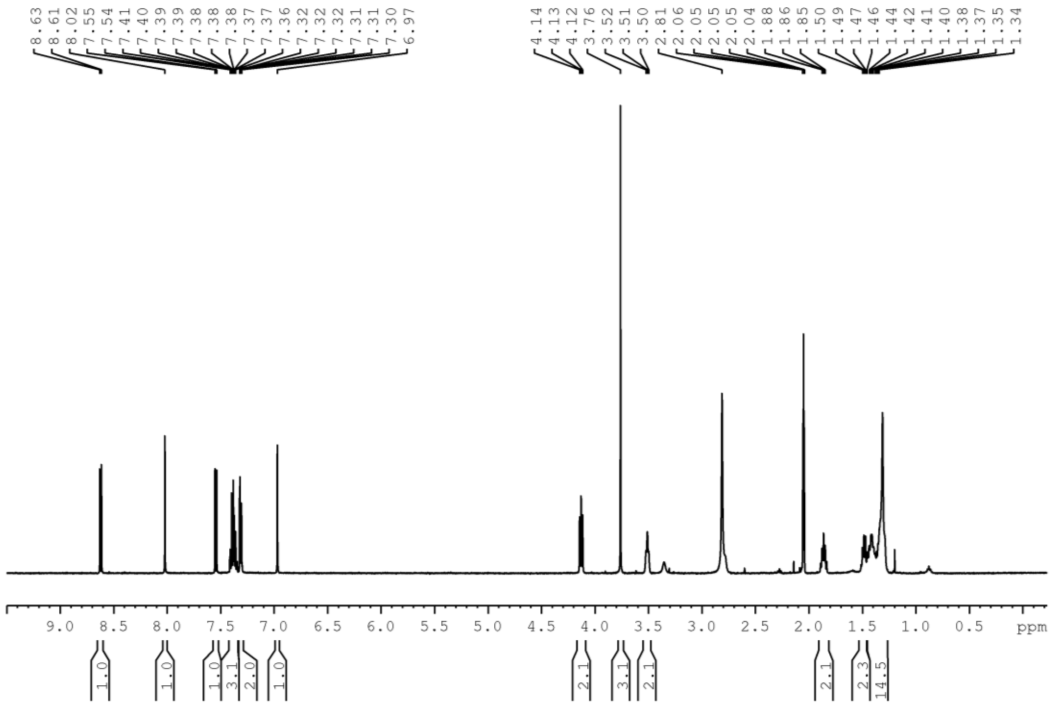

COSY spectrum (500 MHz, acetone-d_6) of **39**

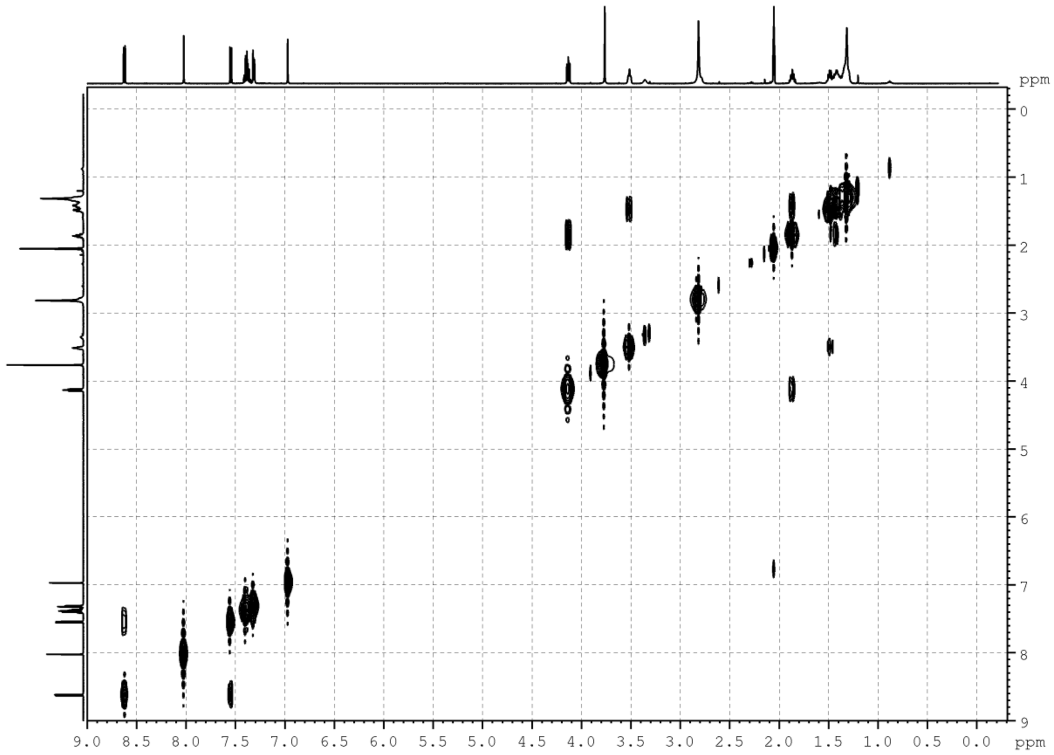

HSQC spectrum (500 MHz, acetone-d_6) of **39**

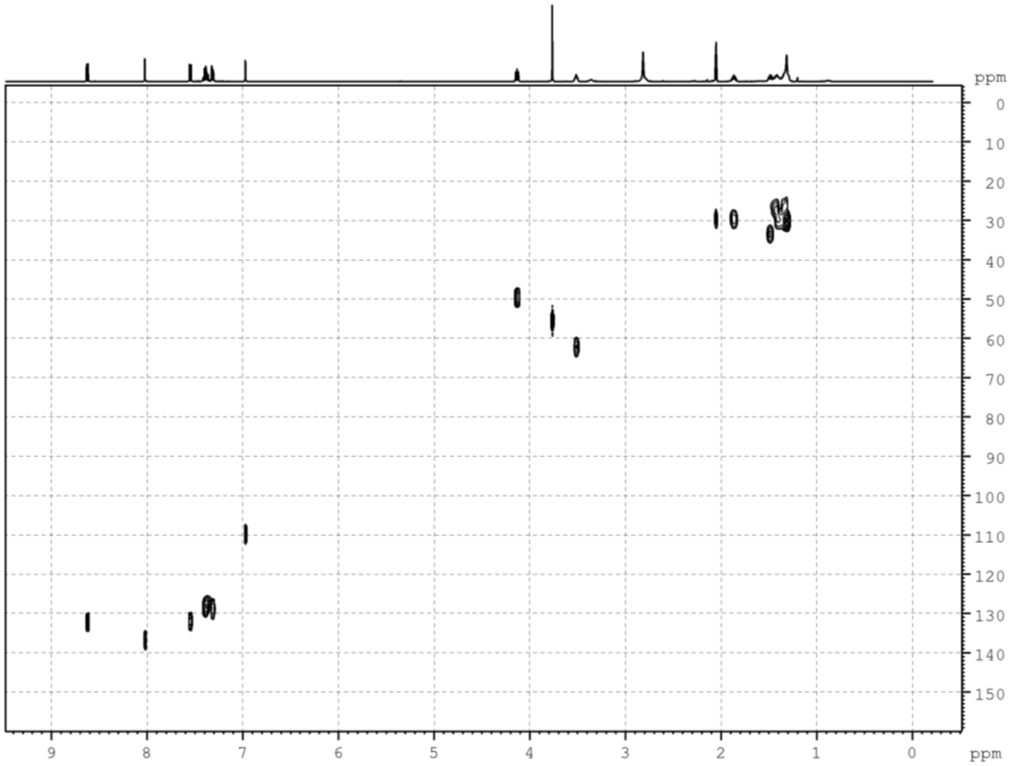

HMBC spectrum (500 MHz, acetone-d_6) of **39**

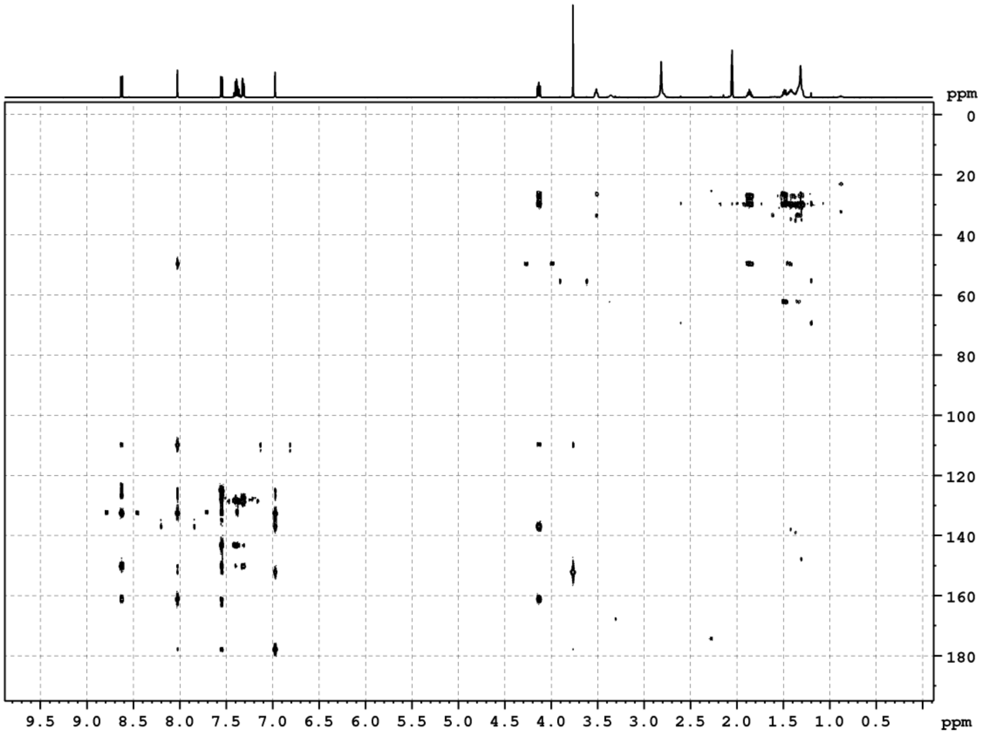

Scheme SS10. Experimental HRESIMS and NMR spectra of **40**

2-(12''-Hydroxydodecyl)-5-hydroxy-7-phenyl-2H-benzo[de]isoquinoline-1,6-dione (40)

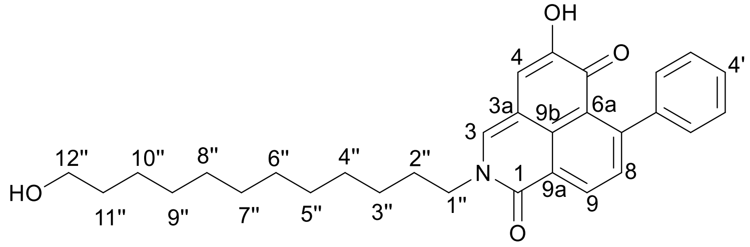

Structure of **40**

HPLC-PDA-HRESIMS (method B, gradient 2) chromatogram of **40** (UV 254 nm)

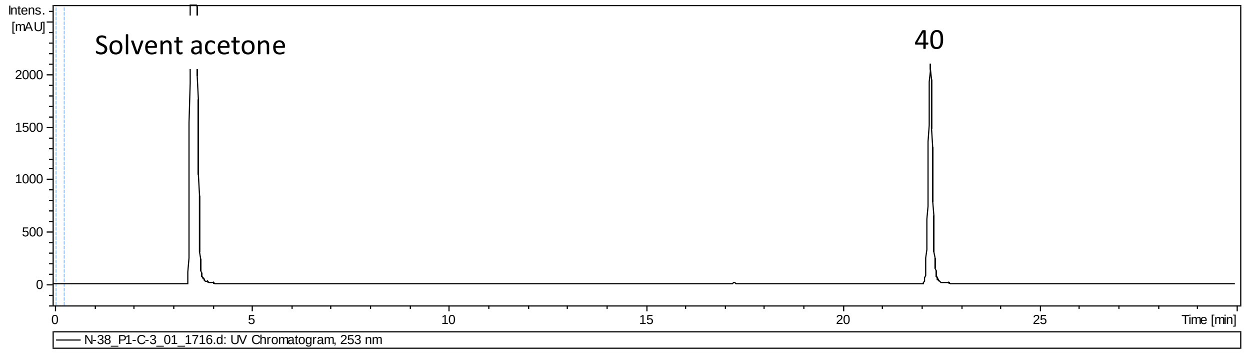

UV and HRESIMS spectrum of **40**

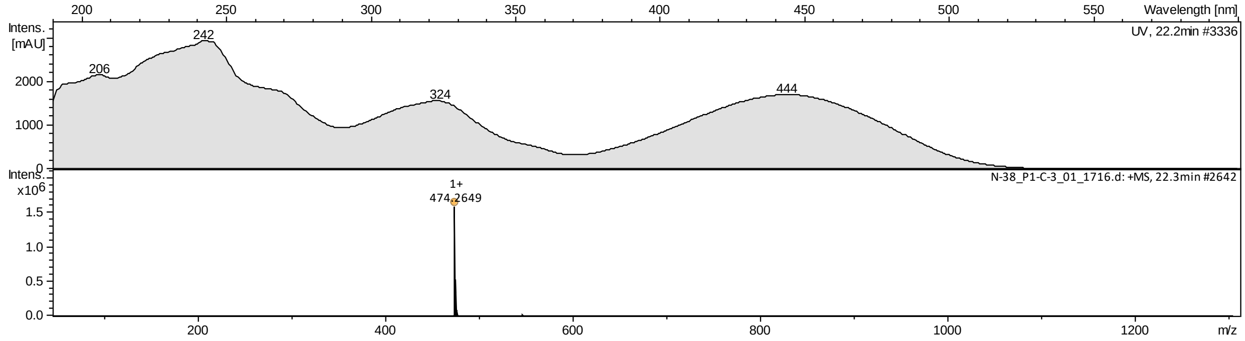

^{1}H NMR spectrum (500 MHz, chloroform-*d*) of **40**

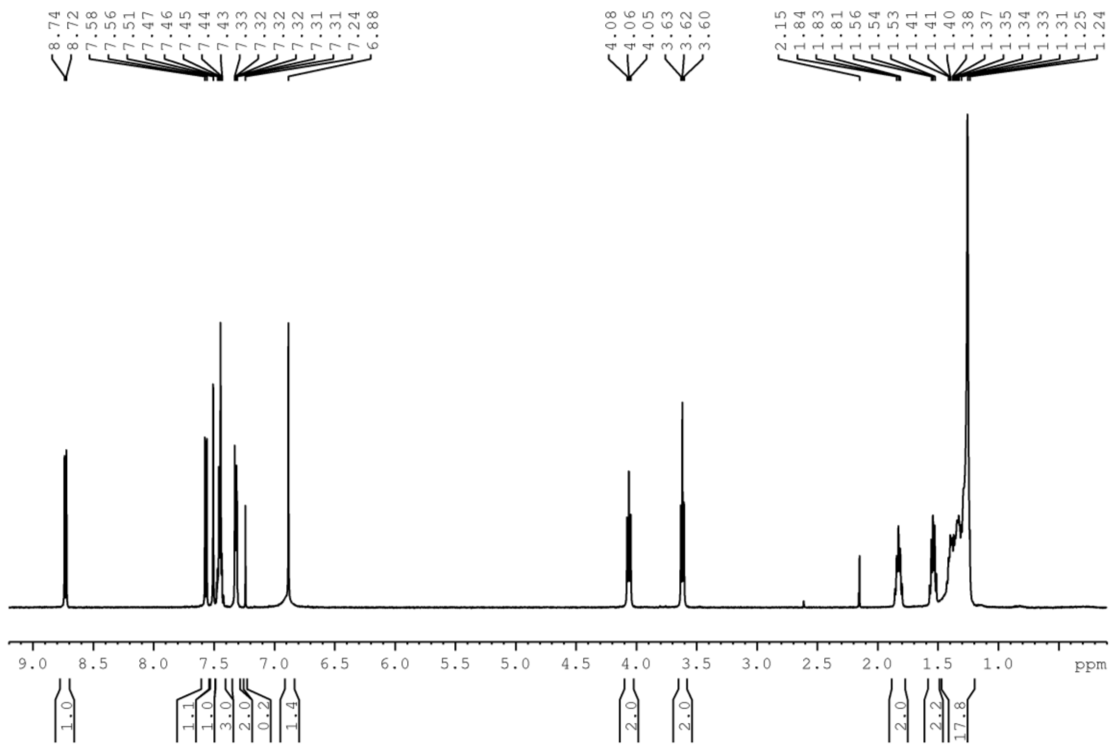

^{13}C NMR spectrum (125 MHz, chloroform-*d*) of **40**

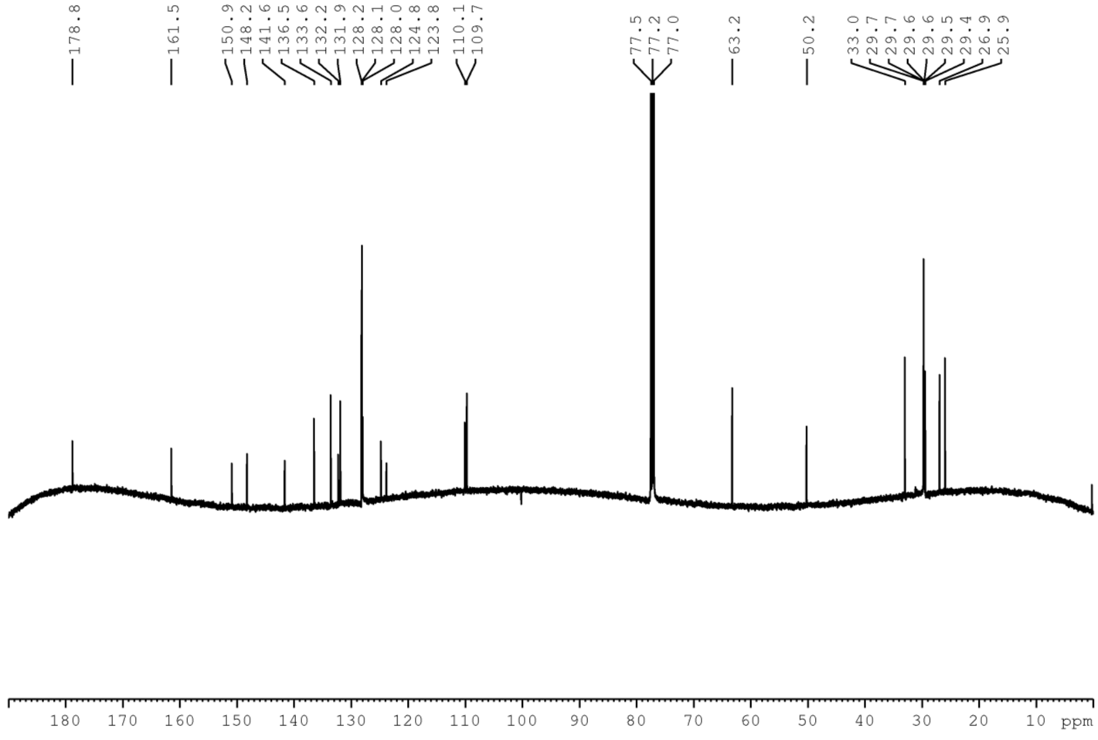

COSY spectrum (500 MHz, chloroform-*d*) of **40**

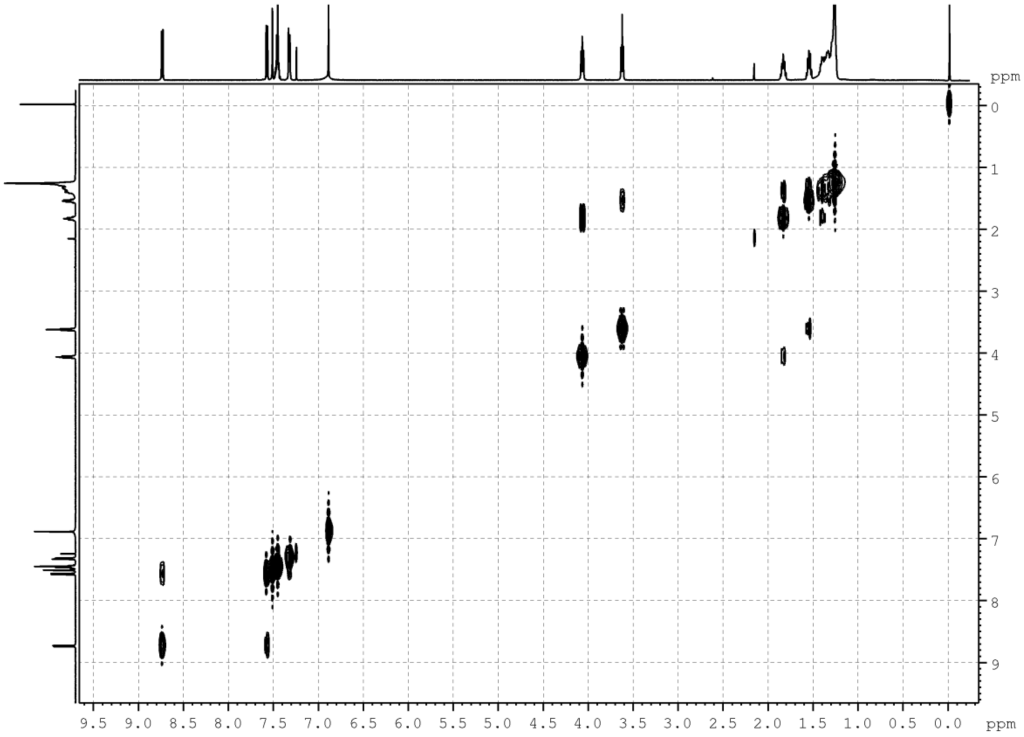

HSQC spectrum (500 MHz, chloroform-*d*) of **40**

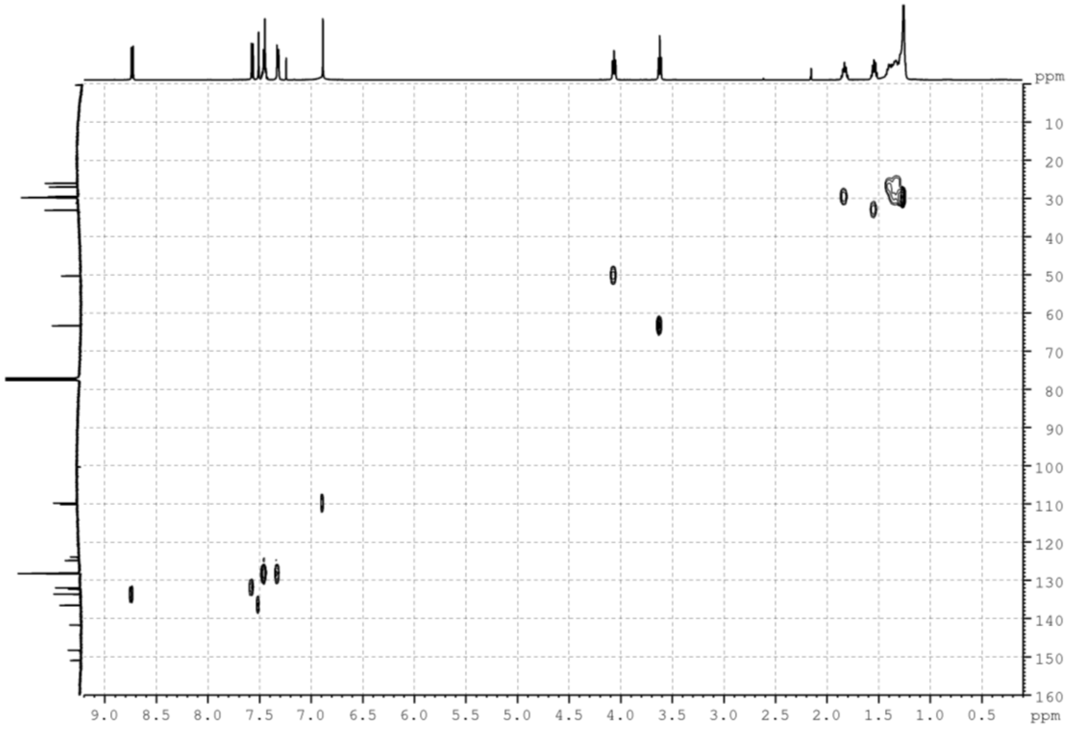

HMBC spectrum (500 MHz, chloroform-*d*) of **40**

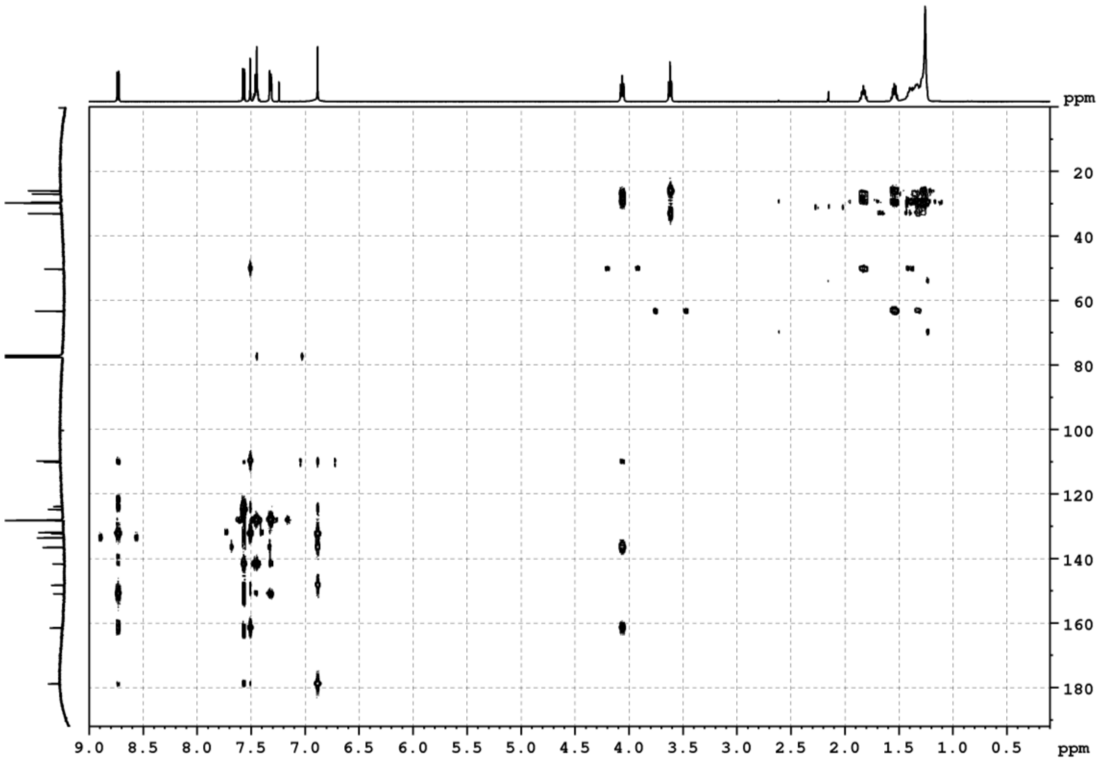

Scheme SS11. Experimental HRESIMS and NMR spectra of **41**

2-(12''-Hydroxydodecyl)-5-hydroxy-7-phenyl-2H-benzo[de]isoquinoline-1,6-dione (41)

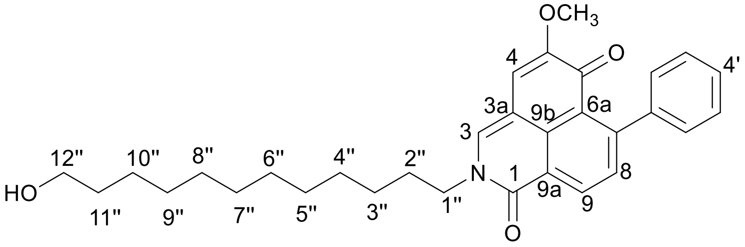

Structure of **41**

HPLC-PDA-HRESIMS (method B, gradient 2) chromatogram of **41** (UV 254 nm)

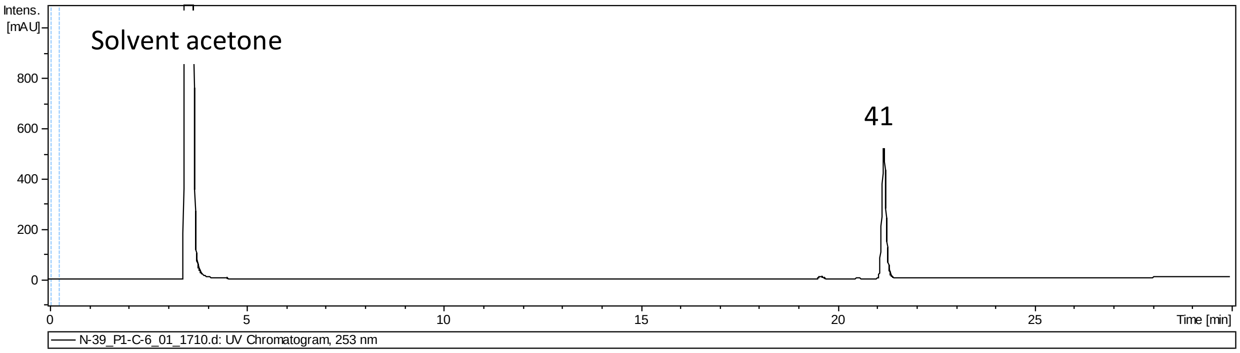

UV and HRESIMS spectrum of **41**

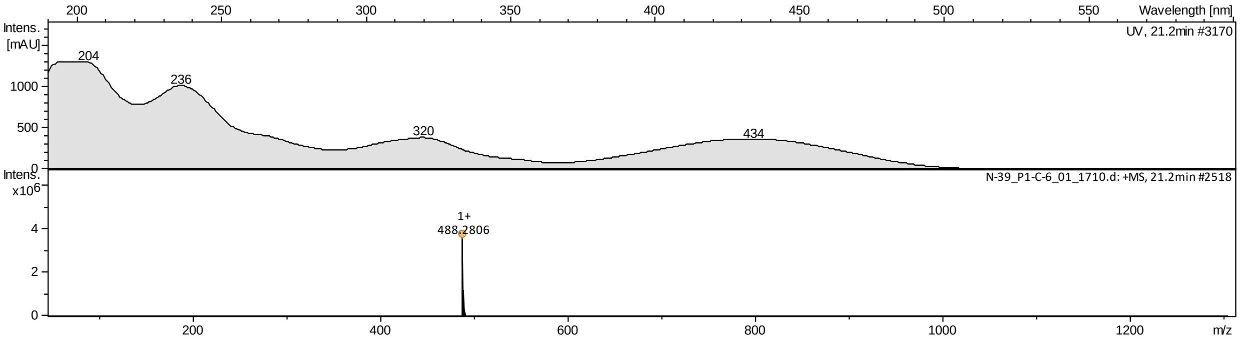

^{1}H NMR spectrum (500 MHz, chloroform-*d*) of **41**

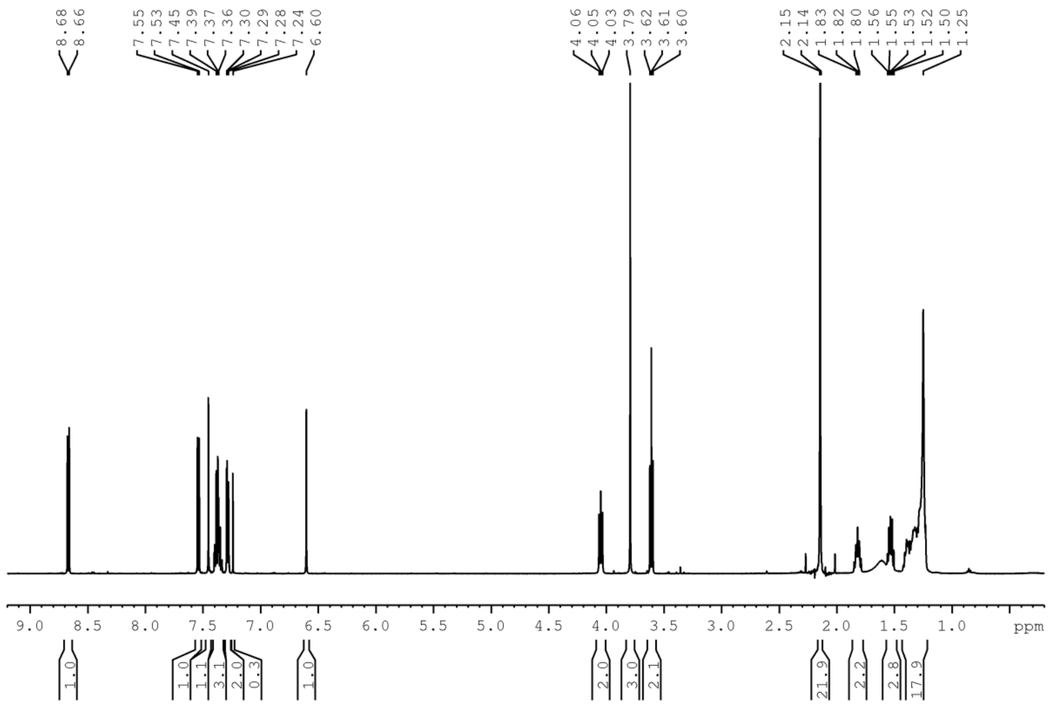

^{13}C NMR spectrum (125 MHz, chloroform-*d*) of **41**

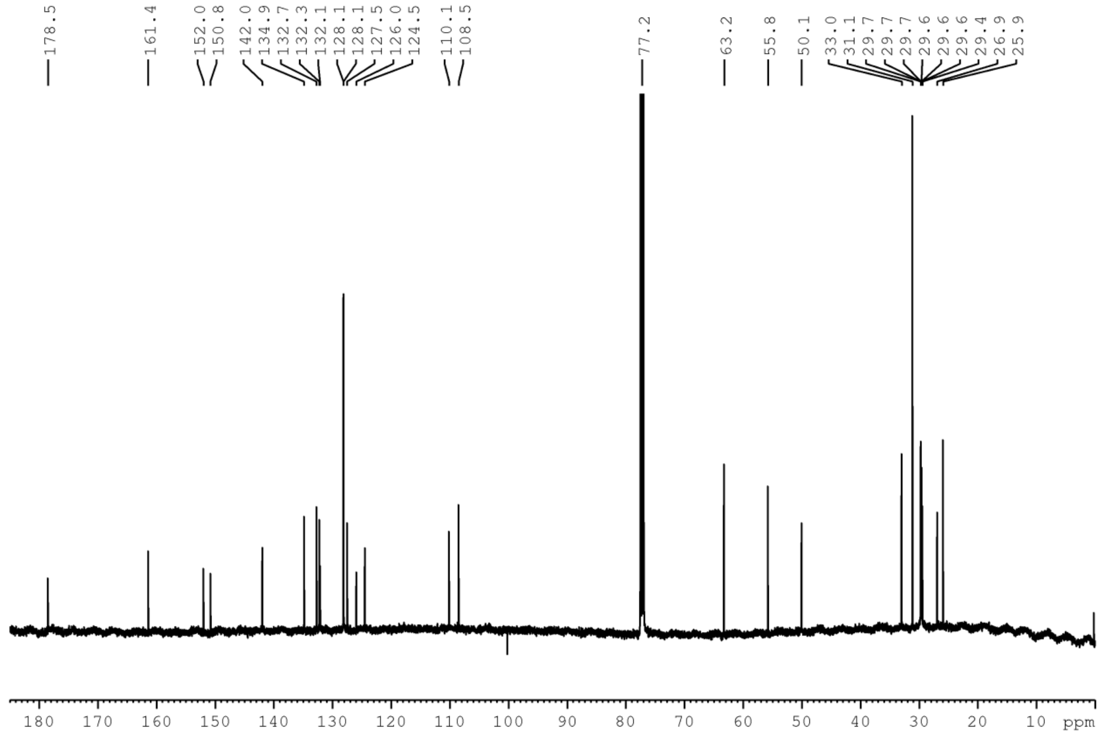

COSY spectrum (500 MHz, chloroform-*d*) of **41**

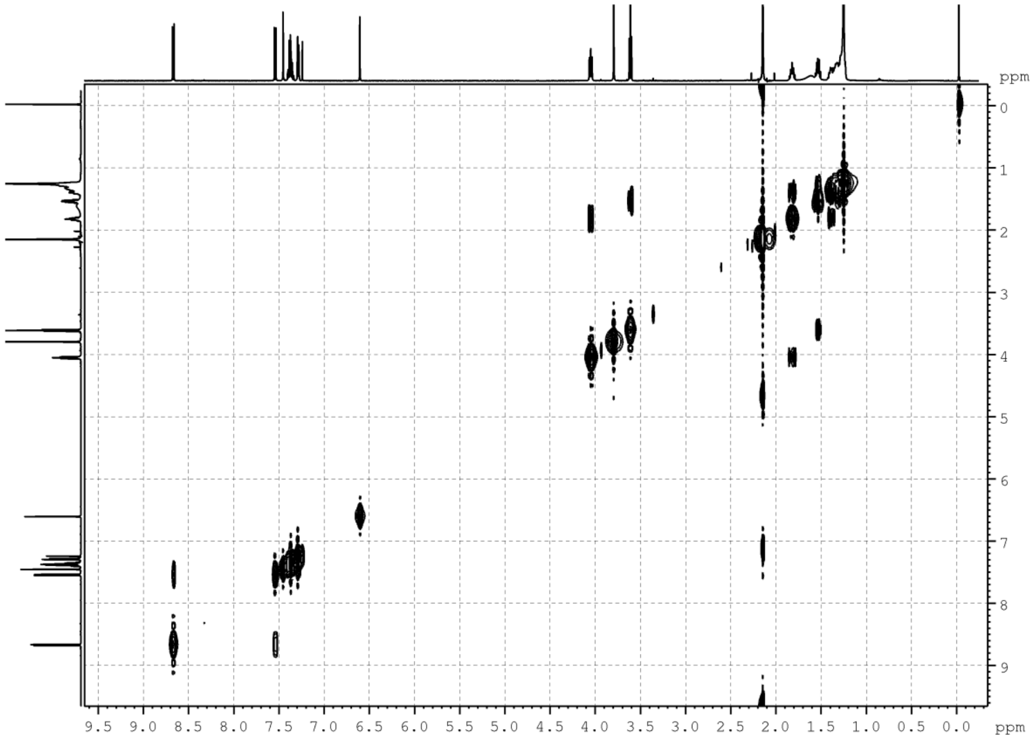

HSQC spectrum (500 MHz, chloroform-*d*) of **41**

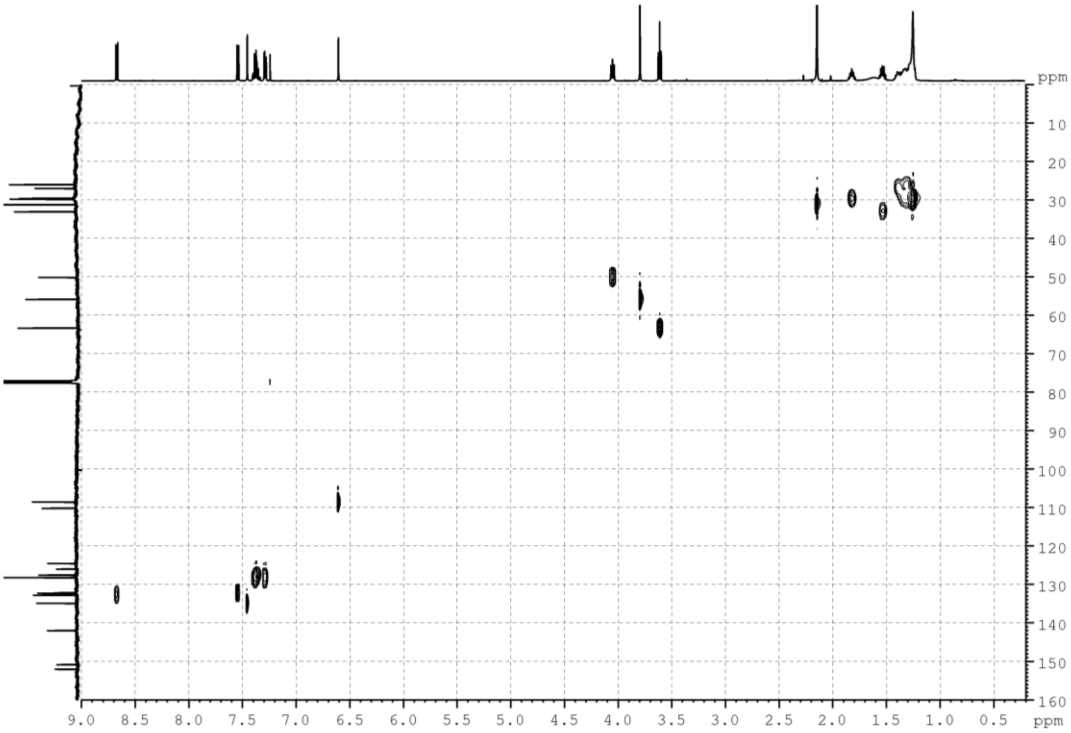

HMBC spectrum (500 MHz, chloroform-*d*) of **41**

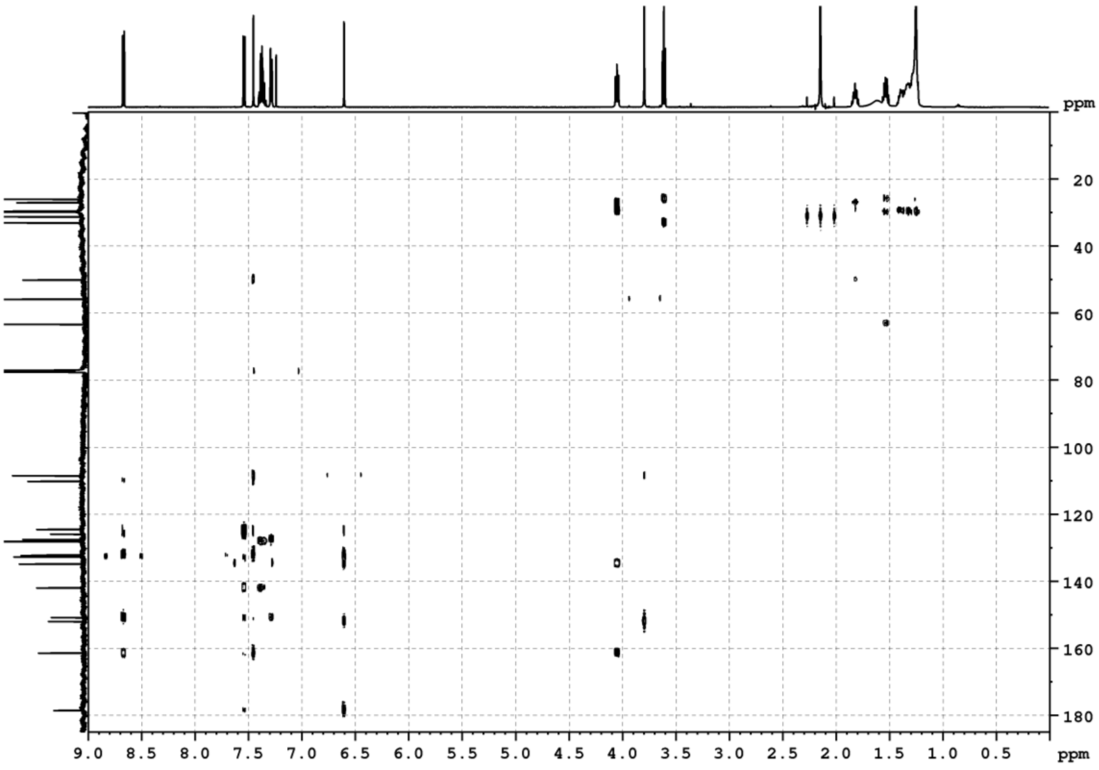

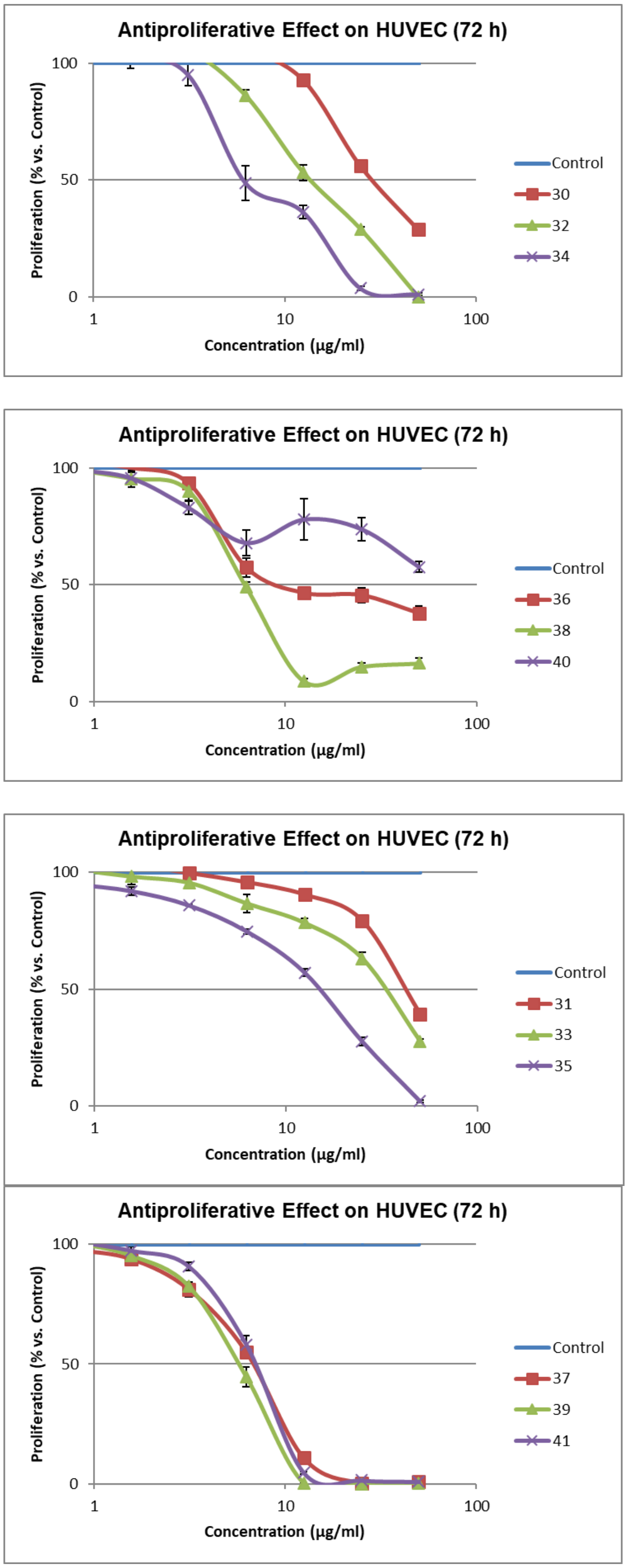

Figure SF3. Test for antiproliferative effects of compounds **30** – **41** on HUVEC cells (72 h).

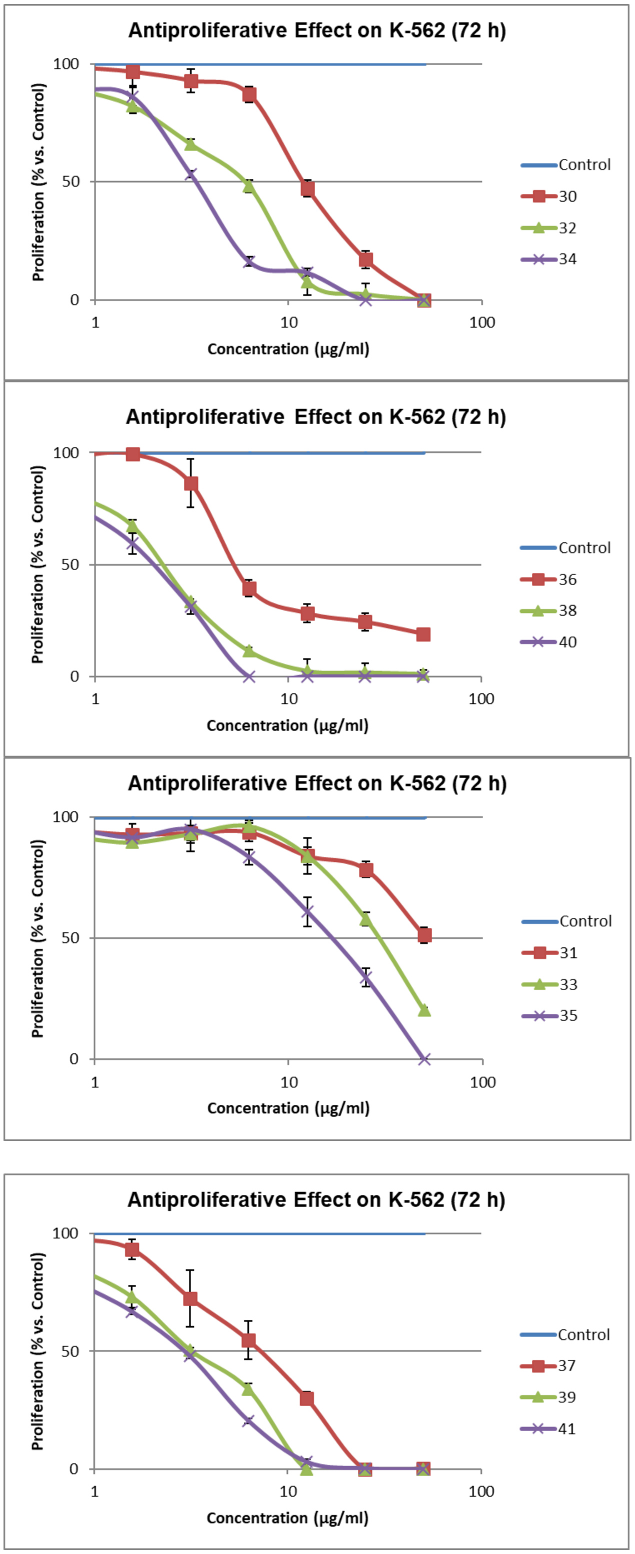

Figure SF4. Test for antiproliferative effects of compounds **30** – **41** on K-562 cells (72 h).

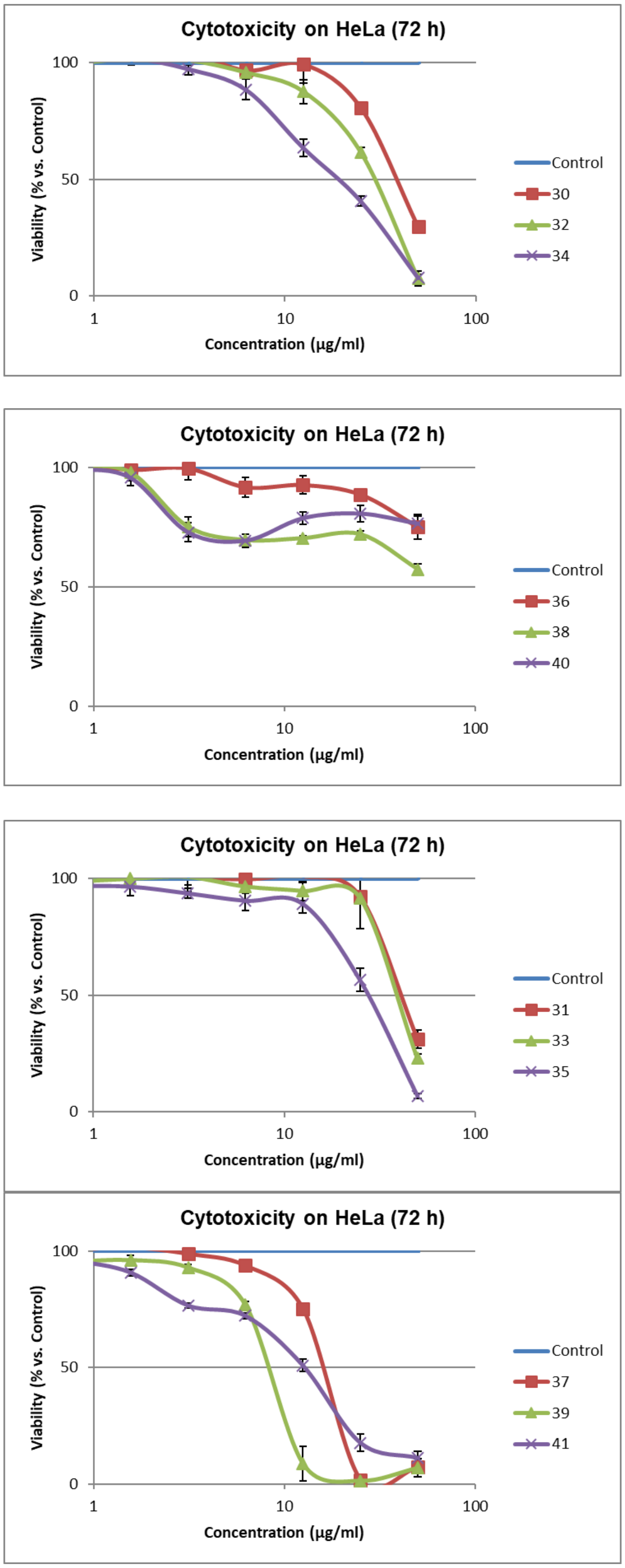

Figure SF5. Test for antiproliferative effects of compounds **30** – **41** on HeLa cells (72 h).

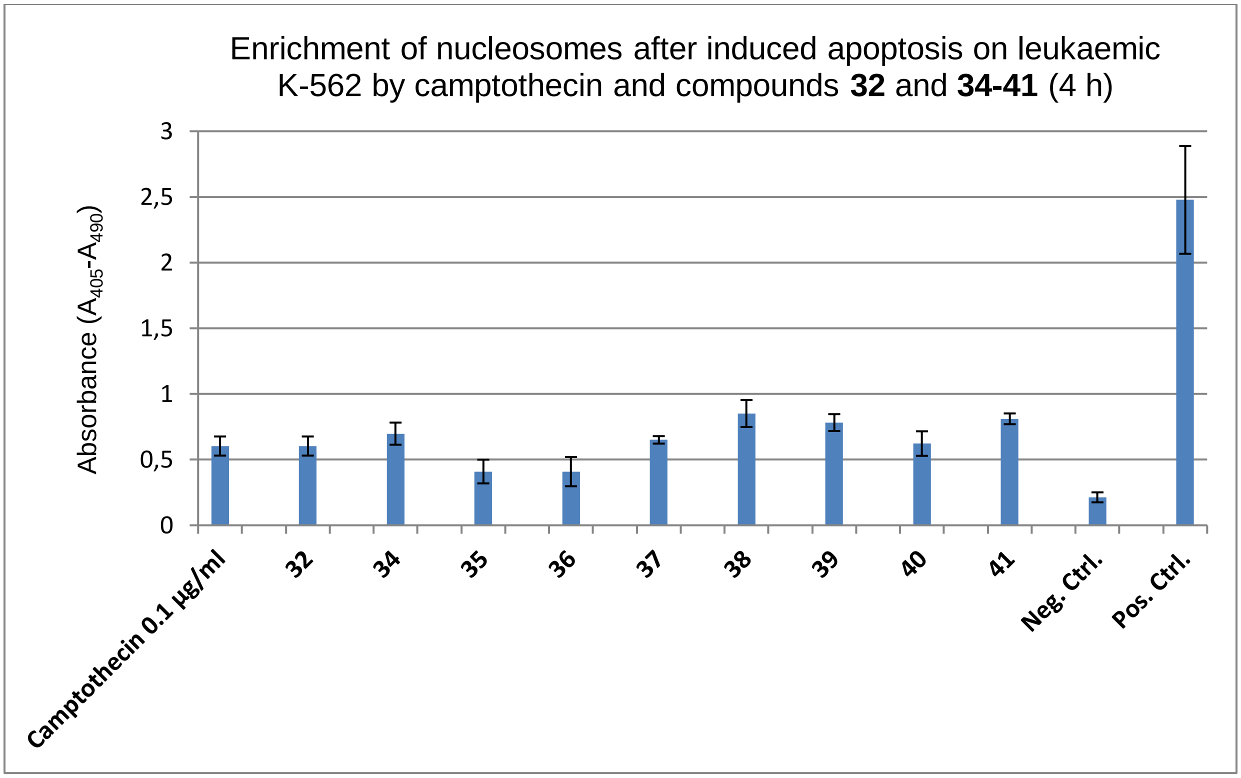

Figure SF6. Enrichment of nucleosomes after induced apoptosis on K-562 by camptothecin and compounds **32** and **34** - **41** (concentration of 10 μg/mL for tested compounds, after 4 h of exposure).

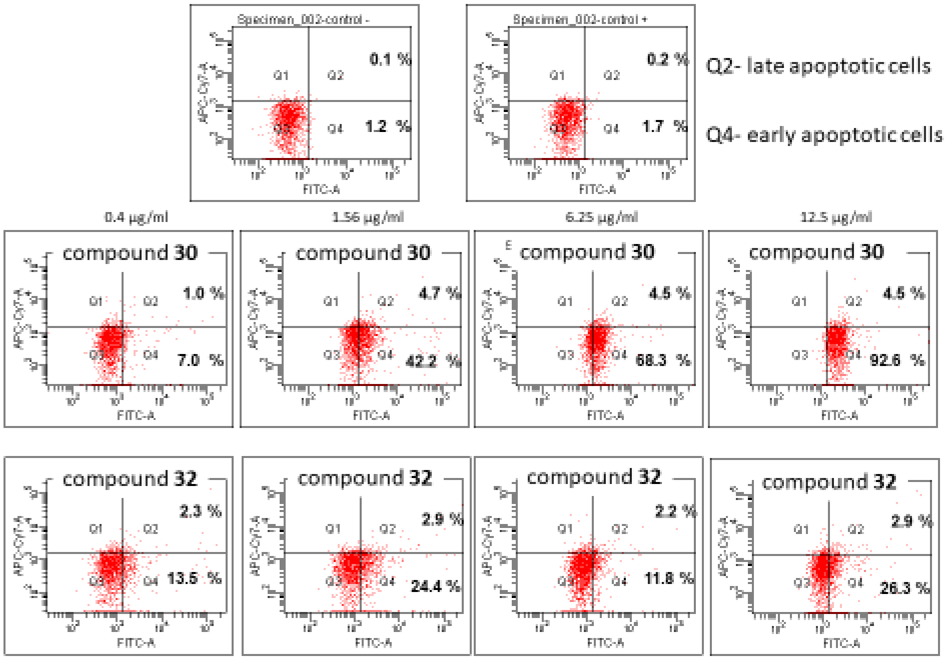
Specimen_002-control -
0.1 %
1.2 %
Specimen_002-control +
0.2 %
1.7 %
Q2- late apoptotic cells
Q4- early apoptotic cells
0.4 µg/ml
1.56 µg/ml
6.25 µg/ml
12.5 µg/ml
compound 30
1.0 %
7.0 %
compound 30
4.7 %
42.2 %
compound 30
4.5 %
68.3 %
compound 30
4.5 %
92.6 %
compound 32
2.3 %
13.5 %
compound 32
2.9 %
24.4 %
compound 32
2.2 %
11.8 %
compound 32
2.9 %
26.3 %
FITC-A
APC-Cy7-A

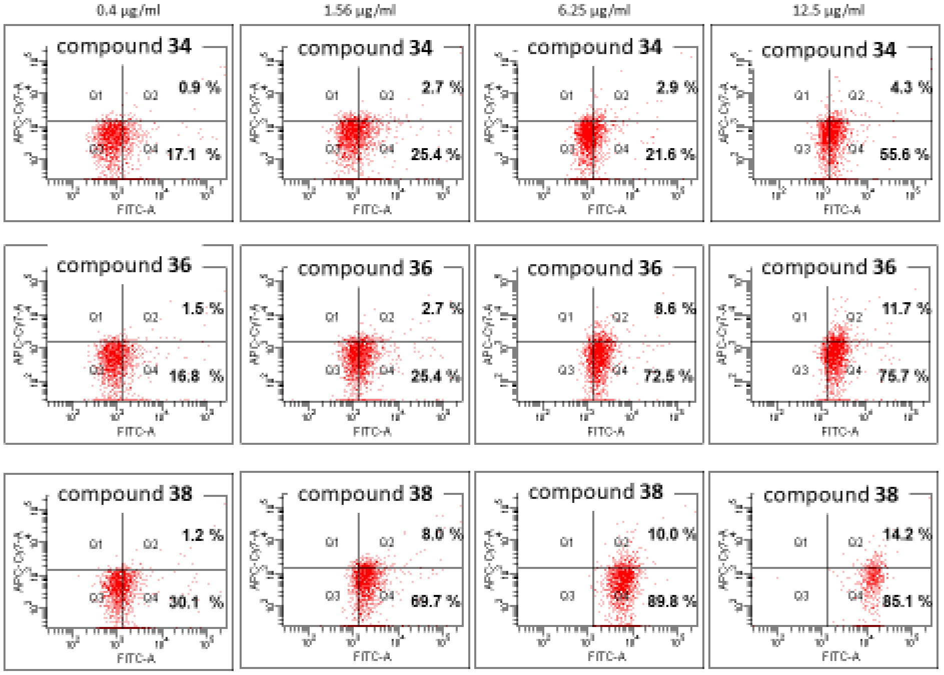
0.4 µg/ml
1.56 µg/ml
6.25 µg/ml
12.5 µg/ml
compound 34
0.9 %
17.1 %
compound 34
2.7 %
25.4 %
compound 34
2.9 %
21.6 %
compound 34
4.3 %
55.6 %
compound 36
1.5 %
16.8 %
compound 36
2.7 %
25.4 %
compound 36
8.6 %
72.5 %
compound 36
11.7 %
75.7 %
compound 38
1.2 %
30.1 %
compound 38
8.0 %
69.7 %
compound 38
10.0 %
89.8 %
compound 38
14.2 %
85.1 %
FITC-A
APC-Cy7-A

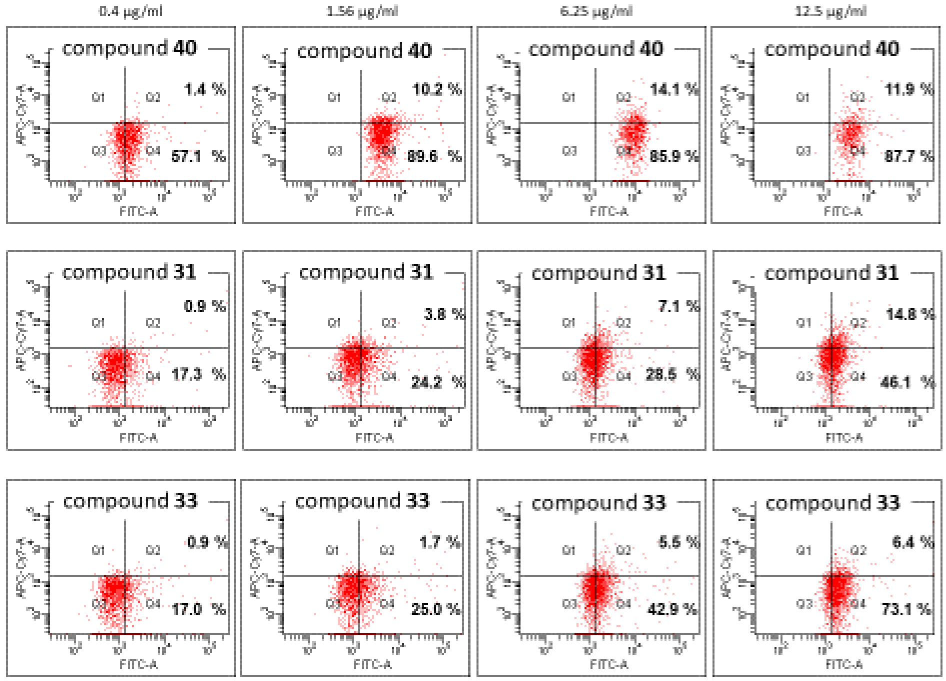
0.4 µg/ml
1.56 µg/ml
6.25 µg/ml
12.5 µg/ml
compound 40
1.4 %
57.1 %
compound 40
10.2 %
89.6 %
compound 40
14.1 %
85.9 %
compound 40
11.9 %
87.7 %
compound 31
0.9 %
17.3 %
compound 31
3.8 %
24.2 %
compound 31
7.1 %
28.5 %
compound 31
14.8 %
46.1 %
compound 33
0.9 %
17.0 %
compound 33
1.7 %
25.0 %
compound 33
5.5 %
42.9 %
compound 33
6.4 %
73.1 %
FITC-A
APC-Cy7-A

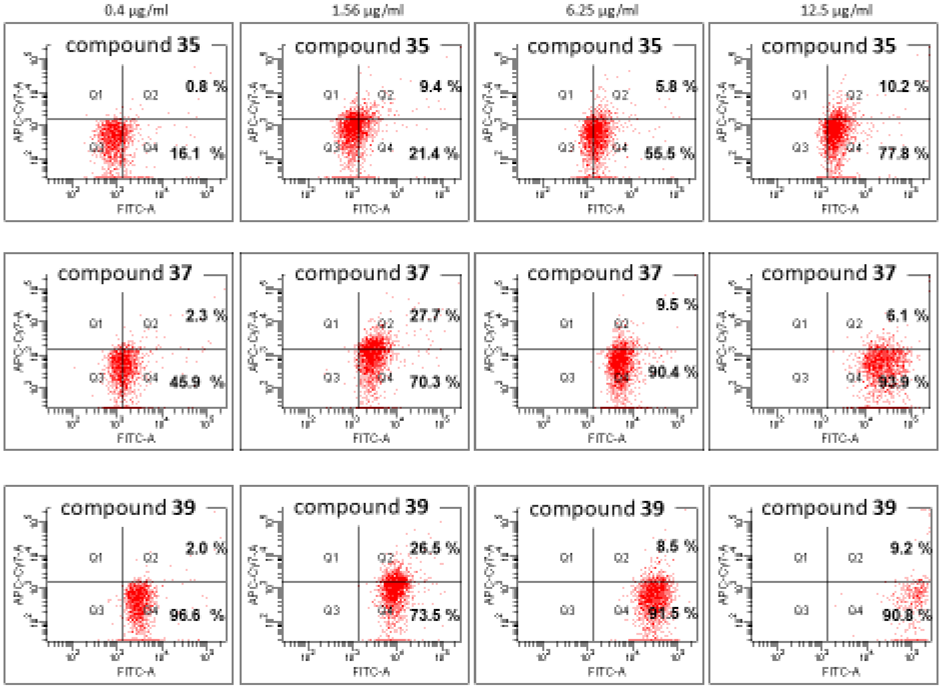
0.4 µg/ml
1.56 µg/ml
6.25 µg/ml
12.5 µg/ml
compound 35
0.8 %
16.1 %
compound 35
9.4 %
21.4 %
compound 35
5.8 %
55.5 %
compound 35
10.2 %
77.8 %
compound 37
2.3 %
45.9 %
compound 37
27.7 %
70.3 %
compound 37
9.5 %
90.4 %
compound 37
6.1 %
93.9 %
compound 39
2.0 %
96.6 %
compound 39
26.5 %
73.5 %
compound 39
8.5 %
91.5 %
compound 39
9.2 %
90.8 %
FITC-A
APC-Cy7-A

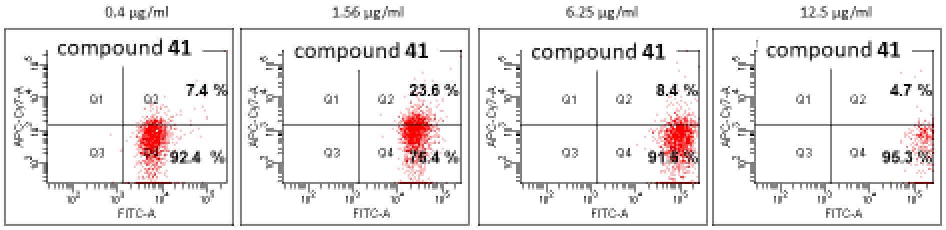

Figure SF7. Apoptosis detection for PBIQs 30-41 by flow cytometry. To detect the different stages of apoptosis within 24 h, we exploited the characteristics of apoptotic cells. During apoptosis, cells change their membrane structure; phosphatidylserine is then exposed on the outer side of the cells. Accordingly, it is possible to detect early apoptotic stages by annexin-V binding. Because the cell membrane becomes permeable in late apoptotic stages, DNA or intracellular free amine groups can be stained with viability dyes. Different staining protocols can thus be used to discriminate between early and late apoptotic cells. In the dot plots (see below), viable cells are depicted in quadrant 3 (Q3), early apoptotic cells in Q4, and late apoptotic cells in Q2.

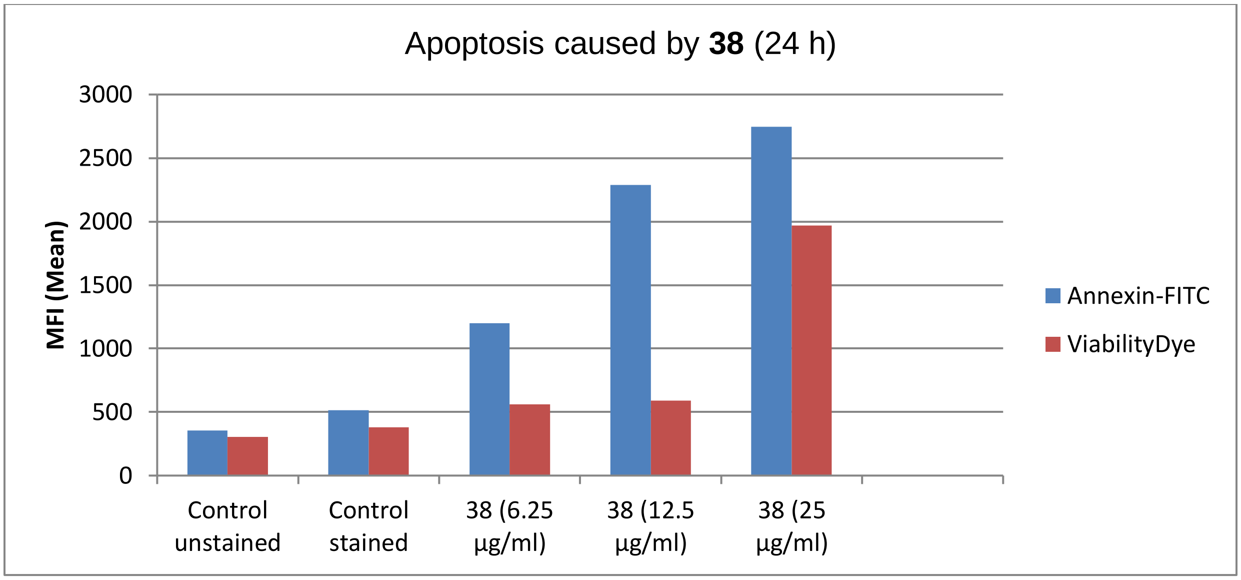

Figure SF8. Apoptosis progression for compound 38. Extracted fluorescence intensities after 24 h exposure at several concentrations with **38**. As apoptosis progresses, early and late apoptotic cells (**Fig. SF7**) can be seen to increase.

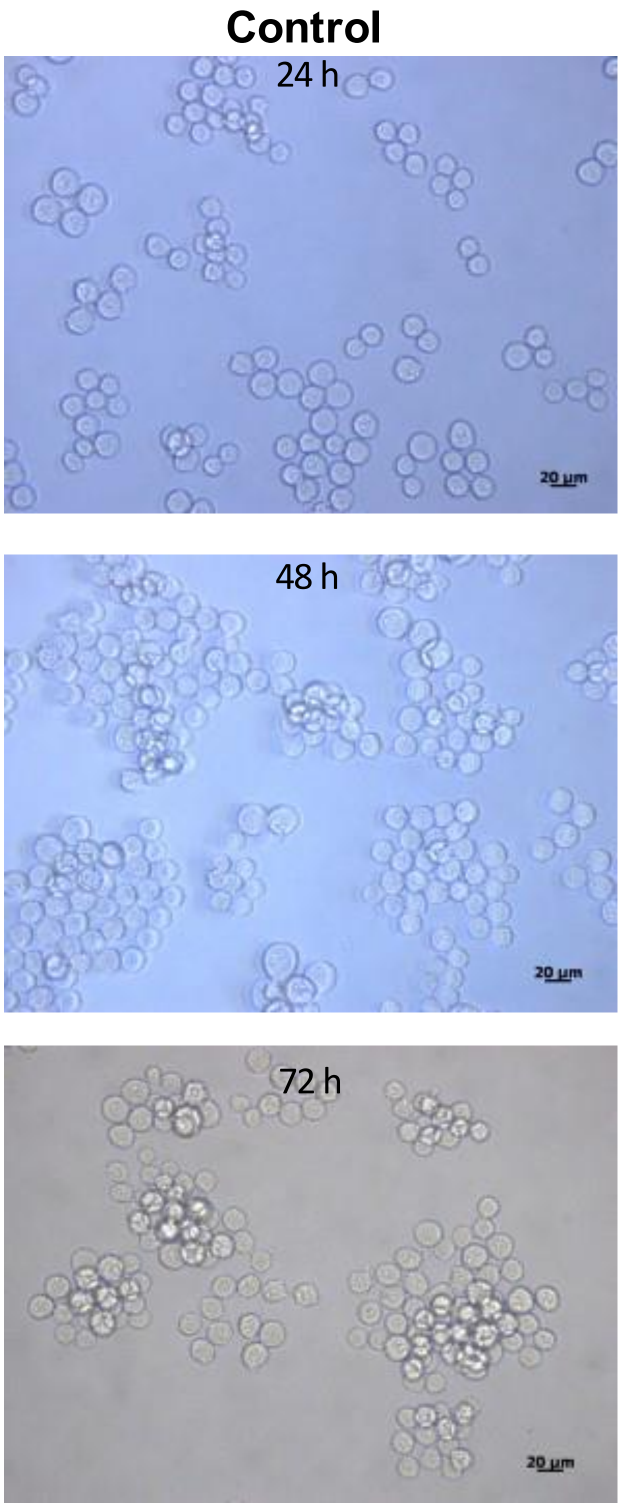

Figure SF9. Microscopic images of K-562 cells, control.

32 (6.2 μg/ml)

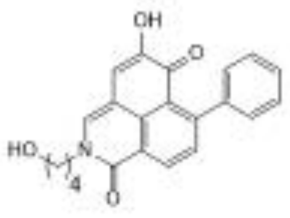

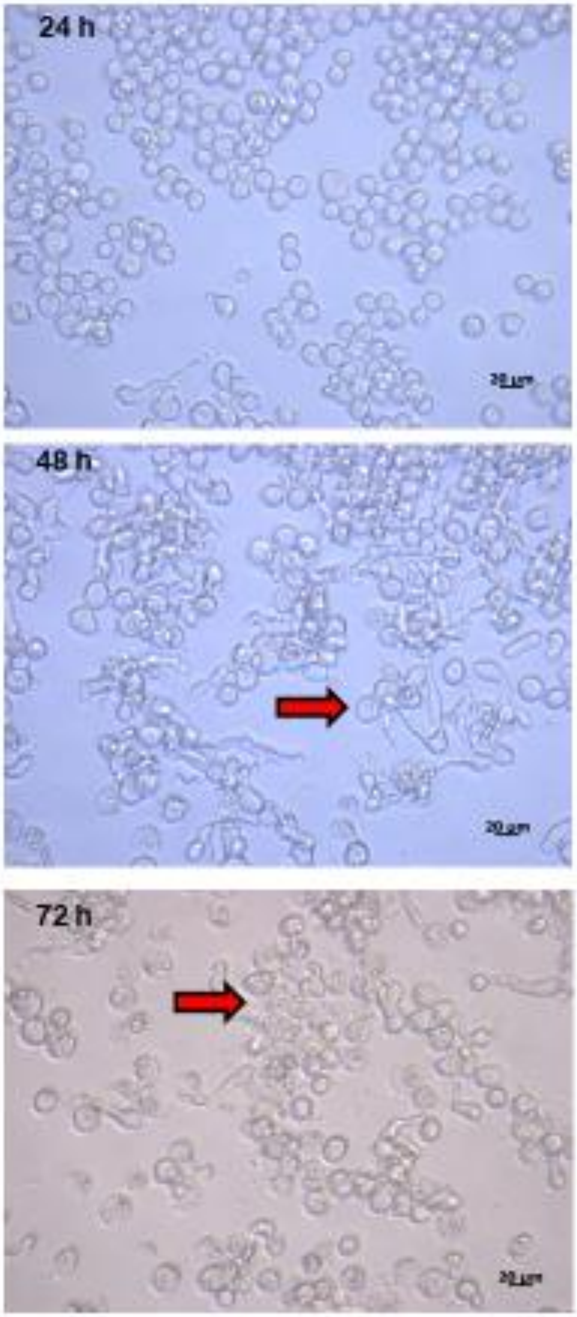

Figure SF10. Microscopic images of K-562 cells treated with **32**. Arrows indicate apoptotic cells.

34 (6.2 μg/ml)

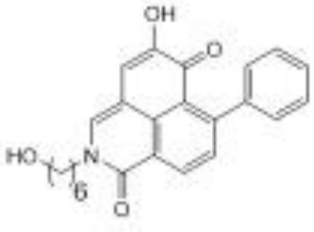

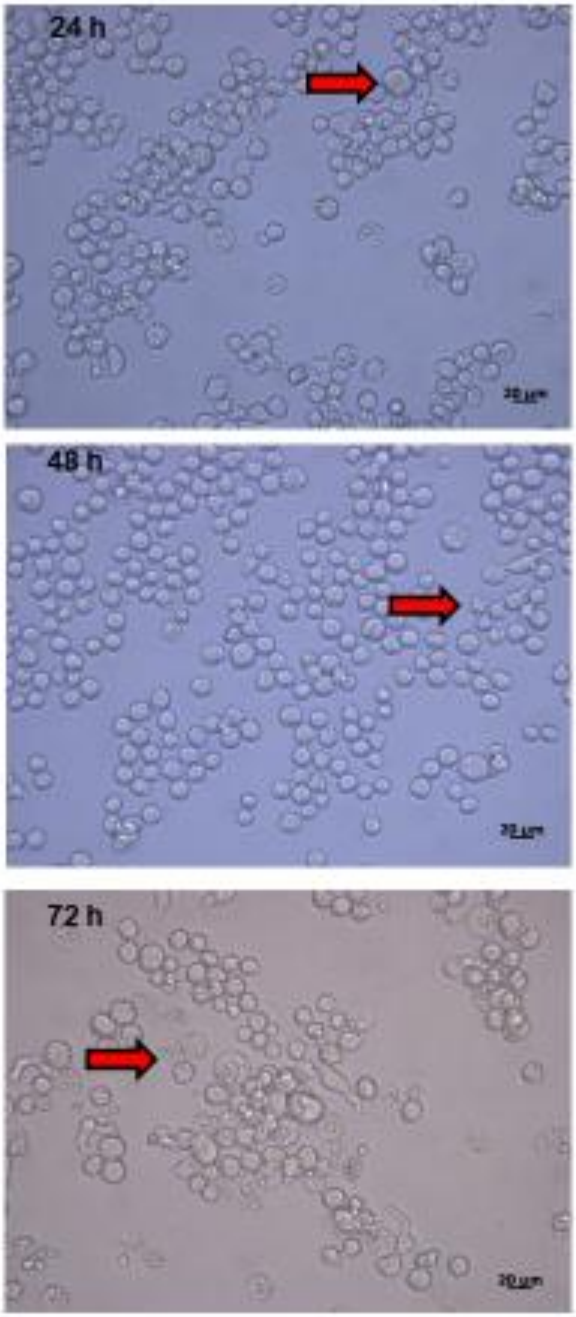

Figure SF11. Microscopic images of K-562 cells treated with **34**. Arrows indicate apoptotic cells.

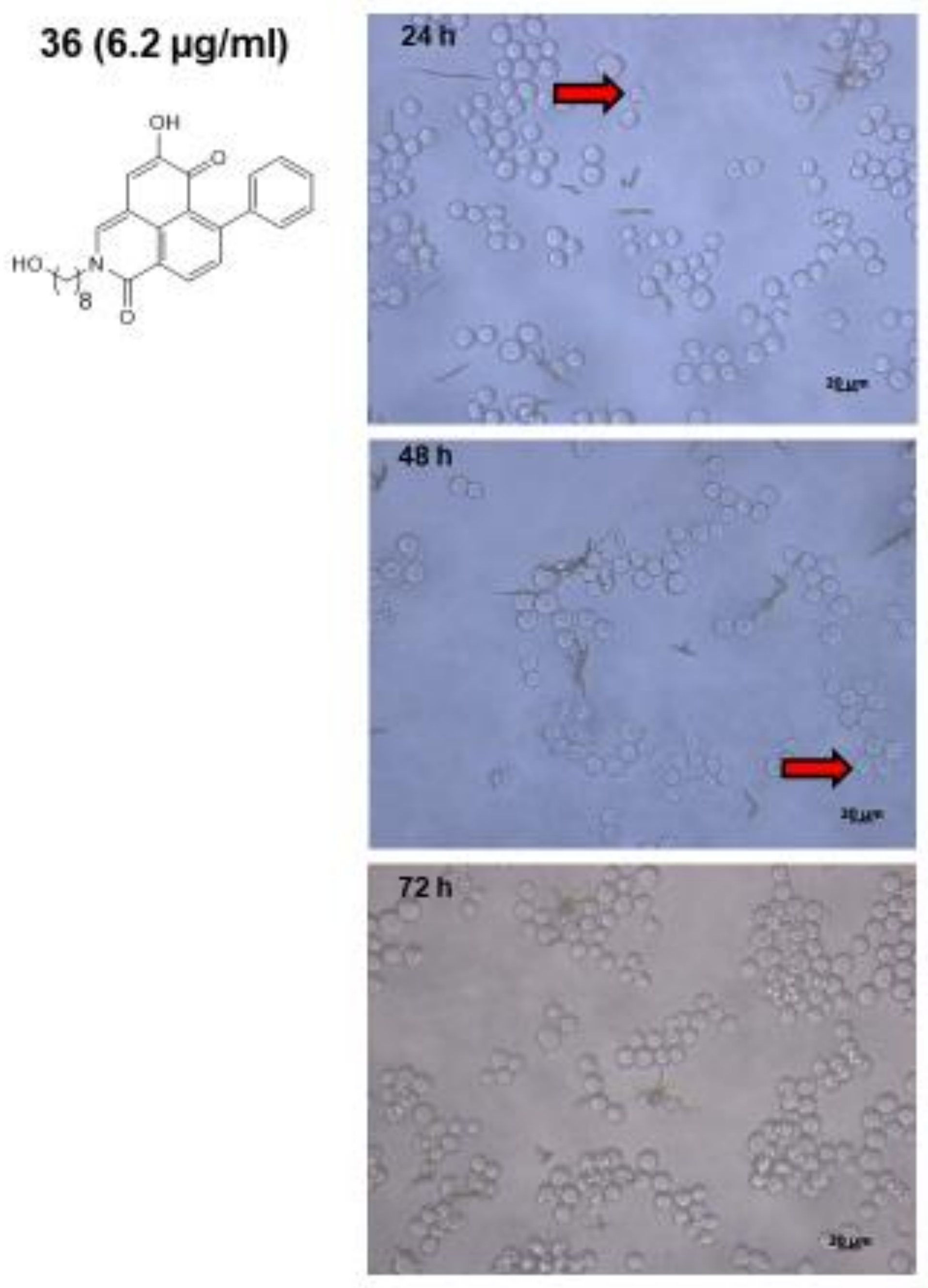

Figure SF12. Microscopic images of K-562 cells treated with **36**. Arrows indicate apoptotic cells.

37 (6.2 µg/ml)

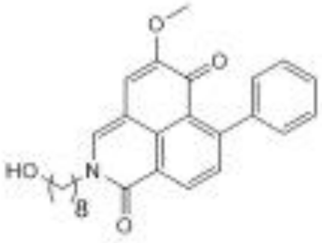

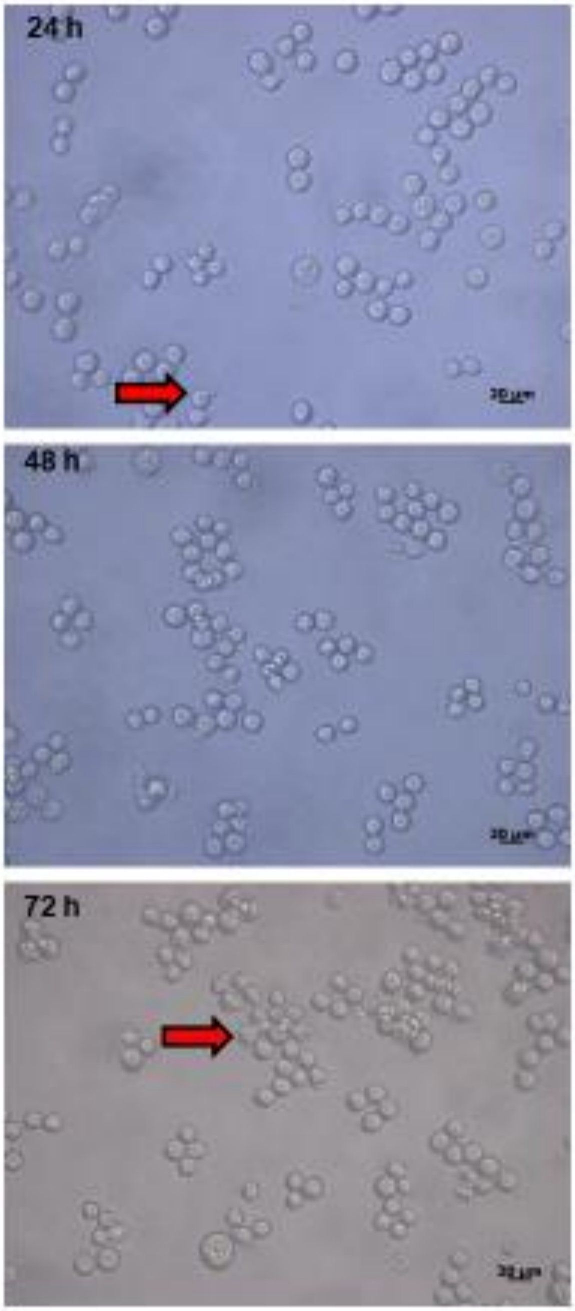

Figure SF13. Microscopic images of K-562 cells treated with **37**. Arrows indicate apoptotic cells.

38 (6.2 µg/ml)

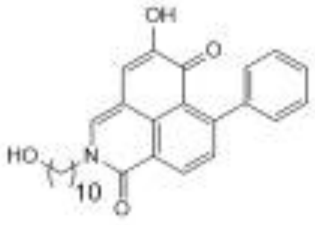

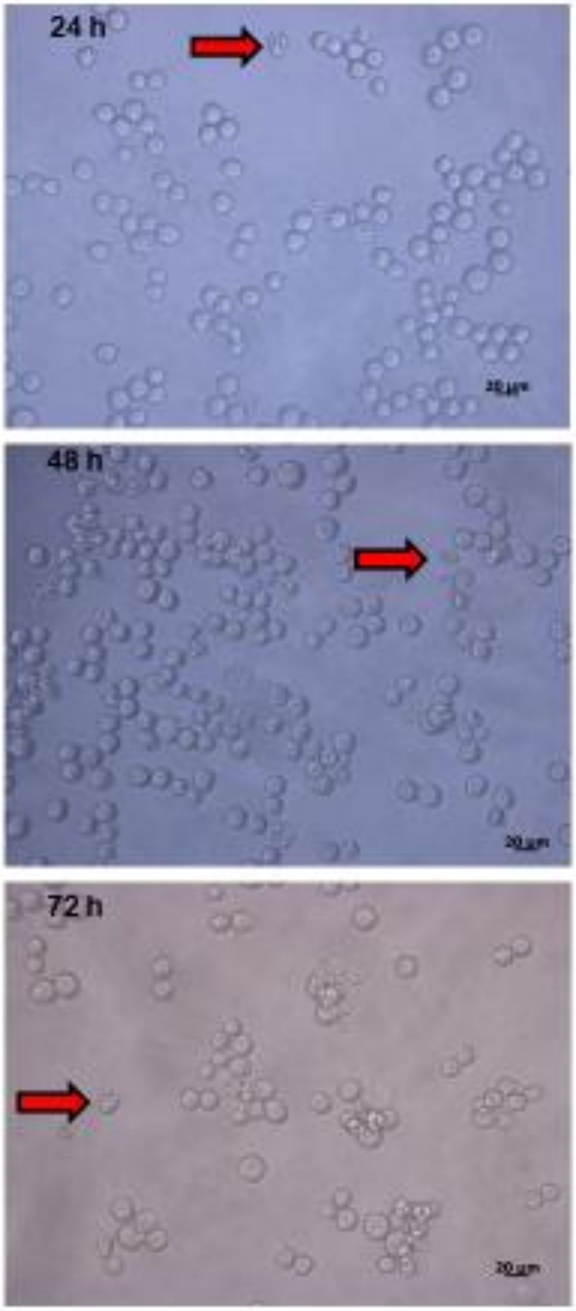

Figure SF14. Microscopic images of K-562 cells treated with **38**. Arrows indicate apoptotic cells.

39 (6.2 µg/ml)

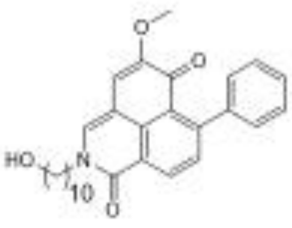

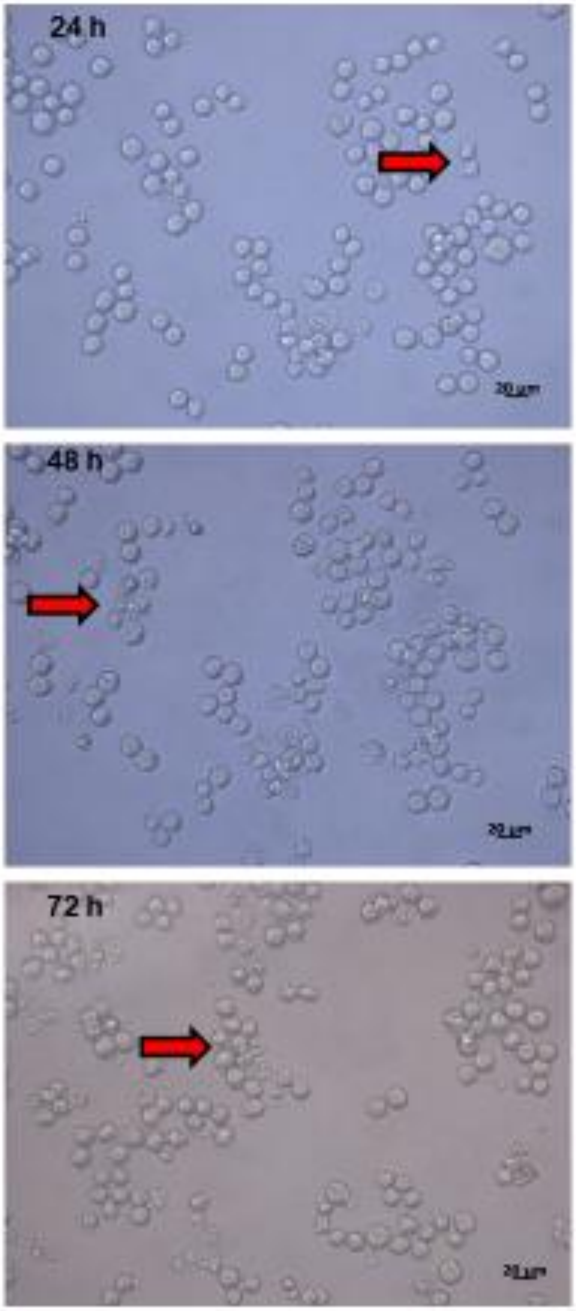

Figure SF15. Microscopic images of K-562 cells treated with **39**. Arrows indicate apoptotic cells.

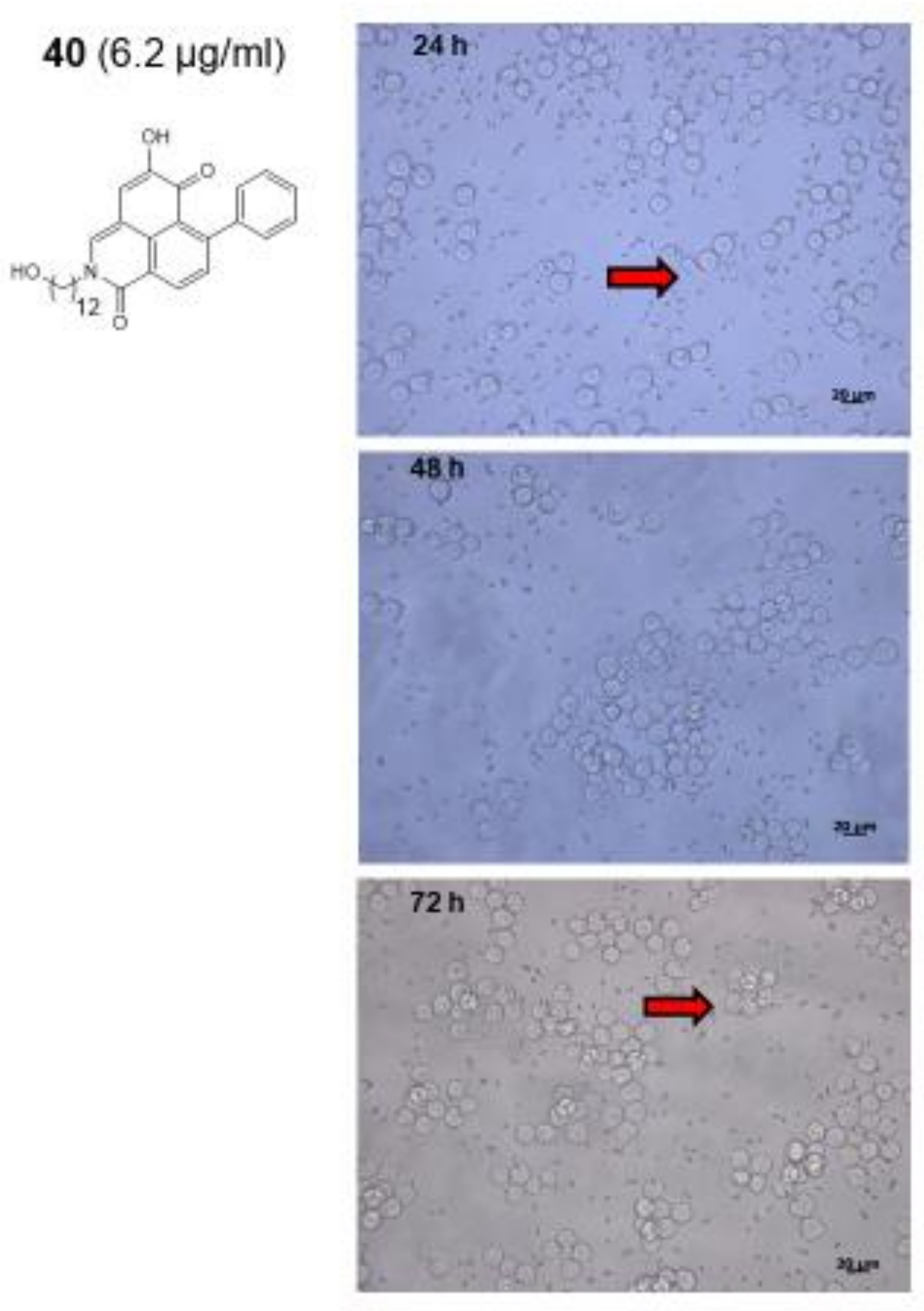

Figure SF16. Microscopic images of K-562 cells treated with **40**. Arrows indicate apoptotic cells

41 (6.2 µg/ml)

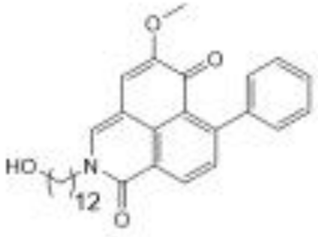

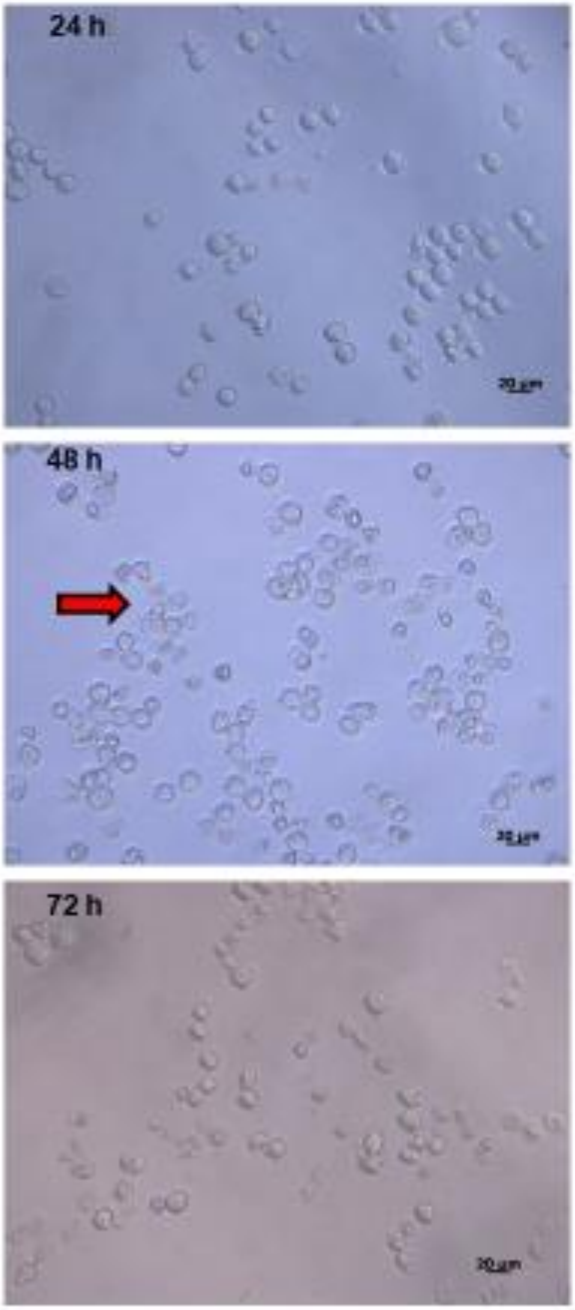

Figure SF17. Microscopic images of K-562 cells treated with **41**. Arrows indicate apoptotic cells.
